# Global, regional, and national disease burden estimates of acute lower respiratory infections due to respiratory syncytial virus in young children in 2015: a systematic review and modelling study

**DOI:** 10.1016/S0140-6736(17)30938-8

**Published:** 2017-09-02

**Authors:** Ting Shi, David A McAllister, Katherine L O'Brien, Eric A F Simoes, Shabir A Madhi, Bradford D Gessner, Fernando P Polack, Evelyn Balsells, Sozinho Acacio, Claudia Aguayo, Issifou Alassani, Asad Ali, Martin Antonio, Shally Awasthi, Juliet O Awori, Eduardo Azziz-Baumgartner, Henry C Baggett, Vicky L Baillie, Angel Balmaseda, Alfredo Barahona, Sudha Basnet, Quique Bassat, Wilma Basualdo, Godfrey Bigogo, Louis Bont, Robert F Breiman, W Abdullah Brooks, Shobha Broor, Nigel Bruce, Dana Bruden, Philippe Buchy, Stuart Campbell, Phyllis Carosone-Link, Mandeep Chadha, James Chipeta, Monidarin Chou, Wilfrido Clara, Cheryl Cohen, Elizabeth de Cuellar, Duc-Anh Dang, Budragchaagiin Dash-yandag, Maria Deloria-Knoll, Mukesh Dherani, Tekchheng Eap, Bernard E Ebruke, Marcela Echavarria, Carla Cecília de Freitas Lázaro Emediato, Rodrigo A Fasce, Daniel R Feikin, Luzhao Feng, Angela Gentile, Aubree Gordon, Doli Goswami, Sophie Goyet, Michelle Groome, Natasha Halasa, Siddhivinayak Hirve, Nusrat Homaira, Stephen R C Howie, Jorge Jara, Imane Jroundi, Cissy B Kartasasmita, Najwa Khuri-Bulos, Karen L Kotloff, Anand Krishnan, Romina Libster, Olga Lopez, Marilla G Lucero, Florencia Lucion, Socorro P Lupisan, Debora N Marcone, John P McCracken, Mario Mejia, Jennifer C Moisi, Joel M Montgomery, David P Moore, Cinta Moraleda, Jocelyn Moyes, Patrick Munywoki, Kuswandewi Mutyara, Mark P Nicol, D James Nokes, Pagbajabyn Nymadawa, Maria Tereza da Costa Oliveira, Histoshi Oshitani, Nitin Pandey, Gláucia Paranhos-Baccalà, Lia N Phillips, Valentina Sanchez Picot, Mustafizur Rahman, Mala Rakoto-Andrianarivelo, Zeba A Rasmussen, Barbara A Rath, Annick Robinson, Candice Romero, Graciela Russomando, Vahid Salimi, Pongpun Sawatwong, Nienke Scheltema, Brunhilde Schweiger, J Anthony G Scott, Phil Seidenberg, Kunling Shen, Rosalyn Singleton, Viviana Sotomayor, Tor A Strand, Agustinus Sutanto, Mariam Sylla, Milagritos D Tapia, Somsak Thamthitiwat, Elizabeth D Thomas, Rafal Tokarz, Claudia Turner, Marietjie Venter, Sunthareeya Waicharoen, Jianwei Wang, Wanitda Watthanaworawit, Lay-Myint Yoshida, Hongjie Yu, Heather J Zar, Harry Campbell, Harish Nair, RSV Global Epidemiology Network

**Affiliations:** a Centre for Global Health Research, Usher Institute of Population Health Sciences and Informatics, University of Edinburgh, Edinburgh, Scotland, UK; b Institute of Health and Wellbeing, University of Glasgow, Scotland, UK; c Department of International Health, International Vaccine Access Center, Johns Hopkins Bloomberg School of Public Health, Baltimore, MS, USA; d University of Colorado, Aurora, CO, USA; e Medical Research Council: Respiratory and Meningeal Pathogens Research Unit, School of Pathology, University of the Witwatersrand, Johannesburg, South Africa; f Department of Science and Technology/National Research Foundation: Vaccine Preventable Diseases, University of the Witwatersrand, Johannesburg, South Africa; g Agence de Medecine Preventive, Paris, France; h Fundacion INFANT, Buenos Aires, Argentina; i Centro de Investigação em Saúde de Manhiça (CISM), Maputo, Mozambique; j Epidemiology Department, Ministry of Health, Chile; k Ministry of Health, Togo; l Department of Pediatrics and Child Health, Aga Khan University, Pakistan; m Medical Research Council Unit The Gambia, Basse, The Gambia; n Department of Pediatrics, King George's Medical University, Lucknow (UP), India; o Kenya Medical Research Institute-Wellcome Trust Research Programme, Centre for Geographic Medicine Research-Coast, Kilifi, Kenya; p International Centre for Diarrhoeal Disease Research, Bangladesh; q Centers for Disease Control and Prevention, Atlanta, GA, USA; r Global Disease Detection Center, Thailand Ministry of Public Health—US Centers for Disease Control and Prevention Collaboration, Nonthaburi, Thailand; s Division of Global Health Protection, Centers for Disease Control and Prevention, Atlanta, GA, USA; t Ministry of Health, Nicaragua; u Hospital Materno Infantil Jose Domingo de Obaldia, Ciudad De David, Chiriqui, Panama; v Center for International Health, University of Bergen, Norway; w Department of Child Health, Tribhuvan University Institute of Medicine, Nepal; x ISGlobal, Barcelona Ctr Int Health Res (CRESIB), Hospital Clínic - Universitat de Barcelona, Barcelona, Spain; y ICREA, Pg Lluís Companys 23, 08010 Barcelona, Spain; z Hospital General Pediátrico Niños de Acosta Ñu, Ministerio de Salud Pública y Bienestar Social, San Lorenzo, Paraguay; aa Kenya Medical Research Institute, Centre for Global Health Research, Kisumu, Kenya; ab Wilhelmina Children's Hospital, University Medical Center Utrecht, The Netherlands; ac Emory Global Health Institute, Emory University, AT, USA; ad All India Institute of Medical Sciences, New Delhi, India; ae Department of Public Health and Policy, University of Liverpool, Liverpool, UK; af Arctic Investigations Program, National Center for Emerging and Zoonotic Infectious Diseases (NCEZID), Centres for Disease Control and Prevention, Anchorage, AK, USA; ag Institute Pasteur Cambodia, Children's Hospital Colorado, Aurora, CO, USA; ah GSK Vaccines Singapore, Children's Hospital Colorado, Aurora, CO, USA; ai Department of Pediatric Infectious Diseases, Children's Hospital Colorado, Aurora, CO, USA; aj National Institute of Virology, Pune, India; ak University Teaching Hospital, Lusaka, Zambia; al Rodolphe Merieux Laboratory, Faculty of Pharmacy, University of Health Sciences, Phnom Penh, Cambodia; am Centers for Disease Control and Prevention, Central American Region, Guatemala City, Guatemala; an Centre for Respiratory Diseases and Meningitis, National Institute for Communicable Diseases of the National Health Laboratory Service, Johannesburg, South Africa; ao School of Public Health, Faculty of Health Sciences, University of the Witwatersrand, Johannesburg, South Africa; ap Hospital San Juan de Dios, Santa Ana, Ministry of Health, El Salvador; aq National Institute of Hygiene and Epidemiology, Hanoi, Vietnam; ar Bayanzurkh District General Hospital, Ulaanbaatar, Mongolia; as Department of Pneumology, National Pediatric Hospital, Phnom Penh, Cambodia; at Centro de Educación Médica envestigaciones Clínicas “CEMIC”, Argentina; au Health Secretariat of the City of Belo Horizonte, Brazil; av Public Health Institute, Chile; aw Division of Infectious Disease, Key Laboratory of Surveillance and Early-warning on Infectious Disease, Chinese Center for Disease Control and Prevention, Beijing, China; ax Epidemiology Department, Austral University and Ricardo Gutiérrez Children Hospital, Argentina; ay Department of Epidemiology, School of Public Health, University of Michigan, Ann Arbor, MI, USA; az Vanderbilt University, Nashville, TN, USA; ba KEM Hospital Research Center, Pune, India; bb School of Women's and Children's Health, Faculty of Medicine, University of New South Wales, NSW, Australia; bc Department of Paediatrics, University of Auckland, Auckland, New Zealand; bd Centre for International Health, University of Otago, Dunedin, New Zealand; be Center for Health Studies, Universidad del Valle de Guatemala, Guatemala; bf Unit of Training and Research in Public Health, School of Medicine and Pharmacy of Rabat, University Mohamed V, Rabat, Morocco; bg University of Padjdjaran, Bandung, Indonesia; bh University of Jordan, Amman, Jordan; bi Department of Pediatrics and Medicine, Center for Vaccine Development, University of Maryland School of Medicine, Baltimore, MD, USA; bj National Scientific and Technical Research Council (CONICET), Buenos Aires, Argentina; bk Hospital Dr Ernesto Torres Galdames, Iquique, Chile; bl Research Institute for Tropical Medicine, Muntinlupa, Philippines; bm Research Institute for Tropical Medicine-Department of Health, Philippines; bn Ministry of Public Health and Social Welfare, Guatemala; bo Division of Global Health Protection, Center for Global Health, Centers for Disease Control and Prevention, Nairobi, Kenya; bp Pwani University, Kilifi, Kenya; bq Division of Medical Microbiology, University of Cape Town and National Health Laboratory Services, South Africa; br School of Life Sciences, University of Warwick, Coventry, UK; bs Mongolian Academy of Medical Sciences, Ulaanbaatar, Mongolia; bt Tohoku University Graduate School of Medicine, Department of Virology, Miyagi Prefecture, Japan; bu Emerging Pathofens Laboratory, Fondation Mérieux, Centre International de Recherche en Infectiologie (CIRI), Inserm U1111, CNRS UMR5308, ENS de Lyon, UCBL1, Lyon, France; bv Emory University, Rollins School of Public Health, AT, USA; bw Centre d'Infectiologie Charles Mérieux (CICM), Antananarivo, Madagascar; by Fogarty International Center Division of International Epidemiology and Population Studies, NIH, Bethesda, MD, USA; by Department of Pediatrics, Charité University Medical Center, Berlin, Germany; bz Hôpital Femme-Mère-Enfant, Antananarivo, Madagascar; ca United States Naval Medical Research Unit No. 6, Callao, Peru; cb Departamento de Biología Molecular y Genética, Instituto de Investigaciones en Ciencias de la Salud, Universidad Nacional de Asuncion, Paraguay; cc School of Public Health, Virology Department, Tehran University of Medical Sciences, Iran; cd Robert Koch Institute (RKI), Berlin, Germany; ce London School of Hygiene & Tropical Medicine, London, UK; cf Department of Emergency Medicine, University of New Mexico, Albuquerque, New Mexico, USA; cg Key Laboratory of Major Diseases in Children and National Key Discipline of Pediatrics (Capital Medical University), Ministry of Education, Beijing Pediatric Research Institute, Beijing Children's Hospital, Beijing, China; ch Alaska Native Tribal Health Consortium, Anchorage, AK, USA; ci Department of Research, Innlandet Hospital Trust, Lillehammer, Norway; cj Ministry of Health, Indonesia; ck CHU Gabriel Touré, Bamako, Mali; cl Centre for Infection and Immunity, Mailman School of Public Health, Columbia University, NY, USA; cm Shoklo Malaria Research Unit, Mahidol-Oxford Tropical Medicine Research Unit, Faculty of Tropical Medicine, Mahidol University, Mae Sot, Thailand; cn Centre for Viral Zoonosis, Department of Medical Virology, University of Pretoria, Pretoria, South Africa; co National Institute of Health, Department of Medical Sciences, Ministry of Public Health, Thailand; cp MOH Key Laboratory of Systems Biology of Pathogens and Christophe Mérieux Laboratory, IPB, CAMS-Fondation Mérieux, Institute of Pathogen Biology (IPB), Chinese Academy of Medical Sciences (CAMS) & Peking Union Medical College (PUMC), Beijing, China; cq Department of Pediatric Infectious Diseases, Institute of Tropical Medicine, Nagasaki University, Nagasaki, Japan; cr Department of Paediatrics and Child Heath, Red Cross War Memorial Children's Hospital and MRC Unit on Child & Adolescent Health, University of Cape Town, South Africa; cs Public Health Foundation of India, New Delhi, India

## Abstract

**Background:**

We have previously estimated that respiratory syncytial virus (RSV) was associated with 22% of all episodes of (severe) acute lower respiratory infection (ALRI) resulting in 55 000 to 199 000 deaths in children younger than 5 years in 2005. In the past 5 years, major research activity on RSV has yielded substantial new data from developing countries. With a considerably expanded dataset from a large international collaboration, we aimed to estimate the global incidence, hospital admission rate, and mortality from RSV-ALRI episodes in young children in 2015.

**Methods:**

We estimated the incidence and hospital admission rate of RSV-associated ALRI (RSV-ALRI) in children younger than 5 years stratified by age and World Bank income regions from a systematic review of studies published between Jan 1, 1995, and Dec 31, 2016, and unpublished data from 76 high quality population-based studies. We estimated the RSV-ALRI incidence for 132 developing countries using a risk factor-based model and 2015 population estimates. We estimated the in-hospital RSV-ALRI mortality by combining in-hospital case fatality ratios with hospital admission estimates from hospital-based (published and unpublished) studies. We also estimated overall RSV-ALRI mortality by identifying studies reporting monthly data for ALRI mortality in the community and RSV activity.

**Findings:**

We estimated that globally in 2015, 33·1 million (uncertainty range [UR] 21·6–50·3) episodes of RSV-ALRI, resulted in about 3·2 million (2·7–3·8) hospital admissions, and 59 600 (48 000–74 500) in-hospital deaths in children younger than 5 years. In children younger than 6 months, 1·4 million (UR 1·2–1·7) hospital admissions, and 27 300 (UR 20 700–36 200) in-hospital deaths were due to RSV-ALRI. We also estimated that the overall RSV-ALRI mortality could be as high as 118 200 (UR 94 600–149 400). Incidence and mortality varied substantially from year to year in any given population.

**Interpretation:**

Globally, RSV is a common cause of childhood ALRI and a major cause of hospital admissions in young children, resulting in a substantial burden on health-care services. About 45% of hospital admissions and in-hospital deaths due to RSV-ALRI occur in children younger than 6 months. An effective maternal RSV vaccine or monoclonal antibody could have a substantial effect on disease burden in this age group.

**Funding:**

The Bill & Melinda Gates Foundation.

**Research in context**

**Evidence before this study**

Respiratory syncytial virus (RSV) is the most common pathogen identified in young children with acute lower respiratory infections (ALRI), primarily pneumonia and bronchiolitis. Previously, we have estimated that in 2005, RSV was associated with 22% of all childhood ALRI episodes and 3–9% of all deaths. A substantial proportion of the RSV-associated morbidity occurred in the first year of life. We estimated that more than 93% of all RSV-ALRI episodes and 99% of RSV-ALRI mortality occurs in developing countries. However, these estimates were based on few data and were not stratified by narrow age bands for the first year of life. Global RSV-specific mortality estimates (using an alternative model) in young children, by the Institute of Health Metrics and Evaluation in Washington, have ranged from 234 000 (28% of all childhood ALRI) deaths in 2010 to 36 400 (5% of all childhood ALRI) deaths in 2015.

**Added value of this study**

This study used data from a vastly expanded dataset (329 studies of which 291 were not included in our previous estimates). Another important and unique feature is the use of unpublished data—23% of the included data are unpublished. We have now reported RSV-associated disease burden by severity and World Bank income region for narrow non-overlapping age bands particularly in the first year of life. We developed a risk-factor based model to provide the first estimates of RSV-ALRI burden in young children at national level. We estimated that RSV is associated with about 28% of all ALRI episodes and 13–22% of all ALRI mortality in young children. Using historical RSV case fatality data, we show that, in general, there has been a decreasing trend for RSV associated case fatality ratio across all age groups and income regions.

**Implications of all the available evidence**

There has been substantial reduction in child pneumonia morbidity and mortality in the past 15 years. With the introduction and scale-up of vaccines against leading bacterial pneumonia (Pneumococcus and *Haemophilus influenzae* type b), the proportional contribution of viral pathogens like RSV is likely to increase. In the past 5 years, there has been an unprecedented activity in RSV vaccine development. There are more than 60 candidate RSV vaccines in clinical development targeting pregnant women, neonates, and young children. WHO's Strategic Advisory Group of Experts on Immunization have identified absence of age-stratified disease burden estimates, data for RSV mortality in community, and burden data from Africa and south Asia as the key gaps in informing an evidence-based recommendation for the introduction of an RSV vaccine. Our findings should address some of these gaps and assist WHO, donor agencies, regulatory agencies, and policy makers to facilitate the introduction of a novel RSV vaccine in low-income and middle-income countries without delay.

## Introduction

Globally, acute lower respiratory infection (ALRI) remains one of the leading causes of morbidity and mortality in children younger than 5 years.^1^, ^2^ Human respiratory syncytial virus (RSV) is the most common viral pathogen identified in children with ALRI. We have previously estimated (from few data) that in 2005, about 33·8 million new episodes of RSV-ALRI occurred worldwide in young children, 10% severe enough to necessitate hospital admission.^3^ We also estimated that 55 000 to 199 000 child deaths could be attributed to RSV. Since then, many new RSV studies were initiated, collecting new data. Progress in RSV vaccines and therapeutics^4^ led WHO's Product Development for Vaccines Advisory Committee (PDVAC) to highlight RSV as “the most likely big new vaccine area with a vaccine likely to be available in the next 5 to 10 years”.^5^ Therefore, updated RSV disease burden estimates incorporating latest data are of great relevance for vaccine policy formulation and to prioritise research funding. We established the RSV Global Epidemiology Network (RSV GEN)—a collaboration of more than 70 investigator groups primarily in low-income and middle-income countries to estimate RSV-ALRI disease burden (at global, regional, and national levels) in young children for 2015; and highlight gaps in knowledge for future action.

## MethodsSystematic review

We did a systematic literature review (appendix pp 3–6), hand searching of online journals, and scanning reference lists of identified citations to update our previous review.^3^ The search included MEDLINE (Ovid), Embase, CINAHL, Global Health (1973 onwards), Global Health Library, Web of Science, IndMed, and grey literature (OpenGrey) databases and studies published between June 1, 2009, and Dec 31, 2016. Three authors (TS, EB, and SC) searched the literature (with no language or publication restrictions, and including three Chinese language databases [CNKI, Wanfang and ChongqingVIP for period 1/1/95-31/12/2016 (TS)] and extracted data independently (disagreements arbitrated and abstractions validated by HN).

We included studies reporting community incidence, hospital admissions, and in-hospital case fatality ratios (CFRs) for RSV confirmed ALRI in 0–4-year-old children. Studies with data for 12 or more consecutive months (except for mortality-related data), and those reporting RSV-ALRI incidence or mortality for the first year of life were reviewed. We excluded studies where RSV was not a primary outcome, and the case definition was not clear or inconsistently applied, RSV diagnosis was based on serology alone, or with less than 50 ALRI cases admitted to hospital.

RSV GEN formulated common case definitions and agreed on common approaches to data analysis (including re-analysis of already published data) and invited other investigators with relevant data to join RSV GEN. This resulted in analysis of substantial unpublished data to supplement published data (appendix pp 9–12). This study complies with the Guidelines for Accurate and Transparent Health Estimates Reporting (GATHER) recommendations (appendix p 95).^6^

### Definitions

As previously,^3^ we adapted WHO Integrated Management of Childhood Illnesses (IMCI)^7^ pneumonia case definitions to include RSV laboratory confirmation; and elected to replace “clinical pneumonia” and “severe pneumonia” with the terms “ALRI” and “severe ALRI” (appendix pp 2, 85). We recognised that WHO IMCI case definitions were developed for use by first level health workers, and for most hospital-based studies the decision for admission to hospital is based on physician's overall impression (and not IMCI signs alone). Therefore, we developed separate case definitions for hospital-based studies—admission to hospital for RSV-associated (severe or very severe) ALRI (appendix pp 2, 85). We expanded our definition for influenza seasonality to include RSV.^8^ Any month in which the virus was detected in more than 5% (at least 4) specimens was considered to be within RSV or influenza season. Industrialised and developing country designations followed UNICEF categories.^9^ We designated countries as high, upper-middle, lower-middle, and low-income based on the World Bank's classification. The child population estimates for 2015 are from UNPD World Population Prospects: 2015 revision.

### Statistical analysis

**Figure 1:**
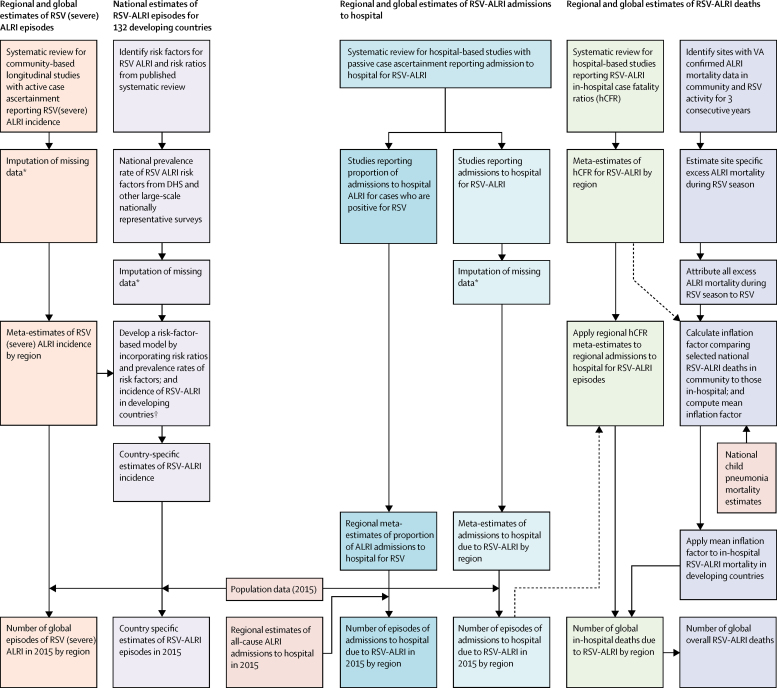
Approaches for estimation of global RSV associated morbidity and mortality in children aged 0–4 years In this study, we report four different sets of estimates—number of episodes of (severe) RSV-ALRI at global and national levels, global RSV-ALRI hospital admissions, and global estimates of RSV-ALRI deaths in hospital and overall (in community). This figure summarises our approach for each of these categories and also shows how they relate to (and feed into each other). Global estimates of hospital admissions for RSV-ALRI have been estimated using two independent approaches and datasets (after ensuring all included studies satisfy the common case definition that hospital admission was based on a physician diagnosis of ALRI). Similarly, the in-hospital deaths due to RSV-ALRI are based on studies reporting in-hospital CFR for RSV and RSV-ALRI hospital admissions (again ensuring that all included studies satisfy the common case definition). RSV=respiratory syncytial virus. ALRI=acute lower respiratory infection. hCFR=in-hospital case fatality ratio. VA=verbal autopsy. DHS=demographic and health survey. *For details description of imputation see appendix p 7. †For detailed description of risk-factor based model see appendix pp 54–57.

For studies not reporting 0–59 month incidence rates, we imputed any missing age group data using median incidence rate ratios (appendix p 7).^3^, ^8^, ^10^ We did a sensitivity analysis using un-imputed data and noted final estimates did not differ substantially. When the study was longer than 12 months, but not in multiples of 1 year, we calculated annualised incidence by adjusting for population at risk. If clinical specimens were systematically collected in a proportion of eligible cases and data for all eligible cases were available, we scaled results for proportion sampled. Figure 1 summarises and gives the rationale for our approach.

We did a data meta-analysis (by region and narrow age bands, where possible) for RSV and severe RSV-ALRI incidence, hospital admission rate for RSV-ALRI (studies with well-defined catchment population), proportion of hospital admissions for ALRI that were RSV positive (RSV+ve) and in-hospital RSV-ALRI CFR, and report pooled estimates (with 95% CI).^11^ Because in-study and between study data heterogeneity was anticipated, we used random effects models.^12^, ^13^ Incidence and hospital admission rate meta-estimates for RSV-ALRI and severe RSV-ALRI were applied to 0–5 year regional populations estimates to yield new episodes of RSV-ALRI and severe RSV-ALRI in 2015.

We validated hospital admissions for RSV-ALRI estimates with independent data by abstracting the proportion of ALRI hospital admissions that were RSV+ve. We then computed (WHO) regional proportion meta-estimates and applied these to regional estimates of hospital admissions for ALRI updated for 2015.^10^

We estimated RSV-ALRI episodes in young children in 132 developing countries using a risk-factor based model similar to that described previously.^1^ We calculated country level RSV-ALRI incidence using odds or rate ratios for six RSV risk factors [prematurity (<37 weeks), low birthweight (<2500 g), siblings, maternal smoking, HIV and crowding] from a meta-analysis of published studies,^14^ country-level risk factor prevalence (from relevant surveys and UN estimates), and estimates of RSV-ALRI incidence in developing countries. This assumes incidence in children without risk factors (unexposed rate) is similar within a region; that rate ratios can be multiplied when two or more are present; and that risk factors were independently distributed within countries (appendix pp 56–57).

We estimated in-hospital RSV-ALRI deaths by applying regional RSV-associated in-hospital CFR (hCFR) meta-estimates to regional number of RSV-ALRI hospital admissions (within narrow age bands; figure 1). We estimated in-hospital death uncertainty ranges (UR) using Monte Carlo Simulation (calculating estimates from 10 000 samples from log-normal distributions with 2·5th and 97·5th centiles defining the UR). We previously reported that about 80% of (all-cause) ALRI deaths in young children occur outside hospital.^10^ Therefore, to estimate overall RSV-associated deaths, we used the excess mortality model (as reported previously).^3^, ^8^ We identified sites with monthly death records (causes of death based on verbal autopsy, mortality surveys, and medical certification of deaths) with at least 100 ALRI community deaths over 3 consecutive years. We calculated the average number of ALRI community deaths per month during (AvgRSV) and outside (AvgOTHER) the RSV season, and the total number of deaths (TOTAL) during the year. We assumed that all excess ALRI mortality during RSV season was caused by RSV and that there is no RSV mortality outside RSV season. We defined the RSV season duration in months for every study year (MonRSV). The proportion of yearly deaths due to RSV was then estimated as:

$$\frac{(AvgRSV-AvgOTHER)\times Mon\;RSV}{TOTAL}$$

Because there is often some overlap in RSV and influenza seasonality, we calculated the area under the curve during RSV season and proportionately attributed excess ALRI mortality during RSV season to the two pathogens. Using published national estimates of 0–4 year ALRI mortality,^15^ we estimated RSV attributable ALRI mortality if community based case ascertainment was used. We then calculated the ratio between RSV-ALRI community and in-hospital deaths for each country to yield an “inflation factor”. Because the three inflation factors in these diverse developing country settings were similar, we assumed that these sites, and their inflation factors, are broadly representative of developing countries. We thus applied the mean inflation factor (for developing countries) to the estimated RSV-ALRI in-hospital deaths (in developing countries) to estimate the overall RSV-ALRI mortality for this region, and then calculated the “adjusted overall RSV mortality estimate” after accounting for overlap with influenza activity. We report all global and regional morbidity and mortality estimates to the nearest thousands of cases and hundreds of deaths. Country-specific results are reported without rounding.

Data were analysed with Stata version 11.2 and R version 3.0.2.

## Results

**Figure 2:**
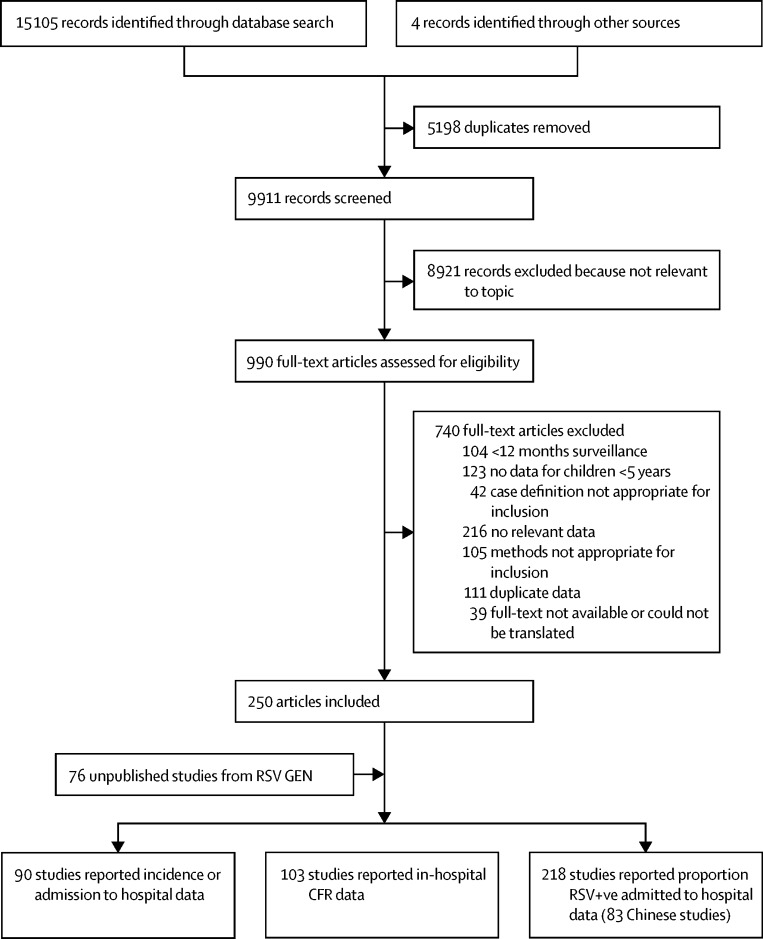
Flow diagram for selection of studies RSV=respiratory synctical virus. Studies could have contributed data to more than one category.

**Figure 3:**
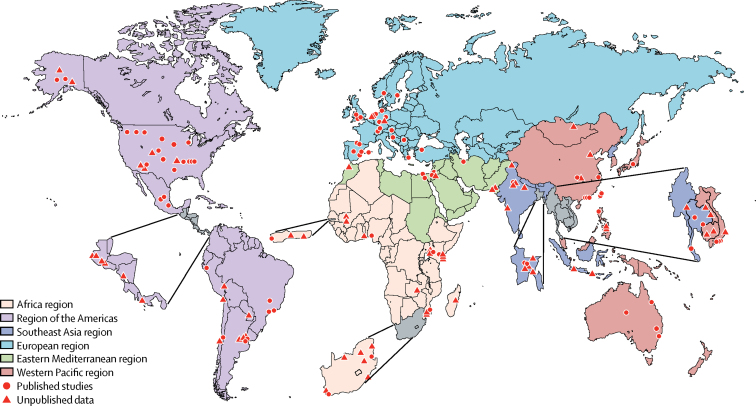
Location of studies reporting incidence, hospital admission, and in-hospital case fatality in children with RSV-ALRI RSV-ALRI=RSV-associated acute lower respiratory infection.

We identified 326 articles (329 studies) with data for community incidence, hospital admissions, in-hospital CFR, and proportion of hospital admissions for ALRI that are RSV+ve cases (figure 2); 250 were published (83 in Chinese) and 76 were unpublished (figure 3; appendix pp 9-12, 86). 41 studies were in rural, 250 in urban, and 38 in mixed populations. 30 (54%) of 56 developing country studies were either cohort or demographic surveillance site studies; and 26 (46%) were hospital studies with well-defined catchment populations. Only 40 studies (12 published and 28 unpublished) reported disease incidence or hospital admission rate by age group for the full age range; we imputed data in 51 studies (supplementary material pp 6–10). 63 studies (21%) reported the incidence or hospital admission rate or in-hospital CFR by narrow age bands for the first year of life. Only 37 studies (one published and 36 unpublished) reported data for neonates and only 19 studies by RSV sub-type.

Community-based studies with active case ascertainment reported RSV-ALRI incidence (14 studies), severe RSV-ALRI (eight studies) and very severe RSV-ALRI (four studies) in low-income and middle-income countries (LMICs; appendix pp 13–16); and an additional two studies reported incidence of RSV-ALRI outpatient clinic visits in high income countries. All but three studies reported peak RSV-ALRI incidence in children younger than 6 months (table 1; appendix pp 13–14).

**Table 1:** Estimates of the incidence, hospital admission rate, and number of episodes of RSV-ALRI in children younger than 5 years in 2015, by World Bank income regions and development status

		**Low income**	**Lower- middle income**	**Upper-middle income**	High income*	**Developing countries**	**Industrialised countries**	**Global**†
**RSV-ALRI**								
0–5 months								
	Studies‡	1 (1)	10 (2)	3 (1)	2 (2)	14 (4)	2 (2)	16 (6)
	Incidence¶ (uncertainity range)	117·2 (108·4–126·6)	63·3 (38·5–104)	168·9 (47·9–596·1)	66·1 (33·5–130·4)	82·5 (50·4–135·2)	66·1 (33·5–130·4)	..
	Number of episodes (thousands)	1247 (1153–1347)	2034 (1238–3344)	2991 (848–10555)	517 (262–1020)	5077 (3099–8318)	448 (227–884)	5560 (3570–8765)
6–11 months								
	Studies	0	8	2	0	10	0	..
	Incidence¶ (uncertainity range)	..	80·7 (48–135·6)	223 (95·2–522·1)	..	98·8 (58·8–166·1)	..	..
	Number of episodes (thousands)	..	2595 (1544–4361)	3948 (1686–9245)	..	6082 (3619–10 223)	..	..
0–59 months								
	Studies‡	1	10 (6)	3 (2)	2 (1)	14 (8)	2 (1)	16 (9)
	Incidence¶ (uncertainity range)	94 (89·1–99·1)	40·8 (25·7–65)	85·5 (33·8–216·7)	35·6 (16·6–76·2)	50·8 (32·4–79·7)	35·6 (16·6–76·2)	..
	Number of episodes (thousands)	9541 (9044–10 059)	12 864 (8081–20 478)	14 887 (5876–37 711)	2841 (1326–6090)	30 516 (19 463–47 853)	2482 (1158–5320)	33 059 (21 583–50 312)
RSV-associated severe ALRI**								
0-5 months								
	Studies‡	0	7 (2)	1	1 (1)	8 (2)	1 (1)	9 (3)
	Incidence¶ (uncertainity range)	..	25·1 (10·7–59·3)	406·7 (316·4–522·7)	3·2 (1·8–5·8)	36·1 (10·1–129·1)	3·2 (1·8–5·8)	..
	Number of episodes (thousands)	..	808 (343–1906)	7201 (5603–9255)	25 (14–45)	2222 (622–7945)	22 (12–39)	2174 (639–7470)
6–11 months								
	Studies	0	6	1	0	7	0	..
	Incidence¶ (uncertainity range)	..	19·5 (8·3–45·8)	82·1 (45·5–148·2)	..	24·7 (11·5–53·2)	..	..
	Number of episodes (thousands)	..	628 (268–1473)	1454 (805–2625)	..	1521 (707–3272)	..	..
0–59 months								
	Studies‡	0	7 (4)	1 (1)	1	8 (5)	1	9 (5)
	Incidence¶ (uncertainity range)	..	7·5 (3·1–18)	86·2 (68·4–108·6)	3 (1·7–5·5)	10·2 (3·5–29·9)	3 (1·7–5·5)	..
	Number of episodes (thousands)	..	2357 (980–5655)	15 003 (11 909–18 902)	243 (133–439)	6145 (2103–17943)	212 (117–383)	6303 (2317–18196)
**Hospital admission for RSV-associated ALRI**								
0–5 months								
	Studies‡	5 (2)	17 (8)	15 (9)	34 (25)	43 (22)	28 (22)	71 (44)
	Hospital admission rate	7·4 (2·4–22·6)	22·9 (17·7–29·7)	23·0 (16·1–32·9)	26·3 (22·8–30·2)	20·2 (16·7–24·5)	27·1 (23·3–31·6)	..
	Number of episodes (thousands)	79 (26–240)	737 (569–955)	407 (284–582)	205 (178–237)	1243 (1025–1508)	184 (158–214)	1447 (1204–1744)
6–11 months								
	Studies	4	9	5	9	20	7	27
	Hospital admission rate	3·4 (0·6–19·5)	11·3 (6·1–21·0)	18·5 (9·8–34·7)	11·3 (6·1–20·9)	11·0 (7·7–15·7)	9·8 (4·8–19·6)	..
	Number of episodes (thousands)	36 (6–207)	362 (195–674)	327 (174–615)	88 (48–163)	674 (471–963)	66 (33–133)	849 (575–1275)
12–59 months								
	Studies	3	9	7	7	21	5	26
	Hospital admission rate	0·4 (0·1–1·7)	1·8 (1·2–2·8)	2·2 (1·3–3·9)	1·4 (0·9–2·0)	1·5 (1·0–2·1)	1·6 (1·0–2·5)	..
	Number of episodes (thousands)	30 (7–132)	451 (288–702)	305 (173–538)	87 (60–128)	693 (482–1003)	90 (59–140)	897 (660–1236)
0–59 months								
	Number of episodes (thousands)†	168 (73–410)	1575 (1252–1998)	1065 (787–1450)	383 (322–467)	2629 (2238–3102)	344 (285–427)	3216 (2745–3801)
**RSV-associated hospital admission ALRI with hypoxaemia**								
0–5 months								
	Studies	3	2	5	0	10	0	..
	Hospital admission rate	6·1 (3·0–12·6)	7·1 (2·8–18·1)	11·9 (4·3–33·3)	..	8·9 (4·4–18·0)	..	..
	Number of episodes (thousands)	65 (32–134)	227 (88–581)	210 (75–589)	..	548 (272–1107)	..	..
6–11 months								
	Studies	3	2	5	0	10	0	..
	Hospital admission rate	2·1 (0·5–8·7)	5·8 (3·1–11·0)	4·6 (1·3–16·1)	..	3·8 (1·9–7·6)	..	..
	Number of episodes (thousands)	23 (6–93)	186 (98–355)	81 (23–285)	..	234 (118–469)	..	..
12–59 months								
	Studies	3	0	3	1	6	1	7
	Hospital admission rate	0·1 (0·1–0·3)	..	0·4 (0·1–1·5)	1·8 (1·8–1·9)	0·3 (0·1–0·8)	1·8 (1·8–1·9)	..
	Number of episodes (thousands)	11 (5–27)	..	55 (14–211)	118 (113–121)	129 (48–363)	103 (99–106)	232 (149–458)
0–59 months								
	Number of episodes (thousands)†	105 (59–196)	..	383 (187–810)	..	951 (595–1588)	..	..

RSV=respiratory syncytial virus. ALRI=acute lower respiratory infection. Incidence and hospital admission rate are presented as per 1000 children per year. Incidence, hospital admission rate, and number of episodes are presented with 95% CI.

* Excludes studies in aboriginal populations in high-income countries.

† Although the overall number of cases was obtained by summing the age and region-specific numbers for each of the 10 000 samples in the Monte Carlo simulation, the point estimates and uncertainty interval limits for the overall cases are not equal to the sum of the age and region-specific results. This reflects the fact that the overall estimates are determined by the full uncertainty distributions for each age and region-specific estimates, and not simply the point estimates.

‡ Data in parentheses indicate number of studies with imputed data.

¶ Incidence estimates (in any age group) are per 1000 children (in that age group) per year.

** This is a subset of RSV-ALRI (see appendix p 85).

We estimated that 30·0 million (95% CI 19·1–47·0) RSV-ALRI episodes occurred in 0–4-year-old children in LMIC in 2015, about a third in the first year of life. An estimated 2·8 million (95% CI 1·3–6·1) RSV-ALRI episodes occurred in high-income countries. Therefore, globally, we estimate 33·1 million (UR 21·6–50·3) RSV-ALRI episodes in young children in 2015. Few data from three (of 14) community based studies indicate a high incidence rate, even in the neonatal period—40 (95% CI 2·5–635·7) episodes per 1000 neonates per year (appendix p 42).

About 20% of (community) cases in young children had lower chest wall indrawing (severe RSV-ALRI); a third observed in infants (table 1, appendix p 43). We also estimated the incidence and number of RSV-ALRI episodes in young children in 132 LMICs in 2015. Despite a wide range of incidence rates from 65·6 (UR 40·3–105·1) per 1000 children per year in Senegal to 31·0 (18·7–50·8) in China, there was only a limited variation in point estimates with very wide uncertainty ranges for most countries (appendix p 87). Five countries (with about 43% of global under-5 children)—India, China, Nigeria, Pakistan, and Indonesia—contributed about half the global RSV-ALRI burden (appendix pp 58–61).

76 hospital-based studies (five in indigenous populations) with passive case ascertainment reported hospital admission rates for RSV-ALRI for young children (appendix pp 17–22). Across all regions, hospital admission rates were highest in infants younger than 6 months. Hospital admission rates were also high in the neonatal period—15·9 (95% CI 8·8–28·9) hospital admissions per 1000 neonates per year in developing countries (appendix p 44). There were relatively few studies reporting hospital admissions for RSV-ALRI in low-income countries and their hospital admission (across all age groups) were much lower than the highest rates (in upper-middle-income countries). We estimated 3·2 million (UR 2·7–3·8) hospital admissions for RSV-ALRI occurred globally in young children in 2015; about 45% of these in children aged younger than 6 months (table 1).

Of the 218 hospital-based studies (without clear population denominator) that reported proportion of RSV+ve cases among all hospital admissions for ALRI, only 104 studies reported 0–59 month data (appendix pp 23–32). Using this independent dataset we estimated that about 2·9 million (95% CI 2·6–3·3) hospital admissions for RSV-ALRI occurred in young children in 2015 (appendix pp 46–47). About 85% of all hospital admission cases had chest wall indrawing (data not shown). 28 (61%) of 46 studies recording SpO_2_ by pulse oximetry used our hypoxaemia case definition and these reported about 20% of all hospital admissions for RSV-ALRI cases aged 0–4 years (all age groups) had hypoxaemia (appendix pp 45, 83–84). This translates to about 1·0 million (UR 0·6–1·6) episodes of hospital admissions for severe RSV-ALRI with hypoxaemia in young children from developing countries, 58% in infants younger than 6 months. We also estimated 0·6 million (UR 0·4–1·1) hospital admissions for very severe RSV-ALRI in young children in developing countries in 2015, 51% in infants younger than 6 months.

Data were insufficient to provide global incidence or hospital admissions by RSV subtype. RSV-A was the more common circulating subtype and resulted in more severe disease (with substantially higher hospital admissions and hCFR) across all age groups (appendix p 48).

48 published and 55 unpublished studies reported hCFR data for young children with RSV-ALRI (appendix pp 33–38). We identified 41 studies from developing countries that reported RSV-associated hCFR in children aged 0–5 months, 6–11 months, and 12–59 months (table 2; appendix pp 39-41, 63). Overall, the highest hCFR was observed in neonates [5·3% (95% CI 2·8–9·8)] and in children from low-income countries. This translates to a substantial in-hospital mortality of about 59 600 deaths (UR 48 000–74 500) in children younger than 5 years globally, 46% occurring in infants younger than 6 months. Because this estimate only includes children admitted to hospital, it is an underestimate due to limited access to care and poor care seeking in LMICs.^10^

**Table 2:** CFR meta-estimates and number of in-hospital deaths in children with RSV-ALRI in children younger than 5 years in 2015, by World Bank Income regions

		**Low income**	**Lower-middle income**	**Upper-middle income**	**High income**	**Developing countries**	**Industrialised countries**	Global*†
Studies		9	16	12	6	41	2	43
0–5 months								
	hCFR (%)‡	1·7 (0·4–6·8)	2·7 (2·0–3·6)	1·8 (1·2–2·6)	0·2 (0·0–12·8)	2·2 (1·8–2·7)	0·0 (0·0–0·1)	..
	Number of deaths‡§	1300 (200–7900)	20 000 (13 500–29 500)	7200 (4200–12 300)	400 (1–228 200)	27 100 (20 700–35 500)	<50 (0–2000)	27 300 (20 700–36 200)
6–11 months								
	hCFR (%)‡	9·3 (3·0–28·7)	2·8 (1·8–4·4)	2·4 (1·1–5·4)	0·9 (0·2–4·0)	2·4 (1·9–3·2)	0·1 (0·0–0·4)	..
	Number of deaths§‡	3400 (400–26 600)	10 300 (4800–21 600)	8000 (2800–22 100)	900 (200–4600)	16 500 (10 400–25 800)	<50 (0–300)	16 500 (10 500–26 100)
12–59 months								
	hCFR (%)‡	4·7 (0·7–33·7)	2·7 (1·7–4·3)	0·5 (0·1–3·5)	0·7 (0·1–5·2)	2·2 (1·6–3·0)	0·1 (0·0–0·3)	..
	Number of deaths§‡	1400 (100–16 100)	12 300 (6500–23 100)	1500 (200–11 700)	700 (100–5600)	15 300 (9500–25 000)	100 (0–300)	15 400 (9500–24 900)
0–59 months								
	Number of deaths‡§	8200 (2200–36 900)	43 600 (31 400–60 400)	17 900 (10 300–34 500)	3300 (700–231 100)	59 600 (47 800–74 300)	200 (100–2200)	59 600 (48 000–74 500)

RSV=respiratory syncytial virus. ALRI=acute lower respiratory infection. hCFR=in-hospital CFR. hCFR and number of deaths are presented with 95% CI.

* Global total for a given age band is sum of the deaths in developing and industrialised countries. We have taken this more conservative approach because there are only a small number of studies contributing to deaths by World Bank income region in narrow age bands leading to large uncertainties in some of these estimates.

† Although the overall number of deaths was obtained by summing the age and region-specific numbers for each of the 10 000 samples in the Monte Carlo simulation, the point estimates and uncertainty interval limits for the overall deaths are not equal to the sum of the age and region-specific results. This reflects the fact that the overall estimates are determined by the full uncertainty distributions for each age and region-specific estimates, and not simply the point estimates.

‡ Data in parentheses are 95% CI.

§ The number of deaths has been rounded to the nearest hundreds.

To estimate the overall RSV-ALRI deaths in young children (including those dying outside hospitals), we identified eight LMIC sites that could provide requisite data. However, data from only three sites (multiple villages across rural Bangladesh, urban slums in Buenos Aires, and multiple hamlets in Lombok, Indonesia) met our strict eligibility criteria. Data for both RSV and influenza activity were available from Bangladesh and Buenos Aires. In Bangladesh (after excluding 2010 influenza data which overlapped with second wave of influenza A (H1N1) pdm09 virus pandemic), there was some overlap between RSV and influenza activity during RSV season. We estimated that about 90% (range 86–93) of excess mortality during RSV season can be attributed to RSV (appendix p 64). There was no overlap between RSV and influenza seasons in Buenos Aires in the years studied.^16^ The “inflation factors” ranged from 1·5 in Argentina to 2·9 in Lombok (appendix p 65). We “adjusted” our estimates for overall RSV-ALRI mortality in developing countries to account for influenza activity during RSV season and estimated that the global RSV-ALRI mortality in young children in 2015 was 118 200 (UR 94 600–149 400). Available morbidity and mortality data show substantial yearly variation in RSV activity and associated ALRI deaths (appendix pp 89–93), suggesting that national, regional, and global RSV morbidity and mortality vary substantially from year to year.

## Discussion

Our revised RSV burden estimates are based on 329 studies (291 of which were not included in our 2005 estimates). We estimate that globally in 2015 there were about 33·1 million (UR 21·6 −50·3) RSV-ALRI episodes resulting in about 3·2 (UR 2·7–3·8) million hospital admissions, and 59 600 (48 000–74 500) in-hospital deaths in (670·5 million) children younger than 5 years. A plausibility check using an independent approach with non-overlapping data from 218 different studies was in good agreement and supports the validity of the hospital admission estimates. The proportion of eligible cases that were tested for RSV varied substantially (appendix pp 49-53). Because the most common reasons for not collecting specimens for testing were death, discharge, absence of parental consent or the child being too ill, studies might have underestimated in-hospital mortality estimates. Consistent with this, hCFR among those not tested was substantially higher than those tested for RSV (appendix pp 67–69). We did several sensitivity analyses considering various scenarios (if RSV positivity in the untested were the same as that in those tested; and if none or all of the deaths in untested cases are RSV positive), suggesting that the overall in-hospital RSV-ALRI mortality estimates could increase by 7–40% (appendix pp 70–79). If the in-hospital mortality estimates are based on a subset of 22 studies that reported RSV data by narrower age bands (including the neonatal period), then the in-hospital mortality estimates would increase by 36% (appendix p 80). Our in-hospital RSV-ALRI mortality estimates is based on the maximum number of eligible datapoints. Consistent with this, our hCFR estimates for RSV-ALRI are substantially lower than those estimated for all-cause hospital admissions for ALRI as would be expected since the hCFR for RSV-associated ALRI is much lower than that for bacterial ALRI.^10^ However, the above sensitivity analyses suggest that the RSV-ALRI in-hospital mortality estimates might represent an underestimate of the true value.

We estimate that in the first 6 months of life there were 1·4 million (UR 1·2 −1·7) RSV-ALRI hospital admissions, and 27 300 (20 700-36 200) in-hospital deaths, a substantial number of these being in the neonatal period when RSV often presents as apnoea or sepsis. Thus, an effective RSV vaccine for maternal immunisation (with a candidate in phase 3)^17^ or extended half-life monoclonal antibody (candidate to begin phase 3)^18^ could have a substantial impact in this age group. For example, if a future successful maternal immunisation or newborn antibody immunisation strategy could confer 80% protection to infants up to 6 months of age,^19^ then this would have the potential to directly prevent up to 1·1 million hospital admissions and 22 000 in-hospitals deaths globally due to RSV if these immunisations could be achieved with near complete coverage. WHO and key donor agencies have initiated several steps to ensure that a successful RSV vaccine is made available in LMICs without delay.^20^

Our best estimate of overall (combined hospital and community) RSV-ALRI mortality, based on very limited data, is 118 200 (UR 94 600–149 400) deaths in children younger than 5 years and is substantially higher than the GBD 2015 estimates of 36 400 (20 400–61 500) deaths in this age group.^21^ Our data-derived estimates are consistent with RSV being associated with 13–22% of deaths from ALRI in young children in 2015.^2^, ^10^ However, there is substantial uncertainty in this estimate. In our comparative model, we attributed all excess mortality during RSV season to RSV, after adjusting for any overlap with influenza activity. However, the true overlap between RSV and influenza activity could be greater than in our limited data and we did not consider possible seasonal cocirculation of other respiratory pathogens. These could have resulted in an overestimate of overall RSV mortality. Peak pneumococcal mortality is closely linked to (and temporally follows) RSV activity.^22^ A sensitivity analysis extending the RSV season by 1 month and inflating the in-hospital RSV-ALRI mortality accordingly suggests that this could increase RSV-ALRI mortality by about 60% (appendix p 66). Thus, failure to account for this indirect effect on pneumococcal deaths could result in an underestimate of the contribution of RSV to ALRI deaths.

**Figure 4:**
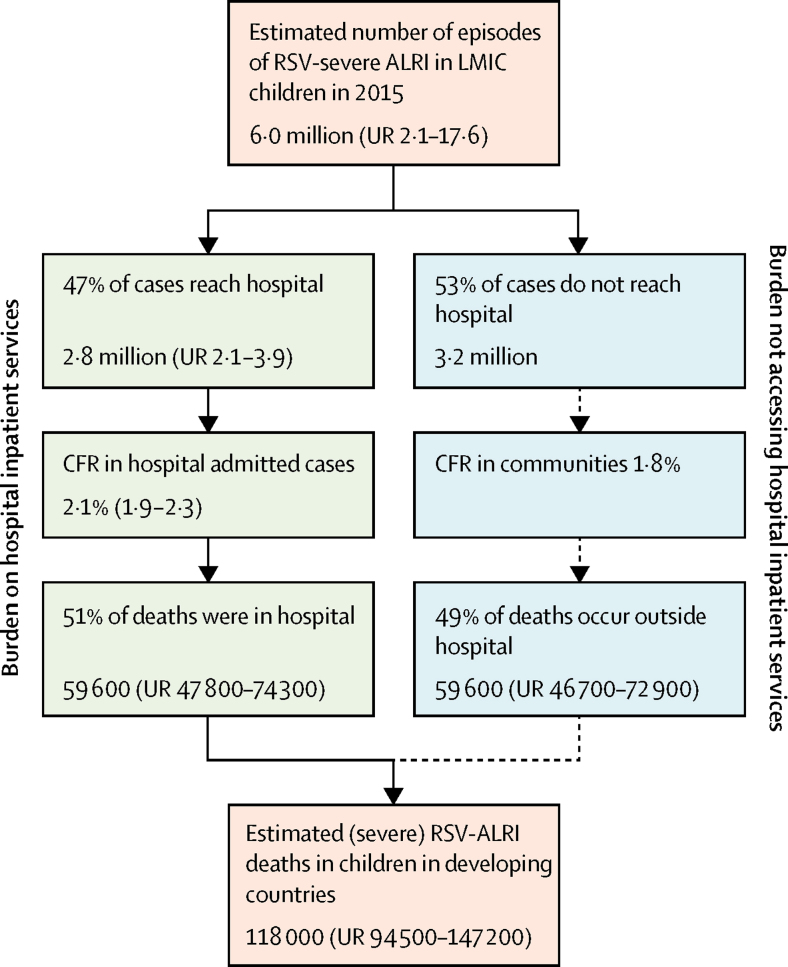
Global burden of RSV-associated severe ALRI including burden on hospital services RSV=respiratory syncytial virus. ALRI=acute lower respiratory infection. Understanding the contribution of RSV to burden on hospital services and the proportion of “severe” cases not accessing hospital care or deaths outside of hospital is relevant for development of health policies to reduce global (RSV-associated) ALRI mortality. The orange boxes show the estimated number of “severe cases” and overall RSV-related deaths in LMICs that are based on relatively limited data. The green boxes show estimated burden on hospital inpatient services that are based on robust data. The blue boxes reflect the inferred (derived) burden estimates for severe cases and deaths that have no access to hospital care.

We have been unable to report estimates of overall RSV-ALRI mortality separately in infants or children younger than 6 months. Further estimates of overall RSV-ALRI mortality from population-based studies with demographic surveillance (which identify child ALRI deaths and conduct RSV and influenza surveillance to define seasonality) could provide more data to allow more robust estimates. In some settings, it might be possible to take respiratory samples soon after death to directly identify RSV-ALRI deaths. Because the current data are consistent with most RSV-ALRI deaths occurring outside of hospital (figure 4), investment in these approaches is warranted to improve estimates of overall RSV-ALRI mortality.

Hypoxaemia is an important indicator of severity and key predictor of ALRI mortality.^23^ About 20% of all children admitted to hospital with RSV-ALRI have hypoxaemia. Our estimates of RSV-ALRI hospital admissions suggests that about half of the severe RSV-ALRI episodes are being admitted to hospital globally and a similar proportion of all RSV deaths occur in hospitals (figure 4). The high proportion of children with severe ALRI who are not admitted to hospital probably reflects limited access to hospital care and conditions that restrict the ability of caregivers to seek hospital care for their children (these studies occurred when WHO recommended hospital admission for all ALRI cases with lower chest wall indrawing).^7^ In Kilifi, Kenya the incidence of RSV-ALRI closest to the hospital was twice the overall incidence (21 *vs* 11 for 1000 infants per year)^24^ with many children referred for hospital care not attending because of cost or cultural factors.^25^ Our estimates of RSV-ALRI hospital admission show the very large referral burden on hospital services in developing countries and this can be expected to further increase as access to and use of health services increases with socioeconomic development.

These updated estimates of 33·1 million (UR 21·6 −50·3) RSV-ALRI episodes resulting in about 3·2 million (UR 2·7 −3·8) million hospital admissions show that RSV in children presents a substantial economic burden on health-care services in view that the direct medical costs associated with hospital care for childhood ALRI has been estimated to range from US$243 (95% CI 154–341) to US$559 (269–887) at secondary and tertiary care facilities, respectively, in LMICs; and $2804 (2001–3683) to $7037 (4286–11 311) at secondary and tertiary care facilities, respectively, in high-income countries.^26^ With an average length of hospital stay for uncomplicated RSV-ALRI in children of about 3 days,^27^, ^28^ this also represents a major challenge for hospital services, requiring substantial investment and seasonal planning both in terms of human resources and provision of relevant medicines and supplies for paediatric care. Simple measures like timely and regular provision of oxygen supplies can substantially decrease RSV-ALRI mortality. The general improvement in diagnosis (particularly availability of pulse oximetry) and improved case management for ALRI is reflected in a decreasing hCFR trend for RSV-ALRI across all age groups and regions (appendix p 94).

A notable difference to our previous estimates is the two-fold increase in the number of severe RSV-ALRI episodes. The current estimate is improved because it is based on many more datapoints and only data from community-based studies employing active case ascertainment (unlike previous estimates based partly on passive case ascertainment studies). However, despite this expanded evidence base, there are still wide uncertainty ranges (appendix p 88). The variation in estimates within countries or regions, and between regions is due to study methodological differences, annual variations in RSV activity (6–75% variation in RSV-ALRI hospital admission rates by year across sites) and variation in RSV epidemiology between study populations. The true uncertainty is wider than that expressed in a standard 95% CI. Data were insufficient to provide regional incidence or hospital admissions rate estimates by RSV subtype.

Several factors affect our estimates, including exact case definitions for (severe) ALRI, case ascertainment method, health-care seeking behaviour of the population, proportion of eligible patients tested for RSV (appendix pp 49–53), geographical location of and environmental conditions at study sites,^29^ sample sizes of included studies and differences in sensitivity and specificity of RSV diagnostic assays. Although we used non-specific case definitions in our analyses, several studies used a more restrictive case definition (eg, including wheeze, fever, crepitations, chest wall indrawing, or chest x-ray confirmation). RSV-ALRI hospital admission rates show a clear gradient across World Bank income regions with lower access to care (including longer distance to hospital) and poorer care seeking behaviour in low-income countries.^24^, ^30^ We have also been unable to account for wide (intracountry) variations in socioeconomic conditions and associated risk factor prevalence in populations residing in middle-income countries.

RSV PCR-based assays were used in 127 of 329 studies; immunofluorescence in 30 studies, direct immunofluorescent antibody test in 74 studies, indirect immunofluorescent antibody test in 18 studies, ELISA in 12 studies, a mixture in 48 studies, and no details were given in 20 studies. Immunofluorescence assays have variable and lower sensitivity (69·4%) compared with PCR.^31^ A sensitivity analysis, including only PCR studies, gave similar hospital admission rate in developing countries (4·6 [95% CI 3·6 −5·7] *vs* 4·9 [4·1–5·8]). We observed a slightly higher incidence rate for community-based studies in developing countries using PCR (59·3 [28·5–123·7] *vs* 50·8 [32·4–79·66]). Causal attribution of pathogens in childhood ALRI is complex due to healthy respiratory carriage of potential pathogens and common presence of multiple agents and is best assessed in case-control studies. Our recent meta-analysis suggests that in about 90% of cases RSV in a nasopharyngeal specimen can be causally attributed to ALRI.^32^

Our revised estimates are based on a substantially larger number of data points from low-income and middle-income countries. However, no data are available from several high burden populations (eg, in the WHO Eastern Mediterranean region and parts of sub-Saharan Africa). Additionally, most studies do not report RSV hospital admission and in-hospital mortality data by narrow age strata in the first year of life, which leads to substantial uncertainty and possible under-estimation of RSV burden in very young children. Unlike in our previous estimate, we have now been able to provide a point estimate with uncertainty ranges for overall RSV-ALRI mortality. However, these are based on very little data and cannot at present support regional mortality estimates. National and regional estimates of burden on health-care systems, long-term sequelae and mortality are required to inform policy for introduction of RSV vaccines and also to assess the effect of these vaccines on morbidity and mortality in young children. Therefore, further research investment to identify RSV-ALRI mortality (in community and in hospitals) in low-income and middle-income countries is warranted.
