# Supplementary material for: Global, regional, and national disease burden estimates of acute lower respiratory infections due to respiratory syncytial virus in young children in 2015: a systematic review and modelling study
Source: Lancet. 2017 Sep 2;390(10098):946–58. doi: 10.1016/S0140-6736(17)30938-8 (PMC5592248; doi:10.1016/S0140-6736(17)30938-8)
Supplement: Supplementary appendix [file mmc1.pdf]

THE LANCET

Supplementary appendix

This appendix formed part of the original submission. We post it as supplied by the authors.

Supplement to: Shi T, McAllister D A, L O'Brien K. Global, regional, and national disease burden estimates of acute lower respiratory infections due to respiratory syncytial virus in young children in 2015: a systematic review and modelling study. *Lancet* 2017; published online July 6. http://dx.doi.org/10.1016/S0140-6736(17)30938-8.

Supplementary material for "Global, regional and national disease burden estimates of acute lower respiratory infections due to respiratory syncytial virus in young children in 2015:

A systematic review and modelling study"

Glossary

1. RSV-associated ALRI: cough or difficulty breathing with increased respiratory rate for age (cut-offs same as in WHO Integrated Management of Childhood Illnesses [IMCI] case definition) and laboratory confirmed RSV.
2. RSV-associated severe ALRI: children aged 2-59 months- cough or difficulty in breathing with chest wall indrawing and laboratory confirmed RSV; children aged <2months- increased RR (>60 breaths/ min) OR chest wall indrawing and laboratory confirmed RSV
3. RSV-associated very severe ALRI: cough or difficulty in breathing with one danger sign (cyanosis, difficulty in breastfeeding or drinking, vomiting everything, convulsions, lethargy, or unconsciousness, head nodding) and
4. Hospitalised RSV-associated ALRI: all children with physician confirmed diagnosis of ALRI (pneumonia or bronchiolitis) that are hospitalised or recommended hospitalisation and laboratory confirmed RSV
5. Hospitalised RSV-associated severe ALRI: Hospitalised ALRI cases with hypoxemia (as defined below) and laboratory confirmed RSV
6. Hospitalised RSV-associated very severe ALRI: hospitalised ALRI with one danger sign (cyanosis, difficulty in breastfeeding or drinking, vomiting everything, convulsions, lethargy, or unconsciousness, head nodding) OR proxies for very severe disease – mechanical ventilation OR ICU admission.
7. Hypoxemia: SpO_2 <90% (at altitude ≤2500 m) in children aged 1-59 months and <88% for neonates (at sea level). At altitude above 2500m, SpO_2 <87% in children aged 1-59 months and <85% for neonates would classify as hypoxemia.
8. LMIC: low income countries, lower middle income countries and upper middle income countries as per World Bank Classification.
9. Neonates: children aged 0-27 days.
10. Inflation factor: the ratio between RSV-associated ALRI mortality in community and RSV-associated ALRI deaths in hospital.

Search Strategy

Medline

1. exp Respiratory Syncytial Viruses/ or exp Respiratory Syncytial Virus, Human/ or exp Respiratory Syncytial Virus Infections/ or RSV.mp.

2. respiratory syncytial virus*.mp.

3. pneumonia.mp. or exp Pneumonia/ or exp Pneumonia, Viral/

4. bronchiolitis.mp. or exp Bronchiolitis/ or exp Bronchiolitis, Viral/

5. exp Respiratory Tract Infections/ or respiratory infection*.mp.

6. exp Respiratory Tract Diseases/ or respiratory disease*.mp.

7. incidence.mp. or exp Incidence/

8. prevalence.mp. or exp Prevalence/

9. exp Child Mortality/ or exp Infant Mortality/ or mortality.mp. or exp Hospital Mortality/ or exp Mortality/

10. death*.mp. or exp Death/ or exp "Cause of Death"/

11. morbidity.mp. or exp Morbidity/

12. burden.mp.

13. epidemiology.mp. or exp Epidemiology/

14. 1 or 2

15. 3 or 4 or 5 or 6

16. 7 or 8 or 9 or 10 or 11 or 12 or 13

17. 15 or 16

18. 14 and 17

19. limit 18 to (yr="1995 -Current" and ("all infant (birth to 23 months)" or "newborn infant (birth to 1 month)" or "infant (1 to 23 months)" or "preschool child (2 to 5 years)") and (female or humans or male))

EMBASE

1. RSV.mp. or exp Respiratory syncytial pneumovirus/ or exp respiratory syncytial virus infection/

2. respiratory syncytial virus*.mp.

3. exp community acquired pneumonia/ or exp pneumonia/ or exp virus pneumonia/ or exp infectious pneumonia/ or pneumonia.mp.

4. bronchiolitis.mp. or exp bronchiolitis/ or exp viral bronchiolitis/

5. exp respiratory tract infection/ or exp lower respiratory tract infection/ or respiratory infection*.mp.

6. exp respiratory tract disease/ or respiratory disease*.mp.

7. exp incidence/ or incidence.mp.

8. prevalence.mp. or exp prevalence/

9. exp newborn mortality/ or exp mortality/ or exp childhood mortality/ or mortality.mp. or exp infant mortality/

10. exp death/ or death*.mp. or exp "cause of death"/ or exp child death/

11. morbidity.mp. or exp morbidity/ or exp newborn morbidity/

12. burden.mp.

13. exp epidemiology/ or epidemiology.mp.

14. 1 or 2

15. 3 or 4 or 5 or 6

16. 7 or 8 or 9 or 10 or 11 or 12 or 13

17. 15 or 16

18. 14 and 17

19. limit 18 to (yr="1995 -Current" and (infant or preschool child <1 to 6 years>))

Global Health

1. RSV.mp.

2. exp human respiratory syncytial virus/

3. respiratory syncytial virus*.mp.

4. exp community acquired pneumonia/ or pneumonia*.mp. or exp pneumonia/

5. bronchiolitis.mp. or exp bronchiolitis/

6. respiratory infection*.mp.

7. exp respiratory diseases/

8. respiratory disease*.mp.

9. incidence.mp. or exp disease incidence/ or exp incidence/

10. prevalence*.mp. or exp disease prevalence/

11. mortality.mp. or exp infant mortality/ or exp neonatal mortality/ or exp mortality/

12. death*.mp. or exp death/ or exp "causes of death"/

13. morbidity.mp. or exp morbidity/

14. exp epidemiology/ or epidemiology.mp.

15. burden.mp.

16. 1 or 2 or 3

17. 4 or 5 or 6 or 7 or 8

18. 9 or 10 or 11 or 12 or 13 or 14 or 15

19. 17 or 18

20. 16 and 19

21. limit 20 to yr="1995 -Current"

CINAHL

S1= (MH “respiratory syncytial virus infections”) OR (MH “respiratory syncytial viruses”) OR “respiratory syncytial virus”

S2= "RSV"

S3= (MH "pneumonia+") OR "pneumonia" OR (MH "pneumonia, viral") OR (MH "community-acquired pneumonia")

S4= (MH "bronchiolitis+") OR "bronchiolitis"

S5= (MH "respiratory tract infections+") OR "respiratory infection"

S6= (MH "respiratory tract diseases+") OR "respiratory disease"

S7= (MH "incidence") OR "incidence"

S8= (MH "prevalence") OR "prevalence"

S9= (MH "mortality+") OR "mortality" OR (MH "infant mortality") OR (MH "child mortality") OR (MH "hospital mortality")

S10= (MH "death+") OR "death" OR (MH "cause of death") OR (MH "infant death+")

S11= (MH "morbidity+") OR "morbidity"

S12= "burden"

S13= (MH "epidemiology+") OR "epidemiology"

S14= S1 OR S2

S15= S3 OR S4 OR S5 OR S6

S16= S7 OR S8 OR S9 OR S10 OR S11 OR S12 OR S13

S17= S15 OR S16

S18= S14 AND S17

Limiters: Published date: 19950101 – 20150804; Age groups: infant, newborn: birth-1 month, infant: 1-23 months, child, preschool: 2-5 years

Web of Science

Topic= (respiratory syncytial virus) AND Topic= (epidemiology) AND Topic= (children)

From 1995 to 2015

WHOLIS (KMS)

Respiratory syncytial virus in All Indexes

LILACS (AMRO/PAHO)

Respiratory syncytial virus in All Indexes

IndMed

RSV in anywhere

SIGLE

Respiratory syncytial virus

CNKI

Topic: respiratory infection or pneumonia (vague)

Or Topic: bronchiolitis (vague)

And Topic: respiratory syncytial virus (vague)

And Topic: children (vague)

Publication time: 1st Jan 1995 – 30th Aug 2015

Wanfang Data

All (vague): respiratory infection or

All (vague): pneumonia or

All (vague): bronchiolitis and

All (vague): respiratory syncytial virus and

All (vague): children

Time: 1995 – 2015

ChongQing VIP

Title/key word: respiratory tract infection

Or Title/key word: pneumonia

Or Title/key word: bronchiolitis

And Title/key word: respiratory syncytial virus

And Title/key word: children

Time: 1995 – 2015

Imputation for missing data

Only 40 studies (12 published[1-11] and 28 unpublished[U3-5, U8, U11, U15-24, U26-29, U32, U34-37, 39-40, U42, U45]) reported disease incidence / hospitalisation rate by age group for the full age range (0-59m group); we imputed data for the remaining studies (supplementary tables 2,3 and 5)

For community-based studies reporting incidence rate, relative to an incidence of 1.0 for RSV-associated ALRI in the 0-11 months age group, an incidence rate ratio of 1.01 was calculated for children aged 0-5 months (based on 9 studies), 0.90 for children aged 0-23 months (8 studies), 0.84 for children aged 0-35m (6 studies) and 0.57 for those aged 0-59 months (6 studies), by taking the median of the incidence rate ratios of studies reporting incidence for these age ranges[12-14]. For RSV-associated severe ALRI, an incidence rate ratio of 1.26 was calculated for children aged 0-5 months (based on 7 studies), whereas for those younger than 2 years, 3 years and 5 years, the ratios were 0.64 (7 studies), 0.56 (4 studies) and 0.34 (4 studies). For hospital-based studies reporting hospitalisation rate, relative to children younger than 1 year, the median hospitalisation rate ratio for children aged 0-5 months was 1.32 (based on 27 studies), for those younger than 2 years, 3 years and 5 years, the median rate ratios were estimated as 0.63 (38 studies), 0.44 (23 studies) and 0.27 (35 studies) respectively.

Sensitivity analysis

We conducted several sensitivity analyses by excluding imputed data and found that in general the meta-estimates based on including the full dataset (including imputed data) had slightly lower or higher point estimates and narrower 95% CI. However, inclusion of imputed data increased the point estimates for the incidence rate in 0-59 month old children by over 40%. However, this increase is not statistically significant as the uncertainty range of both estimates overlap each other. Secondly, the large increase in the point estimate for incidence rate in this case is driven by data from two recent high quality studies (Drakenstein birth cohort study in South Africa [Zar, unpublished] and RESPIRA-PERU study in rural highlands of Peru [Wu et al, 2015]) as can be evinced by the incidence rate after excluding these 2 studies from the sensitivity analysis using imputed data - 40.4 (25.0 – 65.2) per 1000 children below 5 years per year. Both studies are of high quality but in children below 3 years and report a high(er) incidence rate in infants and in the second year of life.

Comparison of incidence rate meta-estimates after excluding imputed data

Region	0-11m				0-59m			
	Excluding imputed data		Including imputed data		Excluding imputed data		Including imputed data	
	No.	Incidence rate	No.	Incidence rate	No.	Incidence rate	No.	Incidence rate
**Developing**	11	99.3 (62.6-157.3)	14	88.3 (58.6-133)	6	35.5 (17.2-73.1)	14	50.8 (32.4-79.7)

Comparison of hospitalisation rate meta-estimates after excluding imputed data

Region	0-11m				0-59m			
	Excluding imputed data		Including imputed data		Excluding imputed data		Including imputed data	
	No.	Hospitalisation rate	No.	Hospitalisation rate	No.	Hospitalisation rate	No.	Hospitalisation rate
**Low income**	5	5.6 (1.7-18.1)	5	5.6 (1.7-18.1)	4	1.3 (0.3-5.1)	5	1.7 (0.6-5.1)
**Lower middle income**	15	18.4 (13.7-24.8)	17	18.2 (13.9-23.8)	12	4.9 (3.5-6.9)	17	5.1 (3.7-6.9)

Region	0-11m				0-59m			
	Excluding imputed data		Including imputed data		Excluding imputed data		Including imputed data	
	No.	Hospitalisation rate	No.	Hospitalisation rate	No.	Hospitalisation rate	No.	Hospitalisation rate
**Upper middle income**	12	19.2 (12.8-28.7)	15	18.5 (13.3-25.8)	12	5.3 (4.2-6.7)	15	6.2 (5.0-7.8)
**High income**	21	20.8 (17.4-24.9)	31	19.6 (16.9-22.8)	14	5.3 (4.0-7.0)	31	5.5 (4.7-6.3)
**Developing**	36	16.2 (13.0-20.1)	43	16.0 (13.2-19.4)	33	4.3 (3.5-5.1)	43	4.9 (4.1-5.8)
**Industrialised**	17	20.9 (17.1-25.4)	25	20.2 (17.2-23.8)	9	5.8 (4.1-8.2)	25	5.5 (4.7-6.5)

Supplementary table 1: List of unpublished studies from RSV GEN collaborators contributing to the analysis

	Location (reference)	Published reference	Remarks
**U1**	Kilifi birth cohort, Kenya (Nokes and colleagues)	Clin Infect Dis 2008; 46:50-7	report both community-based rate and hospital-based rate; data re-analysed using common case definitions
**U2**	San Marcos, Guatemala (Bruce and colleagues)	Lancet 2011; 378(9804): 1717-26	data re-analysed using common case definitions
**U3**	Dhaka, Bangladesh (Brooks and colleagues)	Pediatr Infect Dis J 2010; 29(3): 216-21	RSV was tested for the first 4 years of study; 15 ALRI deaths without information about virus
**U4**	Ballabhgarh, India (Krishnan and colleagues)	BMC Infect Dis 2015; 15: 462	data re-analysed using common case definitions
**U5**	Bandung, Indonesia (Simoes and colleagues)	Pediatr Infect Dis J 2011; 30(9): 778-84	data re-analysed using common case definitions
**U6**	Managua, Nicaragua (Gordon and colleagues)	Pediatr Infect Dis J 2016; 35(2): 152-6	data re-analysed using common case definitions
**U7**	Matiari, Pakistan (Ali and colleagues)	PLoS One 2013; 8(9)	data re-analysed using common case definitions
**U8**	Gilgit, Pakistan (Rasmussen and colleagues)	no	data are not yet published
**U9**	Puerto Maldonado, Peru (Romero and colleagues)	no	only one very severe case in 12-23m
**U10**	Paarl, South Africa (Zar and colleagues)	i. Thorax 2015; 70(6): 592-4<br>ii. Lancet Glob Health 2015; 3(2): e95-e103	report both community-based rate and hospital-based rate; data re-analysed using common case definitions
**U11**	Nashville, Tennessee, USA (Wright and colleagues)	no	data are not yet published; report rate in outpatients in industrialised countries
**U12**	CEMIC, Buenos Aires, Argentina (Echavarria and colleagues)	i. Arch Argent Pediatr 2011;109(4):296-304<br>ii. Pediatr Infect Dis J 2013; 32(3): e105-e110<br>iii. Medicina 2012; 72(1): 28-32	another paper is in preparation; data re-analysed using common case definitions
**U13**	Buenos Aires, Argentina (Polack and colleagues)	Am J Respir Crit Care Med 2013; 187(9): 983-90	data re-analysed using common case definitions
**U14**	Kishoregonj, Bogra, Comilla and Barisal, Bangladesh (Homaira and colleagues)	PLoS One 2016; 11(2): e0147982	data re-analysed using common case definitions
**U15**	Belo Horizonte, Brazil (Oliveira and colleagues)	no	data are not yet published
**U16**	Concepcion, Chile (Fasce and colleagues)	no	data are not yet published
**U17**	Iquique, Chile (Fasce and colleagues)	no	data are not yet published
**U18**	Jingzhou, China (Yu and colleagues)	J Infect Dis 2013; 208 Suppl 3: S184-8	data re-analysed using common case definitions
**U19**	Santa Ana, El Salvador (Clara and colleagues)	i. Bull World Health Organ 2012; 90(10): 756-63<br>ii. Influenza Other Respir Viruses 2016	data re-analysed using common case definitions

	Location (reference)	Published reference	Remarks
**U20**	Gambia Western Region, Gambia (Howie and colleagues)	Clin Infect Dis 2014; 59(5): 682-5	data re-analysed using common case definitions
**U21**	Santa Rosa, Guatemala (McCracken and colleagues)	i. Infect Dis 2013; 208 Suppl 3: S197-206<br>ii. Pediatr Infect Dis J 2013; 32(6): 629-35	data re-analysed using common case definitions
**U22**	Quetzaltenango, Guatemala (McCracken and colleagues)	i. Infect Dis 2013; 208 Suppl 3: S197-206<br>ii. Pediatr Infect Dis J 2013; 32(6): 629-35	data re-analysed using common case definitions
**U23**	Ballabhgarh, India (Broor and colleagues)	J Infect 2015; 70(2): 160-70	data re-analysed using common case definitions
**U24**	Pune, India (Chadha and colleagues)	J Infect 2015; 70(2): 160-70	data re-analysed using common case definitions
**U25**	Lombok, Indonesia (Gessner and colleagues)	Epidemiol Infect 2008; 136(10): 1319-27	data re-analysed using common case definitions
**U26**	Kilifi hospital study, Kenya (Nokes and colleagues)	JAMA 2010; 303(20): 2051-7	data re-analysed using common case definitions
**U27**	Bondo district, Kenya (Feikin and colleagues)	J Med Virol 2013; 85(5): 924-32	data re-analysed using common case definitions
**U28**	Manhiça, Mozambique (Bassat and colleagues)	Pediatr Infect Dis J 2011; 30(1): 39-44	data re-analysed using common case definitions
**U29**	Manhiça, Mozambique (Bassat and colleagues)	Pediatr Infect Dis J 2011; 30(12): 1032-6	data re-analysed using common case definitions
**U30**	Utrecht, Netherlands (Bont and colleagues)	i. Pediatr Infect Dis J 2014; 33(1): 19-23<br>ii. Pediatrics 2011; 127(1): 35-41	healthy term infants; data re-analysed using common case definitions
**U31**	41 sites in Netherlands (Bont and colleagues)	N Engl J Med 2013; 368(19): 1791-9	late preterm infants; data re-analysed using common case definitions
**U32**	David City, Panama (Jara and colleagues)	no	data are not yet published
**U33**	Tagbilaran and 6 rural sites, Philippines (Lucero and colleagues)	i. Pediatr Infect Dis J 2009; 28(6): 455-62<br>ii. J Clin Microbiol 2013; 51(3): 945-53	data re-analysed using common case definitions
**U34**	Gauteng province, South Africa (Cohen and colleagues)	i. J Infect Dis 2013; 208 Suppl 3: S217-26<br>ii. J Infect Dis 2012; 206 Suppl 1: S159-65	data re-analysed using common case definitions
**U35**	KwaZulu-Natal province, South Africa (Cohen and colleagues)	i. J Infect Dis 2013; 208 Suppl 3: S217-26<br>ii. J Infect Dis 2012; 206 Suppl 1: S159-65	data re-analysed using common case definitions
**U36**	Klerksdorp site, South Africa (Cohen and colleagues)	i. J Infect Dis 2013; 208 Suppl 3: S217-26<br>ii. J Infect Dis 2012; 206 Suppl 1: S159-65	data re-analysed using common case definitions
**U37**	Soweto, South Africa (Madhi and colleagues)	J Clin Virol 2006; 36(3): 215-21	data re-analysed using common case definitions
**U38**	Maela Camp, Tak Province, Thailand (Turner and colleagues)	i. PLoS ONE 2012; 7(11): e50100<br>ii. BMC Infect Dis 2013; 13(1)	data re-analysed using common case definitions
**U39**	Sa Kaeo and Nakhon Phanom, Thailand (Thamthitiwat and colleagues)	i. Pediatr Infect Dis J 2014; 33(2): e45-e52<br>ii. J Infect Dis 2013; 208 Suppl 3:	data re-analysed using common case definitions

	Location (reference)	Published reference	Remarks
		S238-45	
**U40**	Tone district, Togo (Gessner and colleagues)	no	data are not yet published
**U41**	Alaska, USA (Singleton and colleagues)	i. Pediatr Infect Dis J 2007; 26(11(Supplement): S46-S50<br>ii. J Med Virol 2010; 82(7): 1282-90	report very severe cases from 1994 to 2007; hypoxemia data from Oct 2005 to Sep 2007; data re-analysed using common case definitions
**U42**	Colorado, USA (Simoes and colleagues)	no	ICD-9 codes for hypoxemia
**U43**	Navajo and WMA, USA - MEDI (O'Brien and colleagues)	submitted to journal	data re-analysed using common case definitions
**U44**	Navajo and WMA, USA - EPI (O'Brien and colleagues)	Pediatrics 2002; 110(2 Pt 1): e20	data re-analysed using common case definitions
**U45**	Nha Trang, Vietnam (Yoshida and colleagues)	i. Pediatr Infect Dis J 2010; 29(1): 75-7<br>ii. Eur Respir J 2013; 42(2): 461-9	subtypes data from 2010 to 2012; another paper is in preparation; data re-analysed using common case definitions
**U46**	Buenos Aires, Argentina (Echavarria and colleagues)	i. Arch Argent Pediatr 2011;109(4):296-304<br>ii. Pediatr Infect Dis J 2013; 32(3): e105-e110<br>iii. Medicina 2012; 72(1): 28-32	another paper is in preparation; data re-analysed using common case definitions
**U47**	Buenos Aires, Argentina (Gentile and colleagues)	Arch Argent Pediatr 2014; 112(5): 397-404	data re-analysed using common case definitions
**U48**	Takeo and Kampong Cham, Cambodia (Goyet and colleagues)	Pediatr Infect Dis J 2013; 32(1): e8-13	data re-analysed using common case definitions
**U49**	Chillan, Chile (Fasce and colleagues)	no	data are not yet published
**U50**	Osorno, Chile (Fasce and colleagues)	no	data are not yet published
**U51**	Punta Arenas, Chile (Fasce and colleagues)	no	data are not yet published
**U52**	Santiago, Chile (Fasce and colleagues)	no	data are not yet published
**U53**	Valparaiso, Chile (Fasce and colleagues)	no	data are not yet published
**U54**	China (Yu and colleagues)	PLoS One 2014; 9(6): e99419	data re-analysed using common case definitions
**U55**	Beijing, China (GABRIEL)	no	data are not yet published
**U56**	Ulaanbaatar, Mongolia (GABRIEL)	no	data are not yet published
**U57**	Phnom Penh, Cambodia (GABRIEL)	no	data are not yet published
**U58**	Lucknow, India (GABRIEL)	no	data are not yet published
**U59**	Antananarivo, Madagascar (GABRIEL)	no	data are not yet published
**U60**	Asuncion, Paraguay (GABRIEL)	no	data are not yet published
**U61**	Bamako, Mali (GABRIEL)	no	data are not yet published
**U62**	Berlin, Germany (Rath and colleagues)	i. iagn Microbiol Infect Dis 2015; 81(3): 171-6<br>ii. J Clin Microbiol 2009; 47(6):	data re-analysed using common case definitions

	Location (reference)	Published reference	Remarks
		1800-10	
**U63**	Amman, Jordan (Khuri and colleagues)	Vaccine 2015; 33(47): 6479-87	data re-analysed using common case definitions
**U64**	Lwak, Kenya (Montgomery and colleagues)	J Infect Dis 2013; 208 Suppl 3: S207-16	data re-analysed using common case definitions
**U65**	Rabat, Morocco (Bassat and colleagues)	i. J Trop Pediatr 2014; 60(4): 270-8<br>ii. Epidemiol Infect 2016; 144(3): 516-26	data re-analysed using common case definitions
**U66**	Kathmandu, Nepal (Basnet and colleagues)	Pediatr Infect Dis J 2011; 30(12): 1032-6	data re-analysed using common case definitions
**U67**	Karachi, Pakistan (Ali and colleagues)	PLoS One 2013; 8(9)	data re-analysed using common case definitions
**U68**	Basse, Gambia (PERCH)	no	data are not yet published
**U69**	Kilifi, Kenya (PERCH)	no	data are not yet published
**U70**	Bamako, Mali (PERCH)	no	data are not yet published
**U71**	Nakhon Phanom and Sa Kaeo, Thailand (PERCH)	no	data are not yet published
**U72**	Lusaka, Zambia (PERCH)	no	data are not yet published
**U73**	Dhaka, Bangladesh (PERCH)	no	data are not yet published
**U74**	Matlab, Bangladesh (PERCH)	no	data are not yet published
**U75**	Tacloban, Philippines (Lupisan and colleagues)	BMC Infect Dis 2012; 12: 267	data re-analysed using common case definitions
**U76**	Mpumalanga, South Africa (Cohen and colleagues)	i. J Infect Dis 2013; 208 Suppl 3: S217-26<br>ii. J Infect Dis 2012; 206 Suppl 1: S159-65	data re-analysed using common case definitions

Supplementary table 2: Description of all studies reporting incidence of RSV-associated ALRI in children younger than 5 years

Location (reference)	Case definition (codes)*	Denominator source	Specimen and diagnostic test*	Incidence of RSV associated ALRI (per 1000 children per year)					
				0-5m	6-11m	0-11m	12-23m	24-59m	0-59m
San Marcos, Peru (rural, Mar 2009 - Sep 2011)[15]	I	Defined population base	nasal swab; RT-PCR	343.8	338.1	340.8	304.3		(194.3)
Dhaka, Bangladesh (urban, Apr 2009 - Mar 2011)[16]	I	Defined population base	NPW; RT-PCR	150.0	110.0	(148.5)†	120.0		(84.7)
Kilifi birth cohort, Kenya (rural, Jan 2002 - May 2005) (Nokes and colleagues)	I, O2+/-	Defined population base	NPA, nasal wash; DFA	147.0	63.0	105.1	71.0		(59.9)
Mirzapur, Bangladesh (rural, Oct 1993 - Aug 1996)[17]	I	Defined population base	NPA; ELISA	(46.8)		(46.3)			(26.4)
Ballabhgarh, India (rural, Oct 2001 - Mar 2005)[18]	I	Defined population base	NPA; DFA	60.2	18.0	38.0	52.0		(21.7)
Ibadan, Nigeria (rural, Jun 1999 - May 2001)[1]	I	Defined population base	NPA, NPW; ELISA	(117.2)		116.0			94.0
San Marcos, Guatemala (rural, Dec 2002 - Dec 2004) (Bruce and colleagues)	I, II, DS+/-	Defined population base	NPA; DFA	107.7	172.4	147.5	124.5		(84.1)
Rio de Janeiro, Brazil (rural, Jan 1987 - Dec 1989)[19]	II, T+/-, W+/-	Defined population base	NPA; IFA, culture	(24.8)		(24.6)			14.0
Dhaka, Bangladesh (urban, Jan 2005 - Feb 2008) (Brooks and colleagues)	I, W+/C+, DS+/-	Defined population base	NPW, serum; serology, RT-PCR	28.3	36.7	32.2	21.8	9.5	17.5
Ballabhgarh, India (rural, Aug 2012 - Aug 2013) (Krishnan and colleagues)	I, DS+/-	Defined population base	NPS; RT-PCR	97.3	76.9	85.5	54.6	10.1	33.4
Bandung, Indonesia (periurban and rural, 1st Feb 1999 - 26th Apr 2001) (Simoes and colleagues)	I, DS+/-	Defined population base	nasal wash; RT-PCR	26.6	141.5	87.9	97.3	21.1	65.4
Managua, Nicaragua (urban, Sep 2011 - Sep 2012) (Gordon and colleagues)	I, DS+/-	Defined population base	nasal and throat swab; RT-PCR	(61.4)		60.8			(34.7)

* NPA=nasopharyngeal aspirate. NPS=nasopharyngeal swab. NPW=nasopharyngeal wash. RT-PCR=reverse transcriptase polymerase chain reaction. IFA=indirect immunofluorescent antibody test. DFA=direct immunofluorescent antibody test. ELISA=enzyme-linked immunosorbent assay. I=WHO definition applied by a health worker. II=physician's assessment. T+/-=tachypnea part of case definition or not. W+/-=wheeze part of case definition or not. DS+/-=danger signs part of case definition or not. O2+/-=hypoxemia part of case definition or not. C+/-=crepitation part of case definition or not.

† Data in parentheses are imputed

Location (reference)	Case definition (codes)*	Denominator source	Specimen and diagnostic test*	Incidence of RSV associated ALRI (per 1000 children per year)					
				0-5m	6-11m	0-11m	12-23m	24-59m	0-59m
Gilgit, Pakistan (rural, 1st Apr 2012 - 31st Mar 2014) (Rasmussen and colleagues)	I, DS+/-	Defined population base	NPS; RT-PCR	30.8	101.9	62.5	58.6	15.9	35.9
Paarl, South Africa (periurban, Mar 2012 - Dec 2014) (Zar and colleagues)	I, DS+/-	Defined population base	NPS; RT-PCR	506.7	141.8	331.0	78.6		(188.7)
Nashville, Tennessee, USA (urban, Jan 1973 - Dec 1993) (Wright and colleagues) ‡	II	Defined population base	NPW; culture	(46.3)		45.8			24.0
Multicentric, Germany (urban, Nov 1999 - Oct 2001) †20	II	Census-derived estimate	NPS; PCR	(92.6)		(91.7)			(52.3)

‡ Outpatient clinic based

Supplementary table 3: Description of all studies reporting incidence of RSV-associated severe ALRI in children younger than 5 years

Location (reference)	Case definition (codes)*	Denominator source	Specimen and diagnostic test*	Incidence of RSV associated severe ALRI (per 1000 children per year)					
				0-5m	6-11m	0-11m	12-23m	24-59m	0-59m
Kilifi birth cohort, Kenya (rural, Jan 2002 - May 2005) (Nokes and colleagues)	I, O2+/-	Defined population base	NPA, nasal wash; DFA	86.0	47.0	68.5	17.0		(23.3)†
Ballabhgarh, India (rural, Oct 2001 - Mar 2005)[18]	I	Defined population base	NPA; DFA	20.1	9.0	14.3	6.5		(4.8)
Dhaka, Bangladesh (urban, Jan 2005 - Feb 2008) (Brooks and colleagues)	I, W+/C+, DS+/-	Defined population base	NPW, serum; serology, RT-PCR	5.1	1.4	3.4	0.5		1.0
Ballabhgarh, India (rural, Aug 2012 - Aug 2013) (Krishnan and colleagues)	I, DS+/-	Defined population base	NPS; RT-PCR	53.1	51.3	52.0	32.1	2.3	17.7
Bandung, Indonesia (periurban and rural, 1st Feb 1999 - 26th Apr 2001) (Simoes and colleagues)	I, DS+/-	Defined population base	nasal wash; RT-PCR	(24.1)	35.8	19.1	13.0	3.1	11.2
Managua, Nicaragua (urban, Sep 2011 - Sep 2012) (Gordon and colleagues)	I, DS+/-	Defined population base	nasal and throat swab; RT-PCR	(28.9)		22.9			(7.8)
Matiari, Pakistan (rural, Oct 2011 - Jun 2014) (Ali and colleagues)	I, F+/-	Census-derived estimate	NPS; RT-PCR	19.0	22.2	20.6	3.1		(7.0)
Paarl, South Africa (periurban, Mar 2012 - Dec 2014) (Zar and colleagues)	I, DS+/-	Defined population base	NPS; RT-PCR	406.7	82.1	253.5	36.9		(86.2)
Nashville, Tennessee, USA (urban, Jan 1973 - Dec 1993) (Wright and colleagues)‡	II	Defined population base	NPW; culture	(3.2)		9.5			3.0

* NPA=nasopharyngeal aspirate. NPS=nasopharyngeal swab. NPW=nasopharyngeal wash. RT-PCR=reverse transcriptase polymerase chain reaction. DFA=direct immunofluorescent antibody test. I=WHO definition applied by a health worker. II=physician's assessment. W+/-=wheeze part of case definition or not. DS+/-=danger signs part of case definition or not. F+/-=fever part of case definition or not. O2+/-=hypoxemia part of case definition or not. C+/-=crepitation part of case definition or not

† Data in parentheses are imputed

‡ Outpatient clinic based

Supplementary table 4: Description of all studies reporting incidence of RSV-associated very severe ALRI in children younger than 5 years

Location (reference)	Case definition (codes)*	Denominator source	Specimen and diagnostic test*	Incidence rate of RSV associated very severe ALRI (per 1000 children per year)					
				0-5m	6-11m	0-11m	12-23m	24-59m	0-59m
Puerto Maldonado, Peru (periurban, Jan - Dec 2013) (Romero and colleagues)	I, W+/C+, DS+/-	Defined population base	NPW, serum; serology, RT-PCR				18.2		4.4
Paarl, South Africa (periurban, Mar 2012 - Dec 2014) (Zar and colleagues)	I, DS+/-	Defined population base	NPS; RT-PCR	26.7	14.9	21.1	2.5		
Dhaka, Bangladesh (urban, Jan 2005 - Feb 2008) (Brooks and colleagues)	I, DS+/-	Defined population base	OPS; RT-PCR	1.1	0.5	0.8			0.2
Ballabhgarh, India (rural, Aug 2012 - Aug 2013) (Krishnan and colleagues)	I, DS+/-	Defined population base	NPS; RT-PCR	17.7	6.4	11.1	6.4		3.4

* NPS=nasopharyngeal swab. NPW=nasopharyngeal wash. OPS=oropharyngeal swab. RT-PCR=reverse transcriptase polymerase chain reaction. I=WHO definition applied by a health worker. W+/-=wheeze part of case definition or not. DS+/-=danger signs part of case definition or not. C+/-=crepitation part of case definition or not

Supplementary table 5: Description of all studies reporting hospitalisation rate of RSV-associated ALRI in children younger than 5 years

Location (reference)	Case definition (codes)*	Denominator source	Specimen and diagnostic test*	Hospitalisation rate of RSV associated ALRI (per 1000 children per year)					
				0-5m	6-11m	0-11m	12-23m	24-59m	0-59m
Alice Springs, Australia (urban, 1st Jan 2000 - 31st Dec 2004)[21]	II	Census-derived estimate	NPA; DFA	(42.7)†		(32.4)			(8.7)
Suzhou, China (urban, Jan 2007 - Dec 2008)[22]	II	Census-derived estimate	nasal aspirate; DFA	(14.1)		(10.7)			2.9
Hong Kong, China (urban, 2003 - 2006)[3]	II, F+	Census-derived estimate	NPA; DFA, culture	26.8	10.4	18.7	10.1	4.4	13.0
Sa Kaeo and Nakhom Phanom, Thailand (rural, 1st Jan 2004 - 31st Dec 2007)[4]	II, XR	Census-derived estimate	NPS, serum; RT-PCR, IFA	(14.3)		10.9			5.4
Damanhour, Egypt (urban and rural, Jun 2009 - Jun 2012)[10]	II	Census-derived estimate	NPS, OPS; RT-PCR	(23.0)		17.5			2.4
Lørenskog, Norway (periurban, 1993 - 2000)[23]	II	Census-derived estimate	NPA; ELISA	(28.6)		21.7	6.8		(5.9)
Valencia, Spain (urban, 2001 - 2002)[24]	II	Census-derived estimate	NA‡	(37.2)		(28.2)	9.8		(7.6)
Gipuzkoa, Spain (urban, Jul 2004 - Jun 2007)[25]	II	Census-derived estimate	NPA; RT-PCR	39.4	10.8	25.1	3.0		(6.8)
Bursa, Turkey (urban, 1st Mar 2010 - 28th Feb 2011)[26]	II	Census-derived estimate	nasal swab; IF	(16.1)		(12.2)			(3.3)
USA (urban and rural, 1997 - 2006)[27]	II	Census-derived estimate	NA	(32.8)	13.4	(24.8)	5.0	0.8	6.7
Dadaab and Kakuma, Kenya (rural, Sep 2007 - Aug 2010)[2]	III, T+/-, CWI+/-, O2+/-	Census-derived estimate	NPS, OPS; RT-PCR	(38.3)		29.0			9.3

* NPA=nasopharyngeal aspirate. NPS=nasopharyngeal swab. NPW=nasopharyngeal wash. OPS=oropharyngeal swab. RT-PCR=reverse transcriptase polymerase chain reaction. IFA=indirect immunofluorescent antibody test. DFA=direct immunofluorescent antibody test. ELISA=enzyme-linked immunosorbent assay. IF=immunofluorescence. II=physician's assessment. III=SARI definition. F+/-=fever as part of case definition or not. T+/-=tachypnea part of case definition or not. W+/-=wheeze part of case definition or not. C+/-=crepitation part of case definition or not. XR=X-ray confirmed (radiologically confirmed pneumonia). CWI+/-=chest wall indrawing part of case definition or not. DS+/-=danger signs part of case definition or not. O2+/-=hypoxemia part of case definition or not. ICU=intensive care unit. MV=mechanical ventilation

† Data in parentheses are imputed

‡ NA=not available

Location (reference)	Case definition (codes)*	Denominator source	Specimen and diagnostic test*	Hospitalisation rate of RSV associated ALRI (per 1000 children per year)					
				0-5m	6-11m	0-11m	12-23m	24-59m	0-59m
Gipuzoka, Spain (urban, Jul 1996 - Jun 2000)[11]	II	Census-derived estimate	NPA; ELISA	36.8	14.2	25.5	3.6	0.5	6.2
Kiel, Germany (urban, 1996 - 97 to 2000 - 2001)[28]	II, XR, W+/-	Defined population base	NPA; RT-PCR	(21.5)		16.3			(4.4)
Multicentric, Germany (urban, Nov 1999 - Oct 2001)[20]	II	Census-derived estimate	NPS; PCR	(33.5)		(25.4)			(6.9)
Shropshire, United Kingdom (urban, 1996 - 1999)[29]	II	Census-derived estimate	NA; IF	(34.2)		(25.9)			(7.0)
Stockholm, Sweden (urban, 1987 - 1998)[30]	II	Census-derived estimate	NPW; IFA	(18.5)		14.0			(3.8)
Graz, Austria (urban, 1999 - 2000)[31]	II	Census-derived estimate	NPA; ELISA	(14.7)		(11.1)			(3.0)
England, United Kingdom (urban, Apr 1995 - Mar 1998)[8]	II	Census-derived estimate	NA	(37.4)		28.3			10.1
Netherlands (urban, 1997 - 2003)[32]	II	Census-derived estimate	NA; cell culture, IF	(12.5)		9.5			(2.6)
Banjul, Fajara and Sibanor, Gambia (urban and rural, Jan 1994 - Dec 1996)[33]	II	Census-derived estimate	NPA; IF	(23.4)		17.7			(4.8)
Mpumalanga, South Africa (rural, Apr 2000 - Mar 2001)[1]	II, CWI+/-, W+/-, T+/-	Census-derived estimate	NPA, NPW; ELISA	17.1		15.0			9.0
Takhli, Thailand (rural, 1999 - 2000)[34]	II, T+, CWI+, XR	Defined population base	NPA; IF	(43.0)		(32.6)			8.8
Hong Kong, China (urban, 1993 - 1997)[35]	II	Census-derived estimate	NPA, throat swab; DFA, virus isolation	(12.2)		(9.3)			2.5
Townsville (Queensland), Australia (urban and rural, Jan 1997 - Oct 1999)[§36]	II	Census-derived estimate	NA; IF, culture	(23.8)		18.0			(4.9)
Monroe and Davidson Counties in Tennessee, USA (urban and rural, Oct 2000 - Sep 2001)[7]	II	Census-derived estimate	nasal and throat swab; viral culture, RT-PCR	18.5	7.4	12.9	3.3	0.4	3.5
Milwaukee, USA (urban, Nov 1996 - Oct 1998)[37]	II	Census-derived estimate	NPS, throat swab; culture, ELISA	(28.4)		(21.5)			5.8
Tennessee, USA (urban, Jul 1989 - Jun 1993)[38]	II	Census-derived estimate	NA	88.2	30.1	63.0	7.3		(17.0)

§ Reported hospitalisation rate of an aboriginal population in a high-income country

Location (reference)	Case definition (codes)*	Denominator source	Specimen and diagnostic test*	Hospitalisation rate of RSV associated ALRI (per 1000 children per year)					
				0-5m	6-11m	0-11m	12-23m	24-59m	0-59m
Alaska, USA (rural, Jan 2000 - Dec 2001) §39	II	Census-derived estimate	NA	(45.4)		34.4			(9.3)
USA (urban and rural, Jan 2000 - Dec 2001)[39]	II	Census-derived estimate	NA	(36.2)		27.4			(7.4)
Nashville, Rochester and Cincinnati, USA (urban, Oct 2000 - Sep 2004)[6]	II, F+/-, W+/-	Census-derived estimate	nasal and throat swab; RT-PCR, culture	16.9	5.1	11.1	2.7	0.4	3.0
Kilifi birth cohort, Kenya (rural, Jan 2002 - May 2005) (Nokes and colleagues)	II, T+, CWI+/DS+ /O2+	Defined population base	NPA, nasal wash; DFA	20.0	6.0	11.6	5.4		(3.1)
CEMIC, Buenos Aires, Argentina (urban, 1st Jun 2008 - 31st Dec 2010) (Echavarria and colleagues)	II, ICU+/-, MV+/-	Defined population base	NPA; IFA	14.3		(16.8)		0.7	4.5
Buenos Aires, Argentina (urban and rural, 2011 - 2013) (Polack and colleagues)	II, O2+, W+, F+/-, ICU+/-, MV+/-	Census-derived estimate	NPA; PCR	64.5	33.4	48.9	9.1		(13.2)
Kishoregonj, Bogra, Comilla and Barisal, Bangladesh (urban and rural, 2010 - 2013) (Homaira and colleagues)	II, F+/-	Census-derived estimate	nasal and throat swab; RT-PCR	(18.7)		(14.1)			3.8
Belo Horizonte, Brazil (urban, 2011 - 2013) (Oliveira and colleagues)	II, F+, O2+/-, ICU+/-	Census-derived estimate	nasal swab; RT-PCR	(10.4)		7.9	1.3	0.9	2.2
Concepcion, Chile (urban and rural, Jan 2012 - Dec 2013) (Fasce and colleagues)	II, F+/-, ICU+/-	Census-derived estimate	NPA; IF	(11.0)		8.3	1.9	0.3	2.2
Iquique, Chile (urban and rural, Jan 2012 - Dec 2013) (Fasce and colleagues)	II, F+/-, ICU+/-	Census-derived estimate	NPA; IF	(30.4)		23.0	3.2	0.5	4.9
Jingzhou, China (urban and rural, Jan 2010 - Dec 2012) (Yu and colleagues)	III, T+/-, XR	Census-derived estimate	NPS; RT-PCR	3.5	6.6	5.2	11.2	3.4	5.4
Santa Ana, El Salvador (urban, 2008 - 2013) (Clara and colleagues)	III	Census-derived estimate	NPS; IFA	(52.5)		39.8	16.2	2.0	12.2
Gambia Western Region, Gambia (urban and rural, Jul 2007 - Jun 2008) (Howie and colleagues)	II, CWI+/-, W-, O2+/-	Defined population base	NPA; PCR	(32.2)	17.4	24.4	6.3	1.6	5.7
Santa Rosa, Guatemala (urban and rural, 2008 - 2013) (McCracken and colleagues)	II, CWI+/-, DS+/-	Census-derived estimate	NPS, OPS; RT-PCR	(34.9)		26.4	5.6	0.8	7.1

Location (reference)	Case definition (codes)*	Denominator source	Specimen and diagnostic test*	Hospitalisation rate of RSV associated ALRI (per 1000 children per year)					
				0-5m	6-11m	0-11m	12-23m	24-59m	0-59m
Quetzaltenango, Guatemala (urban and rural, 2009 - 2013) (McCracken and colleagues)	II, CWI+/-, DS+/-	Census-derived estimate	NPS, OPS; RT-PCR	(19.1)		14.4	3.3	0.4	3.8
Ballabhgarh, India (rural, Jan 2010 - Dec 2012) (Broor and colleagues)	II, DS+/-	Defined population base	NPS; RT-PCR	6.3	2.0	3.8	1.0	0.1	1.0
Pune, India (rural, May 2009 - Apr 2013) (Chadha and colleagues)	II	Census-derived estimate	NPS; RT-PCR	12.2	22.1	18.5	11.1	2.2	5.3
Lombok, Indonesia (urban and rural, 2000 - 2002) (Gessner and colleagues)	II, CWI+	Defined population base	nasal wash; ELISA	14.0	12.3	13.1	2.6		(3.5)
Kilifi hospital study, Kenya (urban and rural, Jan 2002 - Dec 2010) (Nokes and colleagues)	II, CWI+, O2+/DS+	Census-derived estimate	OPS, NPS, nasal wash; DFA	27.2	11.1	19.3	3.4	0.6	4.9
Bondo district, Kenya (rural, Jan 2007 - Jun 2009) (Feikin and colleagues)	II, O2+/DS+	Census-derived estimate	NPS, OPS; RT-PCR	52.6	25.0	32.0	6.3	7.1	16.5
Manhiça, Mozambique (rural, 20th Sep 2006 - 19th Sep 2007) (Bassat and colleagues)	II, T+	Census-derived estimate	NPA; RT-PCR	14.5	6.6	11.0	2.8	0.6	3.2
Manhiça, Mozambique (rural, 1st Jan 2011 - 30th Jun 2014) (Bassat and colleagues)	II, T+, O2+/-	Defined population base	NPA; RT-PCR	4.4	1.9	3.2	1.2	0.4	1.1
Utrecht, Netherlands (urban, 2001 - 2010) (Bont and colleagues)	II	Defined population base	NPS, NPW; PCR, IF	(11.1)		8.4			(2.3)
41 sites in Netherlands (urban and rural, 2008 - 2013) (Bont and colleagues)	II, ICU+/-	Defined population base	NPS, NPW; PCR, IF	(53.5)		40.5			(10.9)
David City, Panama (urban, Jan 2011 - Dec 2013) (Jara and colleagues)	II	Defined population base	NPS, OPS; IFA, RT-PCR	(24.3)		18.4			5.6
Tagbilaran and 6 rural sites, Philippines (urban and rural, 5th Jul 2000 - 31st Dec 2004) (Lucero and colleagues)	II, CWI+, DS+/-	Defined population base	NPA, nasal swab; culture, PCR	73.1	54.8	62.4	23.1		(16.8)
Gauteng province, South Africa (urban, 2009 - 2012) (Cohen and colleagues)	II	Defined population base	NPA; PCR	(38.9)		29.4	6.8	1.3	7.8
KwaZulu-Natal province, South Africa (periurban, 2010 - 2014) (Cohen and colleagues)	II	Defined population base	NPA; PCR	(27.5)		20.8	2.6	0.7	5.1
Klerksdorp site, South Africa (periurban, Jan 2011 - Dec 2014) (Cohen and colleagues)	II	Defined population base	NPA; PCR	(16.3)		12.3			3.3
Soweto, South Africa (urban, Mar 1998 - Oct 2005) (Madhi and colleagues)	II, CWI+, O2+/-	Defined population base	NPA; IF	33.1	10.2	20.0	4.0	0.5	4.7
Paarl, South Africa (periurban, Mar 2012 - Dec 2014) (Zar and colleagues)	II, CWI+, T+, DS+/-	Defined population base	NPS; RT-PCR	146.7	29.9	91.5	4.9		(24.7)

Location (reference)	Case definition (codes)*	Denominator source	Specimen and diagnostic test*	Hospitalisation rate of RSV associated ALRI (per 1000 children per year)					
				0-5m	6-11m	0-11m	12-23m	24-59m	0-59m
Maela Camp, Tak Province, Thailand (rural, Nov 2007 - Oct 2010) (Turner and colleagues)	II, CWI+, T+	Defined population base	NPA; RT-PCR	135.7	73.1	105.0	61.9		(28.4)
Sa Kaeo and Nakhon Phanom, Thailand (rural, Jan 2008 - Dec 2011) (Thamthitiwat and colleagues)	II, F+/-, T+/-, CWI+, O2+/-	Census-derived estimate	NPS; PCR	12.2	18.6	15.4	15.3	6.3	9.8
Tone district, Togo (rural, Aug 2011 - Dec 2013) (Gessner and colleagues)	II, CWI+	Census-derived estimate	nasal wash; RT-PCR	0.4	0.3	0.4	0.2	0.0	0.1
Alaska, USA (rural, Jul 1994 - Jun 2012) (Singleton and colleagues) §	II, MV+/-	Census-derived estimate	NPS; ELISA, DFA, culture	202.0	121.1	161.6	39.8		(43.6)
Colorado, USA (urban and rural, Jan 2008 - Jun 2013) (Simoes and colleagues)	II	Census-derived estimate	nasal wash; PCR	(23.8)		18.1	5.8	1.6	5.7
Navajo and WMA, USA - MEDI (rural, Oct 2004 - Dec 2010) (O'Brien and colleagues) §	II, O2+/-	Defined population base	NPS; RT-PCR	314.4	236.3	292.3			(78.9)
Navajo and WMA, USA - EPI (rural, Oct 1997 - Mar 2000) (O'Brien and colleagues) §	II, O2+/-	Defined population base	NPA; ELISA	230.2	158.9	194.4	81.7		(52.5)
Nha Trang, Vietnam (urban and rural, Feb 2007 - Dec 2012) (Yoshida and colleagues)	II, CWI+/DS+	Census-derived estimate	NPS; RT-PCR	9.3	3.8	7.5	4.1	0.8	2.8
Memphis, Nashville and Salt Lake City, USA (urban, Jul 2010 - Jun 2012)[40]	II, XR	Census-derived estimate	NPS, OPS; RT-PCR	(5.8)		(4.4)		0.7	(1.2)
Spain (urban, Jan 1997 - Dec 2011)[5]	II	Census-derived estimate	NA	(54.6)		41.4	8.0	2.2	10.7
Karemo, Kenya (rural, Aug 2009 - Jul 2012)[41]	III	Census-derived estimate	NPS, OPS; RT-PCR	13.4	14.0	(19.3)	8.1	2.0	5.2
Ho Chi Minh, Vietnam (urban, Jul 2009 - Dec 2013)[42]	II	Defined population base	NPS; RT-PCR	(10.6)		8.0			(2.2)
Dong Thap, Vietnam (urban and rural, Aug 2009 - Dec 2012)[42]	II	Defined population base	NPS; RT-PCR	(40.8)		30.9			(8.3)
USA (urban, 1997 - 2000)[43]	II	Census-derived estimate	NA	(30.0)		22.7			(6.1)
USA (urban, 2000)[9]	II	Census-derived estimate	NA	(22.9)		17.4			4.5
**Valencia, Spain (urban, Jan 2009 - Dec 2012)[44]**	II	Defined population base	NA	31.6	3.6	19.5	0.7		(5.3)

Location (reference)	Case definition (codes)*	Denominator source	Specimen and diagnostic test*	Hospitalisation rate of RSV associated ALRI (per 1000 children per year)					
				0-5m	6-11m	0-11m	12-23m	24-59m	0-59m
**New SouthWales, Australia (urban, 2001 - 2010)[45]**	II	Defined population base	NA	(24.2)		(18.3)			4.9
**Gothenburg, Sweden (urban, 2004 - 2011)[46]**	II	Census-derived estimate	NA; PCR, IF	(23.0)		17.4			4.2

Supplementary table 6: Description of all studies reporting proportion of ALRI hospitalisations in children younger than 5 years positive for RSV

Location (reference)	Case definition (codes)*	Specimen and diagnostic test*	Proportion of RSV associated hospitalised ALRI (%)			
			0-11m	0-23m	0-35m	0-59m
Subiaco, Australia (urban, Jan 2000 - Dec 2005)[47]	II	NPA; DFA, RT-PCR, viral culture	54.4	51.4		
Hangzhou, China (urban, Jan 2001 - Dec 2006)[48]	II	NPA; DFA	30.1		27.1	
Lanzhou, China (urban, 1st Jan - 31st Dec 2011)[49]	II, F+/-, W+/-, XR	throat swab; RT-PCR	19.3			
Lanzhou, China (urban, Oct 2004 - Oct 2005)[50]	II, F+/-, XR	NPA, serum; DFA, ELISA	23.4		20.3	19.2
Shanghai, China (urban, May 2009 - Jul 2010)[51]	II, F+	NPS; RT-PCR			11.6	
Hong Kong, China (urban, 1st Jan 2004 - 31st Dec 2004)[52]	II	NPA; DFA			11.6	
Hong Kong, China (urban, Nov 2005 - Oct 2006)[53]	II, W+/-, F+/-	NPA, nasal swab; DFA, RT-PCR, viral culture				8.4
Taiwan, China (urban, Jan 2001 - Dec 2003)[54]	II	NPA; virus culture, IF	26.1	24.3		23.5
Shizuoka, Japan (urban, 1st Jul 1997 - 30th Jun 2000)[55]	II, W+/-, XR	NPA; ELISA			31.4	
Tokyo, Japan (urban, Mar 2007 - Jul 2009)[56]	II, W+/-, F+/-, XR	NPS; RT-PCR		27.6		
Japan (urban, Apr 2008 - Apr 2009)[57]	II, F+, XR	NPS; RT-PCR	26.9	28.7		26.1
Nagasaki, Japan (urban, Apr 2009 - Mar 2010)[58]	II, XR	NPS; IF, RT-PCR				55.9
Seoul, Korea (urban, Jan - Dec 2004)[59]	II, W+, XR	NPA; RT-PCR				29.5
Seoul, Korea (urban, Jul 2004 - Jan 2006)[60]	II, XR, W+	NPA; DFA				20.6
Seoul, Korea (urban, Jan 2007 - Feb 2011)[61]	II	NPA; RT-PCR			67.7	
Kuala Lumpur, Malaysia (urban, Jan 1982 - Dec 1997)[62]	II	NPA; IFA, culture	19.2	18.4		

* NPA=nasopharyngeal aspirate. NPS=nasopharyngeal swab. NPW=nasopharyngeal wash. OPS=oropharyngeal swab. RT-PCR=reverse transcriptase polymerase chain reaction. IFA=indirect immunofluorescent antibody test. DFA=direct immunofluorescent antibody test. ELISA=enzyme-linked immunosorbent assay. IF=immunofluorescence. II=physician's assessment. III=SARI definition. F+/-=fever as part of case definition or not. T+/-=tachypnea part of case definition or not. W+/-=wheeze part of case definition or not. C+/-=crepitation part of case definition or not. XR=X-ray confirmed (radiologically confirmed pneumonia). CWI+/-=chest wall indrawing part of case definition or not. DS+/-=danger signs part of case definition or not. O2+/-=hypoxemia part of case definition or not. ICU=intensive care unit. MV=mechanical ventilation.

Location (reference)	Case definition (codes)*	Specimen and diagnostic test*	Proportion of RSV associated hospitalised ALRI (%)			
			0-11m	0-23m	0-35m	0-59m
Ho Chi Minh City, Vietnam (urban, Nov 2004 - Jan 2008)[63]	II, T+, CWI+/-, ICU+/-	nasal swab, throat swab, NPA; RT-PCR	21.4			24.4
Ho Chi Minh City, Vietnam (urban, Apr 2010 - May 2011)[64]	II, XR	NPS; RT-PCR		24.7		
Pune, India (urban, Feb 2002 - Dec 2004)[65]	II	NPA; IF	46.0		42.5	
Kolkata, India (urban, Apr 2010 - Mar 2011)[66]	III, CWI+, W+, C+, T+	nasal and throat swabs; RT-PCR	21.0			16.7
Lucknow, India (urban, Jun 2011 - May 2012)[67]	II, CWI+	NPA; RT-PCR	34.3			25.2
Khon Kaen, Thailand (urban, Aug 1992 - Nov 1994)[68]	II, CWI+, XR	NPA; virus culture, IF, ELISA				29.0
Nakhon Sawan, Thailand (rural, Nov 1998 - Feb 2001)[69]	II, CWI+	NPA; IFA				34.2
Khon Kaen, Thailand (urban, Apr 2002 - Aug 2004)[70]	II, W+, T+	NPA; RT-PCR	67.6	64.7		
Tak Province, Thailand (rural, Apr 2009 - Sep 2011)[71]	II, CWI+/-, DS+/-	NPA; RT-PCR	39.1			
Seeb, Oman (urban, Dec 2007 - Dec 2008)[72]	II	NPA; IF, RT-PCR				21.6
Zarqa, Jordan (urban, Jan 1997 - May 1999)[73]	II	NPW; DFA, RT-PCR		25.5		
Amman, Jordan (urban, Sep 2002 - Mar 2004)[74]	II	NPA; DFA	15.6	12.5		
Riyadh, Saudi Arabia (urban, Apr 1993 - Mar 1996)[75]	II, F+, W+/-, XR	NPA; IFA				28.9
Riyadh, Saudi Arabia (urban, Jan 2005 - Dec 2010)[76]	II	NPA; DFA	48.8		48.4	47.4
Abha, Saudi Arabia (urban, Oct 1997 - Sep 2001)[77]	II	NPA; ELISA, IFA				35.3
Zagreb County, Croatia (urban, 1994 - 2005)[78]	II	NPA; DFA, virus isolation	50.9	47.4		45.8
Zagreb County, Croatia (urban, Aug 2006 - Aug 2007)[79]	II	NPA; DFA	48.1	47.0		46.5
Turku, Finland (urban, Jan 1993 - Dec 1995)[80]	II, F+, XR	NPA; ELISA, culture		46.3		
Montpellier, France (urban, 1st Nov 2003 - 31st Oct 2004)[81]	II	NPA; DFA, virus culture	33.8	30.5	29.4	
Kiel, Germany (urban, Jul 1996 - Jun 2001)[82]	II	NPA; ELISA, RT-PCR	28.2	23.6		19.8
Germany (urban, Nov 1999 - Oct 2001 and Oct 2002 - Jun 2005)[83]	II	NPA; RT-PCR, ELISA	42.8	36.2	34.1	
Athens, Greece (urban, 2008 - 2009)[84]	II	NPA; IF				28.5
Be'er Sheva, Israel (urban, Nov 2001 - Oct 2002)[85]	II	nasal wash; DFA				19.6
Be'er Sheva, Israel (urban, Nov 2001 - Oct 2005)[86]	II, XR	NPW; DFA, culture				28.0
Pisa, Italy (urban, Jan 2000 - May 2006)[87]	II	nasal swab; DFA		31.1		

Location (reference)	Case definition (codes)*	Specimen and diagnostic test*	Proportion of RSV associated hospitalised ALRI (%)			
			0-11m	0-23m	0-35m	0-59m
Milan, Italy (urban, Oct 2004 - Sep 2006)[88]	II, W+/-	NPA; RT-PCR		28.0		
Milan, Italy (urban, 1st Dec 2008 - 31st Dec 2009)[89]	II, W+/-	OPS; RT-PCR	52.0	42.6		37.0
Enschede, Netherlands (urban, 2006 - 2007)[90]	II, W+/-, F+/-	NPW; RT-PCR		48.3		
Palma, Spain (urban, Jan 1995 - Dec 2006)[91]	II	NPA, NPW; ELISA, culture		62.7		
Madrid, Spain (urban, Oct 2000 - Jun 2005)[92]	II	NPA; IFA, RT-PCR		45.3		
Madrid, Spain (urban, Sep 2005 - Aug 2008)[93]	II, W-, ICU+/-	NPA; RT-PCR		61.3		
Malmo, Sweden (urban, 1998 - 1999)[94]	II	NPA; IF	54.5			
Lausanne and Geneva, Switzerland (urban, Mar 2003 - Dec 2005)[95]	II, CWI+, W-	NPA, serum; RT-PCR, IFA, ELISA, serology				13.1
Turkey (urban, May 2008 - Sep 2010)[96]	II	NPA, NPS, nasal wash, nasal swab; IF		16.9		
Buenos Aires and Santa Fe, Argentina (urban, 1990 - 1996)[97]	II, T+/-, W+/-, XR	NPA; IFA		27.0		
Buenos Aires, Argentina (urban, 1999 - 2004)[98]	II	NPA; RT-PCR, DFA				31.6
Pelotas, Brazil (urban, 1st Aug 1997 - 31st Jul 1998)[99]	II, CWI+	NPA; DFA	30.8			
Bahia, Brazil (urban, Jan - Dec 1998)[100]	II	NPA; IFA				23.8
Sao Paulo, Brazil (urban, Mar 1999 - Jun 2000)[101]	II, W+, XR	NPA, nasal swab; IFA, virus culture, RT-PCR	60.9			
Sao Paulo, Brazil (urban, Jan 2003 - Dec 2006)[102]	II	NPA, nasal swab; RT-PCR	45.4			42.0
Sao Paulo, Brazil (urban, Jan 2006 - Dec 2007)[103]	II, W-	NPA; IFA	63.5			
Bahia, Brazil (urban, Sep 2003 - May 2005)[104]	II, XR	NPA, serum; IF, ELISA				15.2
Sao Paulo, Brazil (urban, May 2004 - Sep 2005)[105]	II, T+/-, W+/-	NPA; RT-PCR	40.4	34.1		
Sao Paulo, Brazil (urban, Feb 2005 - Sep 2006)[106]	II, W+/-	NPA; IFA	30.1			28.6
Sao Paulo, Brazil (urban, Mar 2008 - Aug 2011)[107]	II, CWI+	NPA; RT-PCR		27.3		
Belem, Brazil (urban, Nov 2006 - Oct 2007)[108]	II, F+/-, XR	NPA; DFA, RT-PCR			23.1	
Porto Alegre, Brazil (urban, Sep 2009 - Sep 2010)[109]	II, W-	NPA; DFA	53.5			
Iqaluit, Canada (urban, 28th Jan 2002 - 27th Mar 2003)[110]	II	NPA; ELISA		51.2		

Location (reference)	Case definition (codes)*	Specimen and diagnostic test*	Proportion of RSV associated hospitalised ALRI (%)			
			0-11m	0-23m	0-35m	0-59m
British Columbia, Canada (urban, Apr 2008 - Mar 2010)[111]	II	NA†	48.1			
Medellin, Colombia (urban and rural, Apr 1994 - Apr 1995)[112]	II	NPW; DFA	41.7			
Colombia (urban, Apr 2005 - Apr 2006)[113]	II	NPA; IF	30.1			
San Luis Potosi, Mexico (urban, 1st May 2003 - 30th Apr 2005)[114]	II	NPW; DFA	26.9	25.9	24.8	
Alaska, USA (rural, 1st Oct 1991 - 30th Sep 1993)‡[115]	II	NA; ELISA, IF, culture		38.4		
California, USA (urban, 1996 - 2004)[116]	II	NA; DFA		56.2		
Dallas, Texas, USA (urban, Jan 2002 - Dec 2007)[117]	II	NPA; IF, DFA, virus culture		66.3		
New York, USA (urban, Oct 2007 - May 2010)[118]	II, F+	NPS; RT-PCR, ELISA, virus culture		53.7		
Kumasi, Ghana (urban, Jan 2008 - Dec 2008)[119]	II, CWI+, DS+/-	NPS; RT-PCR		14.6		14.1
Rarieda, Kenya (rural, 1st Mar 2007 - 28th Feb 2010)[120]	III, DS+/CWI+	NPS, OPS; RT-PCR				22.6
Kilifi, Kenya (rural, Jan - Dec 2010)[121]	II, CWI+	NPA, OPS, sputum; RT-PCR				26.5
Bondo district, Kenya (rural, Aug 2008 - Dec 2010)[122]	II, CWI+/DS+/O2+	NPS, OPS; RT-PCR				17.1
Santiago, Chile (urban, Jan 1989 - Dec 2000)[123]	II, W-	NPA; IFA		29.0		
Cape Town, South Africa (urban, Jun 1995 - Aug 1996)[124]	II, W+/-	NPA; ELISA		15.8		
Manhica District, Mozambique (rural, Oct 1998 - May 2000)[125]	II, CWI+/-, W+/-, T+/-	NPA; ELISA	10.7			
Athens, Greece (urban, Feb 1997 - Jun 2000)[126]	II, XR, W+/-, T+/-	NPW; DFA	61.5			
Buenos Aires, Argentina (urban, May 1991 - Dec 1992)[127]	II, CWI+/-, XR, W+/-, T+/-	NPA; IFA		38.6		
Rio de Janeiro, Brazil (rural, Jan 1987 - Dec 1989)[19]	II, T+/-, W+/-	NPA; IFA, culture				40.5
Beijing, China (urban, Feb 2001 - Mar 2003)[128]	II	NPA; virus culture, IFA	44.9		39.0	36.5
Beijing, China (urban, Mar 2010 - Feb 2012)[129]	II	NPA, throat swab; RT-PCR	56.3		49.1	41.8

† NA= Not available

‡ Reported data from aboriginal population in a high-income country

Location (reference)	Case definition (codes)*	Specimen and diagnostic test*	Proportion of RSV associated hospitalised ALRI (%)			
			0-11m	0-23m	0-35m	0-59m
Changsha, China (urban, Sep 2007 - Aug 2008)[130]	II, F+, T+	NPA; RT-PCR	42.0		37.1	37.0
Changsha, China (urban, Sep 2007 - Aug 2008)[131]	II	NPA; RT-PCR	30.1		27.6	27.6
Changsha, China (urban, Jun 2011 - Jun 2012)[132]	II	NPA; DFA	55.1		53.7	53.9
Chaozhou, China (urban, Jun 2011 - Oct 2012)[133]	II	NPA; IFA	9.3		6.9	
Chengdu, China (urban, Jan - Dec 2007)[134]	II	NPA; DFA	29.8		26.4	
Chengdu, China (urban, Mar 2010 - Feb 2011)[135]	II	NPA; RT-PCR		30.6		
Chenzhou, China (urban, Jan - Dec 2010)[136]	II	NPA; PCR	21.5		18.2	
Chongqing, China (urban, Apr 2003 - Oct 2007)[137]	II	NPA; DFA	36.4		29.6	
Chongqing, China (urban, Apr 2008 - Mar 2009)[138]	II	NPA; RT-PCR	31.7	29.3	28.7	
Chongqing, China (urban, Feb 2009 - Mar 2011)[139]	II	NPA; DFA				
Chongqing, China (urban, Jan 2009 - Dec 2011)[140]	II	NPA; DFA	34.5		31.9	
Dongguan, China (urban, Mar 2011 - May 2012)[141]	II	NPA; DFA	32.1		23.2	
Chengdu, China (urban, Jan 2007 - Dec 2009)[142]	II	NPA; IF	25.5		27.5	
Fuyang, China (urban, Jan 2003 - Dec 2004)[143]	II	NPA; DFA				
Guangzhou, China (urban, Oct 2009 - Sep 2010)[144]	II	NPS; RT-PCR	10.7		8.5	7.5
Guiyang, China (urban, Jan 2010 - May 2011)[145]	II	NPA; IF			21.0	
Guiyang, China (urban, Jun 2006 - Apr 2011)[146]	II	NPA; DFA	27.1		24.1	
Haikou, China (urban, Jan 2007 - Oct 2008)[147]	II	NPA; RT-PCR	41.7		39.7	
Hangzhou, China (urban, 2000 - 2001)[148]	II	NPA; IF	34.9		29.5	
Hangzhou, Chin (urban, 2001 - 2003)[149]	II	NPA; DFA				
Jiaxing, China (urban, Dec 2008 - Dec 2009)[150]	II	NPA; RT-PCR, DFA	16.8	15.8		
Jiaxing, China (urban, Aug 2010 - Aug 2012)[151]	II	NPA; DFA	18.6		14.6	
Kunming, China (urban, Oct 2005 - Oct 2007)[152]	II	NPA; DFA	28.5		25.9	
Kunming, China (urban, Sep 2009 - Sep 2010)[153]	II	NPA; RT-PCR	46.2	42.9	40.8	40.2
Yueqing, China (urban, Jan 2006 - Dec 2010)[154]	II	NPA; DFA	32.0		23.2	19.3
Liaocheng, China (urban, Oct 2007 - Oct 2008)[155]	II	NPA; DFA	43.1		38.9	

Location (reference)	Case definition (codes)*	Specimen and diagnostic test*	Proportion of RSV associated hospitalised ALRI (%)			
			0-11m	0-23m	0-35m	0-59m
Linyi, China (urban, Dec 2010 - Dec 2011)[156]	II	NPA; RT-PCR	33.2	31.5		
Nanjing, China (urban, Apri 2006 - Mar 2007)[157]	II	NPA; DFA	28.3	20.9		17.1
Nanjing, China (urban, Sep 2008 - Aug 2009)[158]	II	NPA; DFA	33.1		29.0	
Nanjing, China (urban, Jan 2009 - Dec 2011)[159]	II	NPA; DFA	36.8		34.1	
Shanghai, China (urban, May 2001 - Apr 2002)[160]	II	NPA; IF	25.3		21.1	
Shanghai, China (urban, Jan - Dec 2000)[161]	II	NPA; IF	21.1		19.1	18.6
Shanghai, China (urban, Oct 2002 - Apr 2004)[162]	II	NPA; DFA	29.8		20.4	19.0
Shanghai, China (urban, 2003 - 2006)[163]	II	NPA; DFA	21.4		23.0	21.6
Shanghai, China (urban, Jan 2009 - Mar 2010)[164]	II	NPA, throat swab; RT-PCR	28.1		25.6	
Shanghai, China (urban, Mar 2011 - Feb 2012)[165]	II	NPA; DFA			10.1	
Shangluo, China (urban, May 2011 - May 2012)[166]	II	NPA; IF	43.3		40.6	39.7
Shantou, China (urban, Jan 2007 - Dec 2007)[167]	II	NPA; RT-PCR				19.1
Suzhou, China (urban, Sep 2005 - Oct 2011)[168]	II	NPA; DFA	22.4		18.9	17.1
Tianjin, China (urban, Jan 2002 - Mar 2004)[169]	II	NPA; IFA	9.8		9.4	
Tianjin, China (urban, Jan 2010 - Jun 2011)[170]	II	NPA; DFA	7.5		6.5	
Weifang, China (urban, Nov 2007 - Nov 2008)[171]	II	NPA, NPS; DFA	31.0		25.8	
Wenzhou, China (urban, Jan 2003 - Jan 2005)[172]	II	NPA; DFA	42.8		39.5	
Wenzhou, China (urban, Nov 2003 - Feb 2005)[173]	II	NPS; IFA			27.5	
Wenzhou, China (urban, Nov 2004 - Nov 2006)[174]	II	NPA; DFA	39.5		39.6	
Wenzhou, China (urban, Jan 2007 - Dec 2008)[175]	II	NPA; DFA	38.1		35.6	
Wenzhou, China (urban, Feb 2009 - Jan 2010)[176]	II	NPA; DFA	34.1		30.4	28.2
Wenzhou, China (urban, Jan - Dec 2010)[177]	II	NPA; IF				
Xian, China (urban, 1994 - 1997)[178]	II	NPA; IF	27.4		25.8	
Yancheng, China (urban, Jun 2011 - Jul 2012)[179]	II	NPA; DFA	22.1		20.0	
Zhongshan, China (urban, Apr 2011 - Mar 2012)[180]	II	NPS; IF	17.8			
Zhuzhou, China (urban, Jan - Dec 2011)[181]	II	NPA; DFA	26.7		25.3	22.5

Location (reference)	Case definition (codes)*	Specimen and diagnostic test*	Proportion of RSV associated hospitalised ALRI (%)			
			0-11m	0-23m	0-35m	0-59m
Buenos Aires, Argentina (urban, 1st Jun 2008 - 31st Dec 2010) (Echavarria and colleagues)	II, ICU+/-, MV+/-	NPA; IFA	43.0	36.8	34.8	33.3
Buenos Aires, Argentina (urban, 2001 - 2013) (Gentile and colleagues)	II, MV+/-	NPA; IFA	42.7	40.4	39.8	38.9
Takeo and Kampong Cham, Cambodia (urban, Apr 2007 - Feb 2010) (Goyet and colleagues)	II, F+, T+	NPS, throat swab; RT-PCR	22.6	20.5	20.2	18.8
Chillan, Chile (urban and rural, 2010 - 2013) (Fasce and colleagues)	II	NPA; IF	27.2			23.7
Osorno, Chile (urban and rural, 2010 - 2013) (Fasce and colleagues)	II	NPA; IF	25.4			22.3
Punta Arenas, Chile (urban and rural, 2010 - 2013) (Fasce and colleagues)	II	NPA; IF	60.8			56.4
Santiago, Chile (urban, 2010 - 2013) (Fasce and colleagues)	II	NPA; IF	33.5			28.2
Valparaiso, Chile (urban mostly, 2010 - 2013) (Fasce and colleagues)	II	NPA; IF	20.1			16.0
China (urban and rural, Jan 2009 - Sep 2013) (Yu and colleagues)	II, XR, W+/-, F+/-	NPA, NPS; PCR	27.0	24.9	23.5	21.6
Beijing, China (urban, Jan 2011 - Dec 2012) (GABRIEL)	II, T+, W-, XR	NPA, NPS; RT-PCR	73.8	53.8	42.2	33.1
Ulaanbaatar, Mongolia (urban, Sep 2011 - Oct 2012) (GABRIEL)	II, T+, W-, XR	NPA, NPS; RT-PCR	21.4	21.5	24.7	22.8
Phnom Penh, Cambodia (urban, Oct 2010 - Jan 2013) (GABRIEL)	II, T+, W-, XR	NPA, NPS; RT-PCR	21.8	22.6	22.8	21.7
Lucknow, India (urban and rural, Jun 2012 - Dec 2013) (GABRIEL)	II, T+, W-, XR	NPA, NPS; RT-PCR	9.1	6.4	6.2	5.7
Antananarivo, Madagascar (urban and rural, Dec 2010 - Feb 2013) (GABRIEL)	II, T+, W-, XR	NPA, NPS; RT-PCR	10.9	15.9	15.0	13.2
Asuncion, Paraguay (urban, Jul 2010 - May 2013) (GABRIEL)	II, T+, W-, XR	NPA, NPS; RT-PCR	25.6	16.9	14.9	14.3
Bamako, Mali (urban, Jul 2011 - Nov 2012) (GABRIEL)	II, T+, W-, XR	NPA, NPS; RT-PCR	43.6	36.4	30.9	26.5
Berlin, Germany (urban, 1st Apr 2010 - 31st Mar 2014) (Rath and colleagues)	II, F+, ICU+/-	NPS, NPA; RT-PCR	38.8	31.2	29.5	27.7
Tehran, Iran (urban, Mar 2008 - May 2009)[182]	II	throat swab and wash; PCR	20.5	18.9	17.6	17.6
Iran (urban, Nov 2007 - Apr 2013)[183]	II	throat swab; RT-PCR	24.6	24.7		
Amman, Jordan (urban, Mar 2010 - Mar 2013) (Khuri and colleagues)	II, T+, O2+/ICU/MV	nasal and throat swab; PCR	55.7	52.5		
Lwak, Kenya (rural, 2007 - 2011) (Montgomery and colleagues)	II, CWI+/DS+/O2+	NPS, OPS; RT-PCR	32.9	25.9	23.3	21.5
Rabat, Morocco (urban, Nov 2010 - Dec 2011) (Bassat and colleagues)	II, CWI+, T+	NPA; RT-PCR	29.3	22.3	19.9	17.3
Kathmandu, Nepal (urban, Jan 2006 - Jun 2008) (Basnet and colleagues)	II, CWI+	NPA; RT-PCR	15.2	14.1	13.8	

Location (reference)	Case definition (codes)*	Specimen and diagnostic test*	Proportion of RSV associated hospitalised ALRI (%)			
			0-11m	0-23m	0-35m	0-59m
Karachi, Pakistan (urban, Aug 2009 - Jul 2012) (Ali and colleagues)	II	throat swab; RT-PCR	22.1	21.2	20.5	19.4
Basse, Gambia (rural, 3rd Nov 2011 - 2nd Nov 2013) (PERCH)	II, CWI+, DS+/-	NPS, OPS; PCR	25.5	21.9	20.3	19.3
Kilifi, Kenya (urban, 15th Aug 2011 - 15th Nov 2013) (PERCH)	II, CWI+, DS+/-	NPS, OPS; PCR	33.7	27.0	25.2	23.7
Bamako, Mali (urban, 3rd Jan 2012 - 14th Jan 2014) (PERCH)	II, CWI+, DS+/-	NPS, OPS; PCR	32.0	27.5	26.4	25.4
Nakhon Phanom and Sa Kaeo, Thailand (rural, 1st Jan 2012 - 31st Jan 2014) (PERCH)	II, CWI+, DS+/-	NPS, OPS; PCR	26.4	26.2	29.1	27.3
Lusaka, Zambia (urban, 10th Oct 2011 - 31st Oct 2013) (PERCH)	II, CWI+, DS+/-	NPS, OPS; PCR	20.4	19.6	18.9	18.7
Dhaka, Bangladesh (urban, 1st Jan 2012 - 31st Dec 2013) (PERCH)	II, CWI+, DS+/-	NPS, OPS; PCR	21.3	19.9	19.6	18.7
Matlab, Bangladesh (rural, 1st Jan 2012 - 31st Dec 2013) (PERCH)	II, CWI+, DS+/-	NPS, OPS; PCR	53.6	39.0	35.8	33.3
Tacloban, Philippines (rural, May 2008 - Jul 2012) (Lupisan and colleagues)	II, CWI+, DS+/-	NPS, serum; PCR, virus isolation, culture, serology	21.6	18.9	17.9	17.0
Mpumalanga, South Africa (rural, Jan 2010 - Dec 2014) (Cohen and colleagues)	II	NPA; PCR	25.9	23.2	22.4	21.3
Nanjing, China (urban, Jan 2013 - Dec 2013)[184]	II	NPA; DFA	26.5		11.5	
Guangdong, China (urban, Jul 2010 - Jul 2012)[185]	II	NPA; RT-PCR	6.1		8.8	7.5
Yanting, China (urban, Jan 2011 - Dec 2012)[186]	II	NPA; RT-PCR	56.3		49.0	41.7
Guiyang, China (urban, Jan 2012 - Dec 2013)[187]	II	NPA; DFA	40.6		32.4	
Qingyuan, China (urban, Aug 2012 - Apr 2014)[188]	II	NPA; RT-PCR	7.9		7.4	
Guangzhou, China (urban, Jun 2012 - Jun 2013)[189]	II	NPA; RT-PCR	7.2		7.2	6.8
Guangxi, China (urban, Jan 2013 - Dec 2013)[190]	II	NPA; DFA	13.9		13.0	12.5
Huzhou, China (urban, Jan 2011 - Dec 2013)[191]	II	NPA; DFA	18.4		17.2	16.8
Foshan, China (urban, Feb 2012 - Jan 2014)[192]	II	NPA; DFA	16.6	15.4		13.0
Wuhan, China (urban, Jan 2011 - Dec 2012)[193]	II	NPA; DFA	20.2			
Wenzhou, China (urban, Jan 2013 - Dec 2013)[194]	II	NPA; RT-PCR	42.6			42.4
Wenzhou, China (urban, Dec 2012 - Nov 2013)[195]	II	NPA; DFA	20.0		16.3	13.0
Chongqing, China (urban, Feb 2013 - Apr 2014)[196]	II	NPA; DFA	11.7		10.4	9.3
Ningxia, China (urban, Jan 2010 - Dec 2013)[197]	II	NPA; DFA	91.8	82.5	76.8	
Lishui, China (urban, Jan 2010 - Mar 2013)[198]	II	NPA; DFA	24.3		21.7	

Location (reference)	Case definition (codes)*	Specimen and diagnostic test*	Proportion of RSV associated hospitalised ALRI (%)			
			0-11m	0-23m	0-35m	0-59m
Nantong, China (urban, Jan 2012 - Mar 2013)[199]	II	NPA; DFA	39.7		30.8	28.5
Quanzhou, China (urban, Jan 2012 - Jan 2013)[200]	II	NPS; DFA	37.7		31.0	25.6
Suzhou, China (urban, Jan 2009 - Dec 2013)[201]	II	NPS; DFA	18.9	17.6	17.1	15.8
Changsha, China (urban, Apr 2013 - Mar 2014)[202]	II	NPA; DFA	50.7		46.4	45.0
Shenzhen, China (urban, Jul 2007 - Jun 2010)[203]	II, F+	NPA; RT-PCR	16.3	15.6		
Soma, Japan (urban, Feb 2008 - Aug 2009)[204]	II	NPA; RT-PCR			34.2	
Tokyo, Japan (urban, Apr 2007 - Mar 2012)[205]	II	nasal swab; RT-PCR		30.8		
Baguio, Philippines (urban, Apr 2009 - Dec 2011)[206]	III	NPS, OPS; RT-PCR		31.7		28.4
Riga, Latvia (urban, Jul 2009 - Jun 2012)[207]	II	NPA; RT-PCR		42.5		
Mirzapur, Bangladesh (both, Jun 2006 - Sep 2007)[208]	II	nasal wash; RT-PCR				
Beirut, Lebanon (urban, Oct 2012 - Mar 2014)[209]	II	NPW; IF		25.8		
England, UK (urban, Apr 2007 - Mar 2008)[210]	II	NA	28.0			
Tehran, Iran (urban, Jan 2012 - Dec 2012)[211]	II	NPA; RT-PCR				17.2
Taiwan, China (urban, Jan 2009 - Mar 2011)[212]	II	NPA; culture, IF		43.4		
Ouagadougou, Burkina Faso (urban, Jul 2010 - Jul 2011)[213]	II	NPA; DFA			14.0	
Rize, Turkey (urban, Jan 2014 - Jan 2015)[214]	II	NPS; RT-PCR	51.2	52.1	49.2	47.3
Aliasghar children's hospital, Iran (urban, Mar 2010 - Mar 2013)[215]	SARI	NPA; RT-PCR	33.7	34.7		31.0
Riyadh, Saudi Arabia (urban, Feb 2008 - Feb 2009)[216]	II	NPA; RT-PCR				22.4
Yaounde, Cameroon (urban, Sep 2011 - Sep 2013)[217]	II	NPS; RT-PCR	67.2	67.5		67.1
Vila Real, Portugal (urban, Sep 2005 - Dec 2015)[218]	II	NPA; IF			47.7	
Nicosia, Cyprus (urban, Nov 2010 - Oct 2013)[219]	II	NPS; RT-PCR	46.2		36.9	
Shenzhen Children's Hospital, China (urban, Jan 2012 - Dec 2015)[220]	II	NPS; DFA	13.4		11.7	
Lima, Peru (urban, Jan 2009 - Sep 2010)[221]	II	NPS; RT-PCR				17.3
Chonburi hospital, Thailand (urban, Jun 2013 - May 2014)[222]	II	NPA; RT-PCR				21.6
Baiyin, China (urban, Apr 2012 - Mar 2015)[223]	II	NPA; RT-PCR	37.6	36.2		33.0
Shijiazhuang, China (urban, Mar 2014 - Feb 2015)[224]	II	NPA; DFA	47.0	32.2	33.3	

Location (reference)	Case definition (codes)*	Specimen and diagnostic test*	Proportion of RSV associated hospitalised ALRI (%)			
			0-11m	0-23m	0-35m	0-59m
Guangzhou, China (urban, 2011 - 2013)[225]	II	NPA; RT-PCR	21.2	19.9		17.6
Wenzhou, China (urban, Mar 2013 - Feb 2014)[226]	II	NPA; DFA	25.7		19.0	
Jinhua, China (urban, Feb 2013 - Jan 2014)[227]	II	NPA; DFA	32.5		27.0	
Chongqing, China (urban, Jan 2014 - Dec 2014)[228]	II	NPA; IF	32.5		35.4	
Suzhou, China (urban, Jan 2013 - Dec 2014)[229]	II	NPA; DFA	24.9		23.9	
Shijiazhuang, China (urban, Mar 2014 - Feb 2015)[230]	II	NPA; DFA	35.1		29.9	
Cangzhou, China (urban, May 2012 - Jul 2013)[231]	II	NPA; DFA			26.3	
Donguan, China (urban, Jan 2013 - Dec 2014)[232]	II	NPA; RT-PCR		27.7		

Supplementary table 7: Description of 103 studies reporting proportion of in-hospital CFR for RSV associated ALRI in children younger than 5 years positive for RSV

Location (reference)	Case definition (coding)*	Specimen and diagnostic test*	In-hospital CFR of RSV associated hospitalised ALRI (%)					
			0-5m	6-11m	0-11m	0-23m	0-35m	0-59m
Hong Kong, China (urban, 1st Jan 2004 - 31st Dec 2004)[52]	II	NPA; DFA					0.0	
Taiwan, China (urban, Jan 2001 - Dec 2003)[54]	II	NPA; virus culture, IF						0.0
Ho Chi Minh, Vietnam (urban, Nov 2004 - Jan 2008)[63]	II, T+, CWI+/-, ICU+/-	nasal swab, throat swab, NPA; RT-PCR						0.0
Palma, Spain (urban, Jan 1995 - Dec 2006)[91]	II	NPA, NPW; ELISA, culture				0.1		
San Luis Potosi, Mexico (urban, 1st May 2003 - 30th Apr 2005)[114]	II	NPW; DFA					0.0	
Alaska, USA (rural, 4th Oct 2005 - 30th Sep 2007) [†233]	II	NPS, NPW; RT-PCR					0.0	
Dallas, Texas, USA (urban, Jan 2002 - Dec 2007)[117]	II	NPA; IF, DFA, virus culture				0.1		
Jingzhou, China (urban, Jan - Dec 2011)[234]	III, W+/-, T+/-, XR	NPS, OPS; RT-PCR						0.0
Taiwan, China (urban, 2004 - 2007)[235]	II	NA						0.1
Sa Kaeo and Nakhom Phanom, Thailand (rural, 1st Jan 2004 - 31st Dec 2007)[4]	II, XR	NPS, serum; RT-PCR, IFA						0.1
Damanhour, Egypt (urban and rural, Jun 2009 - Jun 2012)[10]	II	NPS, OPS; RT-PCR						0.0
Lørenskog, Norway (periurban, Feb 1993 - Jan 2000)[23]	II	NPA; ELISA				0.2		
272 institutions, Japan (urban, Aug 2006 - Jul 2008)[236]	II	NA					1.4	
Cairo, Egypt (urban, Feb 2010 - May 2011)[237]	III	NPS and OPS; RT-PCR			5.0			

[*] NPA=nasopharyngeal aspirate. NPS=nasopharyngeal swab. NPW=nasopharyngeal wash. OPS=oropharyngeal swab. RT-PCR=reverse transcriptase polymerase chain reaction. IFA=indirect immunofluorescent antibody test. DFA=direct immunofluorescent antibody test. ELISA=enzyme-linked immunosorbent assay. IF=immunofluorescence. II=physician's assessment. III=SARI definition. F+/-=fever as part of case definition or not. T+/-=tachypnea part of case definition or not. W+/-=wheeze part of case definition or not. C+/-=crepitation part of case definition or not. XR=X-ray confirmed (radiologically confirmed pneumonia). CWI+/-=chest wall indrawing part of case definition or not. DS+/-=danger signs part of case definition or not. O2+/-=hypoxemia part of case definition or not. ICU=intensive care unit. MV=mechanical ventilation.

[†] Reported data from aboriginal population in high income country

Location (reference)	Case definition (coding)[*]	Specimen and diagnostic test[*]	In-hospital CFR of RSV associated hospitalised ALRI (%)					
			0-5m	6-11m	0-11m	0-23m	0-35m	0-59m
Amman, Jordan (urban, 18th Jan - 29th Mar 2007)[238]	II, F+/-	nasal and throat swab; RT-PCR						0.9
Croatia (urban, Sep 2003 - Oct 2009)[239]	II	NPA; DFA						0.2
Beer-Sheva, Israel (urban, Nov to Mar, 2004 - 2011)[240]	II, CWI+, XR	NPW; DFA and culture				0.2		
Belgrade, Serbia (urban, Nov 2008 - Mar 2009)[241]	II	NPA; EIA			2.2			
Athens, Greece (urban, Feb 1997 - Jun 2000)[126]	II, XR, W+/-, T+/-	NPW; DFA			0.7			
Stockholm, Sweden (urban, 1987 - 1998)[30]	II	NPW; IFA			0.3			
Multicentric, Germany (urban, Nov 1999 - Oct 2001)[20]	II	NPS; PCR					0.7	
Shropshire, United Kingdom (urban, Apr 1996 - Mar 1999)[29]	II	NA[‡]; IF				0.2		
Berne, Switzerland (urban, Jul 1997 - Jun 2001)[242]	II	NPS; DFA						0.2
Freiburg, Germany (urban and rural, Apr 1997 - Mar 1999)[243]	II	NPS; IF						0.7
Germany (urban, 1999 - 2005)[244]	II	NPA; ELISA, culture, PCR			0.5			
Manhica, Mozambique (rural, Oct 1998 - May 2000)[125]	II, CWI+/-, W+/-, T+/-	NPA; ELISA						3.4
Banjul, Fajara and Sibanor, Gambia (urban and rural, Oct 1993 - Dec 1996)[33]	II	NPA; IF				2.0		
Hong Kong, China (urban, Jan 1993 - Dec 1997)[35]	II	NPA, throat swab; DFA, virus isolation						0.1
Sydney, Australia (urban, May 1997 - Oct 1999)[245]	II	NA				0.3		
Townsville, Australia (urban and rural, Jan 1997 - Oct 1999) [†36]	II	NA; IF, culture			1.1			
Navajo and WMA, USA (rural, Oct 1997 - Mar 2000) [†246]	II	NPA; ELISA				0.1		
Buenos Aires, Argentina (urban, Apr 1993 - Dec 1994)[247]	II	NPA; DFA						1.3
Santiago, Chile (urban, Jan 1989 - Dec 2000)[123]	II, W-	NPA; IFA				0.1		
Graz, Austria (urban, Nov 1999 - Oct 2000)[31]	II	NPA; ELISA			0.0			
Cape Town, South Africa (urban, Jun 1995 - Aug 1996)[124]	II, W+/-	NPA; ELISA				2.1		

[‡] NA= not available

Location (reference)	Case definition (coding)*	Specimen and diagnostic test*	In-hospital CFR of RSV associated hospitalised ALRI (%)					
			0-5m	6-11m	0-11m	0-23m	0-35m	0-59m
Buenos Aires, Argentina (urban, May 1991 - Dec 1992)[127]	II, CWI+/-, XR, W+/-, T+/-	NPA; IFA				0.0		
Sao Paulo, Brazil (urban and rural, Mar 1995 - Aug 1996)[248]	II	NPA, NPS; IFA						0.0
Tlaxcala, Mexico (urban and rural, Oct 1994 - Jun 1995)[249]	II	NPS; IFA						0.0
Alaska, USA (rural, Jul 2001 - Jun 2004) (Singleton and colleagues) †	II	NPS, NPW; RT-PCR					0.4	
Buenos Aires, Argentina (urban, 2001 - 2013) (Gentile and colleagues) §	II, MV+/-	NPA; IFA	1.9	1.7	1.8	1.8	1.8	1.8
Takeo ad Kampong Cham, Cambodia (urban, Apr 2007 - Feb 2010) (Goyet and colleagues) §	II, F+, T+	NPS, throat swab; RT-PCR	0.0	0.0	0.0	0.0	0.0	0.0
Beijing, China (urban, Jan 2011 - Dec 2012) (GABRIEL) §	II, T+, W-, XR	NPA, NPS; RT-PCR	0.0	0.0	0.0	0.0	0.0	0.0
Ulaanbaatar, Mongolia (urban, Sep 2011 - Oct 2012) (GABRIEL) §	II, T+, W-, XR	NPA, NPS; RT-PCR	0.0	0.0	0.0	0.0	0.0	0.0
Phnom Penh, Cambodia (urban, Oct 2010 - Jan 2013) (GABRIEL) §	II, T+, W-, XR	NPA, NPS; RT-PCR	0.0	0.0	0.0	0.0	0.0	0.0
Lucknow, India (urban and rural, Jun 2012 - Dec 2013) (GABRIEL) §	II, T+, W-, XR	NPA, NPS; RT-PCR	0.0	0.0	0.0	0.0	0.0	0.0
Antananarivo, Madagascar (urban and rural, Dec 2010 - Feb 2013) (GABRIEL) §	II, T+, W-, XR	NPA, NPS; RT-PCR	0.0	0.0	0.0	0.0	0.0	0.0
Asuncion, Paraguay (urban, Jul 2010 - May 2013) (GABRIEL) §	II, T+, W-, XR	NPA, NPS; RT-PCR	0.0	0.0	0.0	0.0	0.0	0.0
Bamako, Mali (urban, Jul 2011 - Nov 2012) (GABRIEL) §	II, T+, W-, XR	NPA, NPS; RT-PCR	0.0	14.3	4.2	3.6	3.3	3.2
Berlin, Germany (urban, 1st Apr 2010 - 31st Mar 2014) (Rath and colleagues) §	II, F+, ICU+/-	NPS, NPA; RT-PCR	0.0	0.0	0.0	0.0	0.0	0.0
Amman, Jordan (urban, Mar 2010 - Mar 2013) (Khuri and colleagues)	II, T+, O2+/ICU/MV	nasal and throat swab; PCR	0.4	0.4	0.4	0.4		
Lwak, Kenya (rural, 2007 - 2011) (Montgomery and colleagues) §	II, CWI+/DS+/O2+	NPS, OPS; RT-PCR	8.3	0.0	4.2	3.8	4.6	4.0
Rabat, Morocco (urban, Nov 2010 - Dec 2011) (Bassat and colleagues) §	II, CWI+, T+	NPA; RT-PCR	4.5	6.1	5.2	3.8	3.2	2.9
Karachi, Pakistan (urban, Aug 2009 - Jul 2012) (Ali and colleagues) §	II	throat swab; RT-PCR	1.1	0.0	0.7	0.5	0.5	0.9
Basse Santa Su, Gambia (rural, 3rd Nov 2011 - 2nd Nov 2013) (PERCH) §	II, CWI+, DS+/-	NPS, OPS; PCR	0.0	0.0	0.0	0.0	0.0	0.0
Kilifi, Kenya (urban, 15th Aug 2011 - 15th Nov 2013) (PERCH) §	II, CWI+, DS+/-	NPS, OPS; PCR	3.4	3.7	3.5	3.6	3.5	4.0

§ Reported in-hospital CFR separately for each of the following age bands- 0-5m, 6-11m and 12-59m

Location (reference)	Case definition (coding)*	Specimen and diagnostic test*	In-hospital CFR of RSV associated hospitalised ALRI (%)					
			0-5m	6-11m	0-11m	0-23m	0-35m	0-59m
Bamako, Mali (urban, 3rd Jan 2012 - 14th Jan 2014) (PERCH) §	II, CWI+, DS+/-	NPS, OPS; PCR	1.7	5.6	2.2	2.7	2.6	2.6
Nakhon Phanom and Sa Kaeo, Thailand (rural, 1st Jan 2012 - 31st Jan 2014) (PERCH) §	II, CWI+, DS+/-	NPS, OPS; PCR	0.0	0.0	0.0	0.0	0.0	0.0
Lusaka, Zambia (urban, 10th Oct 2011 - 31st Oct 2013) (PERCH) §	II, CWI+, DS+/-	NPS, OPS; PCR	4.8	8.0	5.7	6.1	5.9	5.8
Dhaka, Bangladesh (urban, 1st Jan 2012 - 31st Dec 2013) (PERCH) §	II, CWI+, DS+/-	NPS, OPS; PCR	0.0	0.0	0.0	0.0	0.0	0.0
Matlab, Bangladesh (rural, 1st Jan 2012 - 31st Dec 2013) (PERCH) §	II, CWI+, DS+/-	NPS, OPS; PCR	0.0	0.0	0.0	0.0	0.0	0.0
Tacloban City, Philippines (rural, May 2008 - Jul 2012) (Lupisan and colleagues) §	II, CWI+, DS+/-	NPS, serum; PCR, virus isolation, culture, serology	2.5	2.0	2.3	1.8	1.7	1.7
Mpumalanga, South Africa (rural, Jan 2010 - Dec 2014) (Cohen and colleagues) §	II	NPA; PCR	2.4	4.6	3.2	2.6	2.4	2.2
CEMIC, Buenos Aires, Argentina (urban, 1st Jun 2008 - 31st Dec 2010) (Echavarria and colleagues) §	II, ICU+/-, MV+/-	NPA; IFA	0.0	0.0	0.0	0.0	0.0	0.0
Buenos Aires, Argentina (urban and rural, 2011 - 2013) (Polack and colleagues)	II, O2+, W+, F+/-, ICU+/-, MV+/-	NPA; PCR	1.4	1.1	1.3	1.1		
Belo Horizonte, Brazil (urban, 2011 - 2013) (Oliveira and colleagues) §	II, F+, O2+/-, ICU+/-	nasal swab; RT-PCR	1.8	2.0	1.9	1.6	1.4	1.3
Concepcion, Chile (urban and rural, Jan 2012 - Dec 2013) (Fasce and colleagues) §	II, F+/-, ICU+/-	NPA; IF	0.0	0.0	0.0	0.0	0.0	0.5
Iquique, Chile (urban and rural, Jan 2012 - Dec 2013) (Fasce and colleagues) §	II, F+/-, ICU+/-	NPA; IF	0.0	3.3	1.0	0.9	0.9	0.8
Santa Ana, El Salvador (urban, 2008 - 2013) (Clara and colleagues)	III, T+/-, XR	NPS; IFA			23.3	17.6		15.9
Western Gambia (urban and rural, Jul 2007 - Jun 2008) (Howie and colleagues)	II, CWI+/-, W-, O2+/-	NPA; PCR		0.0	0.0	0.0	0.0	0.0
Santa Rosa, Guatemala (urban and rural, 2008 - 2013) (McCracken and colleagues) §	II, CWI+/-, DS+/-	NPS, OPS; RT-PCR	3.7	1.7	3.1	2.8	2.6	3.0
Quetzaltenango, Guatemala (urban and rural, 2009 - 2013) (McCracken and colleagues) §	II, CWI+/-, DS+/-	NPS, OPS; RT-PCR	0.9	1.4	1.0	1.4	1.3	1.3
Ballabhgarh, India (rural, 2010 - 2012) (Broor and colleagues) §	II, DS+/-	NPS; RT-PCR	0.0	0.0	0.0	0.0	0.0	0.0
Pune, India (rural, May 2009 - Apr 2013) (Chadha and colleagues) §	II	NPS; RT-PCR	0.0	0.0	0.0	0.0	0.0	0.0
Lombok, Indonesia (urban and rural, 2000 - 2002) (Gessner and	II, CWI+	nasal wash; ELISA	3.3	0.7	2.1	1.8		

Location (reference)	Case definition (coding)*	Specimen and diagnostic test*	In-hospital CFR of RSV associated hospitalised ALRI (%)					
			0-5m	6-11m	0-11m	0-23m	0-35m	0-59m
colleagues)								
Kilifi hospital study, Kenya (urban and rural, Jan 2002 - Dec 2010) (Nokes and colleagues) §	II, CWI+, O2+/DS+	OPS, NPS, nasal wash; DFA	2.1	2.2	2.1	1.9	2.2	2.2
Bondo district, Kenya (rural, Jan 2007 - Jun 2009) (Feikin and colleagues) §	II, O2+/DS+	NPS, OPS; RT-PCR	0.0	7.7	2.6	1.8	1.6	1.5
Manhiça, Mozambique (rural, 20th Sep 2006 - 19th Sep 2007) (Bassat and colleagues) §	II, T+	NPA; RT-PCR	0.0	10.0	2.9	2.3	2.2	2.0
Manhiça, Mozambique (rural, 1st Jan 2011 - 30th Jun 2014) (Bassat and colleagues) §	II, T+, O2+/-	NPA; RT-PCR	0.0	0.0	0.0	0.0	0.0	0.0
Utrecht, Netherlands (urban, 2001 - 2010) (Bont and colleagues)	II	NPS, NPW; PCR, IF	0.0	0.0	0.0			
41 sites in Netherlands (urban and rural, 2008 - 2013) (Bont and colleagues)	II, ICU+/-	NPS, NPW; PCR, IF	0.0	0.0	0.0			
David City, Panama (urban, Jan 2011 - Dec 2013) (Jara and colleagues) §	II	NPS, OPS; IFA, RT-PCR	0.0	0.0	0.0	0.0	0.0	0.0
Tagbilaran and 6 rural sites, Philippines (urban and rural, 5th Jul 2000 - 31st Dec 2004) (Lucero and colleagues)	II, CWI+, DS+/-	NPA, nasal swab; culture, PCR	0.8	0.8	0.8	0.6		
Gauteng province, South Africa (urban, 2009 - 2012) (Cohen and colleagues) §	II	NPA; PCR	0.9	0.0	0.6	0.5	0.5	0.5
KwaZulu-Natal province, South Africa (periurban, 2010 - 2014) (Cohen and colleagues) §	II	NPA; PCR	0.0	0.0	0.0	0.0	0.0	0.0
Klerksdorp site, South Africa (periurban, Jan 2011 - Dec 2014) (Cohen and colleagues) §	II	NPA; PCR	2.6	0.0	2.0	1.6	1.5	1.5
Soweto, South Africa (urban, Mar 1998 - Oct 2005) (Madhi and colleagues) §	II, CWI+, O2+/-	NPA; IF	2.4	1.0	2.0	1.7	1.7	1.6
Paarl, South Africa (periurban, Mar 2012 - Dec 2014) (Zar and colleagues)	II, CWI+, T+, DS+/-	NPS; RT-PCR	0.0	0.0	0.0	0.0		
Sa Kaeo and Nakhon Phanom, Thailand (rural, Jan 2008 - Dec 2011) (Thamthitiwat and colleagues) §	II, F+/-, T+/-, CWI+, O2+/-	NPS; PCR	0.0	0.0	0.0	0.0	0.0	0.0
Tone District, Togo (rural, Aug 2011 - Dec 2013) (Gessner and colleagues) §	II, CWI+	nasal wash; RT-PCR	0.0	0.0	0.0	0.0	0.0	0.0
Colorado, USA (urban and rural, Jan 2008 - Jun 2013) (Simoes and colleagues) §	II	nasal wash; PCR	0.0	0.1	0.0	0.0	0.1	0.1
Navajo and WMA, USA - MEDI (rural, Oct 2004 - Dec 2010) (O'Brien and colleagues) †	II, O2+/-	NPS; RT-PCR	0.0	0.0	0.0			

Location (reference)	Case definition (coding)*	Specimen and diagnostic test*	In-hospital CFR of RSV associated hospitalised ALRI (%)					
			0-5m	6-11m	0-11m	0-23m	0-35m	0-59m
Navajo and WMA, USA - EPI (rural, Oct 1997 - Mar 2000) (O'Brien and colleagues) †	II, O2+/-	NPA; EIA	0.3	0.0	0.2	0.1		
Nha Trang, Vietnam (urban and rural, Feb 2007 - Dec 2012) (Yoshida and colleagues) §	II, CWI+/DS+	NPS; RT-PCR	0.0	0.0	0.0	0.0	0.0	0.0
Spain (urban, Jan 1997 - Dec 2011)[5]	II	NA			0.1	0.1	0.1	0.1
Baguio, Philippines (urban, Apr 2009 - Dec 2011)[206]	III	NPS, OPS; RT-PCR				0.5		0.4
Tehran, Iran (urban, Jan 2012 - Dec 2012)[211]	II	NPA; RT-PCR						0.0
USA (urban, 2000, 2003, 2006 and 2009)[250]	II	NA				0.1		
USA (urban, 2000 - 2011)[250]	II	NA				0.3		
USA (urban, 2000)[9]	II	NA						0.1
San Luis Potosi, Mexico (urban, May 2003 - Dec 2014)[251]	II	NPA; NA	1.5	0.7	1.3	1.2	1.1	1.0
Seremban, Malaysia (urban, Jan 2008 - Dec 2013)[252]	II	NPA; DFA			1.4			1.6
Gothenburg, Sweden (urban, 2004 - 2011)[46]	II	NA; PCR, IF						0.0
National database, USA (urban, 2004 - 2013)[253]	II	NA				0.3		

Supplementary table 8: Description of 43 studies reporting proportion of in-hospital CFR (by narrow age bands) for RSV associated ALRI in children younger than 5 years positive for RSV

Location (reference)	Case definition (coding)*	Specimen and diagnostic test*	In-hospital CFR of RSV associated hospitalised ALRI (%)			
			0-5m	6-11m	12-23m	24-59m
Buenos Aires, Argentina (urban, 2001 - 2013) (Gentile and colleagues)	II, MV+/-	NPA; IFA	1.9	1.7	1.6	2.3
Takeo ad Kampong Cham, Cambodia (urban, Apr 2007 - Feb 2010) (Goyet and colleagues)	II, F+, T+	NPS, throat swab; RT-PCR	0.0	0.0	0.0	0.0
Beijing, China (urban, Jan 2011 - Dec 2012) (GABRIEL)	II, T+, W-, XR	NPA, NPS; RT-PCR	0.0	0.0	0.0	0.0
Ulaanbaatar, Mongolia (urban, Sep 2011 - Oct 2012) (GABRIEL)	II, T+, W-, XR	NPA, NPS; RT-PCR	0.0	0.0	0.0	0.0
Phnom Penh, Cambodia (urban, Oct 2010 - Jan 2013) (GABRIEL)	II, T+, W-, XR	NPA, NPS; RT-PCR	0.0	0.0	0.0	0.0
Lucknow, India (urban and rural, Jun 2012 - Dec 2013) (GABRIEL)	II, T+, W-, XR	NPA, NPS; RT-PCR	0.0	0.0		0.0
Antananarivo, Madagascar (urban and rural, Dec 2010 - Feb 2013) (GABRIEL)	II, T+, W-, XR	NPA, NPS; RT-PCR	0.0	0.0	0.0	0.0
Asuncion, Paraguay (urban, Jul 2010 - May 2013) (GABRIEL and colleagues)	II, T+, W-, XR	NPA, NPS; RT-PCR	0.0	0.0	0.0	0.0
Bamako, Mali (urban, Jul 2011 - Nov 2012) (GABRIEL)	II, T+, W-, XR	NPA, NPS; RT-PCR	0.0	14.3	0.0	0.0
Berlin, Germany (urban, 1st Apr 2010 - 31st Mar 2014) (Rath and colleagues)	II, F+, ICU+/-	NPS, NPA; RT-PCR	0.0	0.0	0.0	0.0
Lwak, Kenya (rural, 2007 - 2011) (Montgomery and colleagues)	II, CWI+/DS+/O2+	NPS, OPS; RT-PCR	8.3	0.0	3.3	4.3

* NPA=nasopharyngeal aspirate. NPS=nasopharyngeal swab. NPW=nasopharyngeal wash. OPS=oropharyngeal swab. RT-PCR=reverse transcriptase polymerase chain reaction. IFA=indirect immunofluorescent antibody test. DFA=direct immunofluorescent antibody test. ELISA=enzyme-linked immunosorbent assay. IF=immunofluorescence. II=physician's assessment. III=SARI definition. F+/-=fever as part of case definition or not. T+/-=tachypnea part of case definition or not. W+/-=wheeze part of case definition or not. C+/-=crepitation part of case definition or not. XR=X-ray confirmed (radiologically confirmed pneumonia). CWI+/-=chest wall indrawing part of case definition or not. DS+/-=danger signs part of case definition or not. O2+/-=hypoxemia part of case definition or not. ICU=intensive care unit. MV=mechanical ventilation.

Location (reference)	Case definition (coding)*	Specimen and diagnostic test*	In-hospital CFR of RSV associated hospitalised ALRI (%)			
			0-5m	6-11m	12-23m	24-59m
Rabat, Morocco (urban, Nov 2010 - Dec 2011) (Bassat and colleagues)	II, CWI+, T+	NPA; RT-PCR	4.5	6.1	0.0	0.0
Karachi, Pakistan (urban, Aug 2009 - Jul 2012) (Ali and colleagues)	II	throat swab; RT-PCR	1.1	0.0	0.0	3.7
Basse Santa Su, Gambia (rural, 3rd Nov 2011 - 2nd Nov 2013) (PERCH)	II, CWI+, DS+/-	NPS, OPS; PCR	0.0	0.0	0.0	0.0
Kilifi, Kenya (urban, 15th Aug 2011 - 15th Nov 2013) (PERCH)	II, CWI+, DS+/-	NPS, OPS; PCR	3.4	3.7	4.3	7.7
Bamako, Mali (urban, 3rd Jan 2012 - 14th Jan 2014) (PERCH)	II, CWI+, DS+/-	NPS, OPS; PCR	1.7	5.6	6.7	0.0
Nakhon Phanom and Sa Kaeo, Thailand (rural, 1st Jan 2012 - 31st Jan 2014) (PERCH)	II, CWI+, DS+/-	NPS, OPS; PCR	0.0	0.0	0.0	0.0
Lusaka, Zambia (urban, 10th Oct 2011 - 31st Oct 2013) (PERCH)	II, CWI+, DS+/-	NPS, OPS; PCR	4.8	8.0	9.1	0.0
Dhaka, Bangladesh (urban, 1st Jan 2012 - 31st Dec 2013) (PERCH)	II, CWI+, DS+/-	NPS, OPS; PCR	0.0	0.0	0.0	0.0
Matlab, Bangladesh (rural, 1st Jan 2012 - 31st Dec 2013) (PERCH)	II, CWI+, DS+/-	NPS, OPS; PCR	0.0	0.0	0.0	0.0
Tacloban City, Philippines (rural, May 2008 - Jul 2012) (Lupisan and colleagues)	II, CWI+, DS+/-	NPS, serum; PCR, virus isolation, culture, serology	2.5	2.0	0.0	0.0
Mpumalanga, South Africa (rural, Jan 2010 - Dec 2014) (Cohen and colleagues)	II	NPA; PCR	2.4	4.6	0.0	0.0
CEMIC, Buenos Aires, Argentina (urban, 1st Jun 2008 - 31st Dec 2010) (Echavarria and colleagues)	II, ICU+/-, MV+/-	NPA; IFA	0.0	0.0	0.0	0.0
Belo Horizonte, Brazil (urban, 2011 - 2013) (Oliveira and colleagues)	II, F+, O2+/-, ICU+/-	nasal swab; RT-PCR	1.8	2.0	0.0	0.0
Concepcion, Chile (urban and rural, Jan 2012 - Dec 2013) (Fasce and colleagues)	II, F+/-, ICU+/-	NPA; IF	0.0	0.0	0.0	6.2
Iquique, Chile (urban and rural, Jan 2012 - Dec 2013) (Fasce and colleagues)	II, F+/-, ICU+/-	NPA; IF	0.0	3.3	0.0	0.0
Santa Rosa, Guatemala (urban and rural, 2008 - 2013) (McCracken and colleagues)	II, CWI+/-, DS+/-	NPS, OPS; RT-PCR	3.7	1.7	1.2	5.7
Quetzaltenango, Guatemala (urban and rural, 2009 - 2013) (McCracken and colleagues)	II, CWI+/-, DS+/-	NPS, OPS; RT-PCR	0.9	1.4	2.8	0.0

Location (reference)	Case definition (coding)*	Specimen and diagnostic test*	In-hospital CFR of RSV associated hospitalised ALRI (%)			
			0-5m	6-11m	12-23m	24-59m
Ballabhgarh, India (rural, 2010 - 2012) (Broor and colleagues)	II, DS+/-	NPS; RT-PCR	0.0	0.0	0.0	0.0
Pune, India (rural, May 2009 - Apr 2013) (Chadha and colleagues)	II	NPS; RT-PCR	0.0	0.0	0.0	0.0
Kilifi hospital study, Kenya (urban and rural, Jan 2002 - Dec 2010) (Nokes and colleagues)	II, CWI+, O2+/DS+	OPS, NPS, nasal wash; DFA	2.1	2.2	0.5	5.8
Bondo district, Kenya (rural, Jan 2007 - Jun 2009) (Feikin and colleagues)	II, O2+/DS+	NPS, OPS; RT-PCR	0.0	7.7	0.0	0.0
Manhiça, Mozambique (rural, 20th Sep 2006 - 19th Sep 2007) (Bassat and colleagues)	II, T+	NPA; RT-PCR	0.0	10.0	0.0	0.0
Manhiça, Mozambique (rural, 1st Jan 2011 - 30th Jun 2014) (Bassat and colleagues)	II, T+, O2+/-	NPA; RT-PCR	0.0	0.0	0.0	0.0
David City, Panama (urban, Jan 2011 - Dec 2013) (Jara and colleagues)	II	NPS, OPS; IFA, RT-PCR	0.0	0.0	0.0	0.0
Gauteng province, South Africa (urban, 2009 - 2012) (Cohen and colleagues)	II	NPA; PCR	0.9	0.0	0.0	0.0
KwaZulu-Natal province, South Africa (periurban, 2010 - 2014) (Cohen and colleagues)	II	NPA; PCR	0.0	0.0	0.0	0.0
Klerksdorp site, South Africa (periurban, Jan 2011 - Dec 2014) (Cohen and colleagues)	II	NPA; PCR	2.6	0.0	0.0	0.0
Soweto, South Africa (urban, Mar 1998 - Oct 2005) (Madhi and colleagues)	II, CWI+, O2+/-	NPA; IF	2.4	1.0	0.7	0.0
Sa Kaeo and Nakhon Phanom, Thailand (rural, Jan 2008 - Dec 2011) (Thamthitiwat and colleagues)	II, F+/-, T+/-, CWI+, O2+/-	NPS; PCR	0.0	0.0	0.0	0.0
Tone District, Togo (rural, Aug 2011 - Dec 2013) (Gessner and colleagues)	II, CWI+	nasal wash; RT-PCR	0.0	0.0	0.0	0.0
Colorado, USA (urban and rural, Jan 2008 - Jun 2013) (Simoes and colleagues)	II	nasal wash; PCR	0.0	0.1	0.0	0.2
Nha Trang, Vietnam (urban and rural, Feb 2007 - Dec 2012) (Yoshida and colleagues)	II, CWI+/DS+	NPS; RT-PCR	0.0	0.0	0.0	0.0

Supplementary table 9: Regional meta-estimates of incidence of RSV-associated ALRI (by narrow age bands) in children younger than 5 years

Region	0-27days		28-<3m		3-5m		6-11m		12-23m		24-59m	
	No.*	Incidence	No.	Incidence	No.	Incidence	No.	Incidence	No.	Incidence	No.	Incidence
**Low income**	0		0		0		0		0		0	
**Lower middle income**	2	14.4 (0.3-609.1)	4	23.6 (7.6-72.9)	6	72.2 (48.8-106.7)	8	80.7 (48-135.6)	8	66.5 (39.4-112.2)	4	13.4 (8.5-20.9)
**Upper middle income**	1	235.3 (88.3-626.9)	1	471.4 (335.1-663.1)	1	619 (452.3-847.3)	2	223 (95.2-522.1)	2	155.7 (41.3-586.7)	0	
**High income**	0		0		0		0		0		0	
**Developing**	3	40 (2.5-635.7)	5	45.7 (5.9-356.1)	7	99.6 (38.5-257.7)	10	98.8 (58.8-166.1)	10	79.1 (45.1-138.9)	4	13.4 (8.5-20.9)
**Industrialised**	0		0		0		0		0		0	

* No. = Number of studies

Supplementary table 10: Meta-estimates of incidence of RSV-associated severe and very severe ALRI (by narrow age bands) in children younger than 5 years residing in developing countries

	0-27days		28-<3m		3-5m		6-11m		12-23m		24-59m	
	No.*	Incidence	No.	Incidence	No.	Incidence	No.	Incidence	No.	Incidence	No.	Incidence
**RSV-associated severe ALRI**	2	93.4 (11.2-778.3)	3	28.5 (1.4-594.7)	5	38.7 (5.7-265)	7	24.7 (11.5-53.2)	7	10.3 (4.6-23)	2	2.8 (1.3-6.2)
**RSV-associated very severe ALRI**	0	..	2	5.7 (0.2-155.4)	3	11.2 (1.7-75.2)	3	3.8 (0.5-29.6)	3	6.6 (2.5-17.5)	0	..

* No. = Number of studies

Supplementary table 11: Regional meta-estimates of hospitalisation rate of RSV-associated ALRI (by narrow age bands) in children younger than 5 years

Region	0-27 days		28-<3m		3-5m		6-8m		9-11m		12-23m		24-59m	
	No.*	Hospitalisation rate	No.	Hospitalisation rate	No.	Hospitalisation rate	No.	Hospitalisation rate	No.	Hospitalisation rate	No.	Hospitalisation rate	No.	Hospitalisation rate
**Low income**	2	5.0 (0.4-69.8)	3	5.2 (1.4-19.5)	2	6.8 (1.7-28.1)	3	2.5 (0.5-12.9)	2	1.8 (0.9-3.7)	4	1.3 (0.4-5.0)	4	0.4 (0.1-1.2)
**Lower middle income**	5	9.3 (2.5-35.3)	7	22.9 (13.3-39.5)	7	20.5 (11.9-35.4)	7	14.0 (7.8-25.4)	7	9.4 (4.1-21.7)	12	5.6 (3.3-9.7)	9	1.1 (0.6-1.9)
**Upper middle income**	3	46.4 (3.9-549.5)	4	74.5 (28.2-197)	5	25.7 (11.6-56.7)	5	16.9 (9.1-31.5)	5	18.8 (9.6-36.9)	8	7.1 (4.6-10.7)	6	1.4 (0.8-2.7)
**High income**	2	31.9 (26.9-37.8)	2	67.4 (45.3-100.5)	3	36.7 (19.1-70.6)	1	40.8 (36.9-45.1)	1	25.9 (22.9-29.4)	14	4.2 (3.4-5.4)	11	0.9 (0.6-1.2)
**Developing**	11	15.9 (8.8-28.9)	15	26.1 (16.7-40.7)	15	20.7 (12.9-33.0)	16	12.0 (7.9-18.3)	15	11.3 (7.3-17.4)	28	5.0 (3.8-6.4)	23	1.0 (0.7-1.4)
**Industrialised**	1	31.0 (22.0-43.6)	1	54.6 (45.5-65.5)	2	27.5 (23.4-32.4)	0		0		10	3.9 (3.0-5.3)	7	0.8 (0.6-1.2)

* No. = Number of studies

Supplementary table 12: Regional meta-estimates of proportion of hospitalised RSV-associated ALRI cases with hypoxemia in children younger than 5 years

Region	0-11m		12-23m		0-23m		24-59m		0-59m	
	No. of studies	Proportion (%)	No. of studies	Proportion (%)	No. of studies	Proportion (%)	No. of studies	Proportion (%)	No. of studies	Proportion (%)
**Low income**	8	16.4 (9.1-29.6)	8	18.9 (2.2-162.7)	8	14.4 (7.8-26.5)	8	10.7 (6.1-18.8)	8	13.9 (7.7-24.9)
**Lower middle income**	6	16.5 (9.1-30.0)	6	17.7 (9.9-31.8)	6	16.3 (9.2-29.1)	4	14.7 (6.5-33.3)	5	17.3 (7.8-38.6)
**Upper middle income**	9	26.9 (18.3-39.5)	9	23.0 (14.9-35.3)	9	24.3 (16.7-35.5)	5	36.6 (23.7-56.5)	5	30.5 (20.1-46.1)
**High income**	2	32.4 (8.3-127.3)	2	53.8 (28.1-103.0)	2	37.0 (11.7-117.4)	2	53.2 (29.9-94.9)	2	39.6 (14.2-110.5)
**Developing**	23	20.2 (15.1-26.9)	23	19.7 (12.8-30.4)	23	18.6 (14.0-24.8)	17	20.6 (13.3-31.9)	18	18.4 (12.8-26.4)
**Industrialised**	2	32.4 (8.3-127.3)	2	53.8 (28.1-103.0)	2	37.0 (11.7-117.4)	2	53.2 (29.9-94.9)	2	39.6 (14.2-110.5)

Supplementary table 13: Regional meta-estimates of proportion of hospitalised ALRI cases positive for RSV by World Bank income regions in children younger than 5 years

Region	0-11m		0-59m	
	No. of studies	Proportion (%)	No. of studies	Proportion (%)
**Low income**	8	21.1 (15.2-29.3)	6	20.9 (17.9-24.5)
**Lower middle income**	14	29.6 (23.4-37.4)	18	21.9 (18.1-26.5)
**Upper middle income**	103	28.7 (26.5-31.1)	53	23.0 (20.5-25.7)
**High income**	22	39.7 (34.7-45.4)	27	28.3 (25.3-31.6)
**Developing**	133	28.7 (26.7-30.8)	93	23.2 (21.4-25.3)
**Industrialised**	14	42.5 (35.7-50.6)	11	29.9 (24.1-37.1)

Supplementary table 14: Regional meta-estimates of proportion of hospitalised ALRI cases positive for RSV, and estimated hospitalisations by WHO regions in children younger than 5 years

Region	0-11m		0-59m		
	No. of studies	Proportion (%)	No. of studies	Proportion (%)	Hospitalised cases (*10^3)
**Africa**	10	27.3 (19.9-37.5)	13	22.7 (17.9-28.8)	1008 (795-1278)
**Americas**	19	37.5 (32.6-43.3)	15	27.6 (23.9-32.0)	170 (147-197)
**Eastern Mediterranean**	8	29.4 (21.1-41.1)	10	24.4 (18.9-31.4)	438 (340-564)
**Europe**	12	43.1 (35.4-52.6)	10	29.8 (23.8-37.4)	557 (444-699)
**South-East Asia**	10	30.8 (21.8-43.7)	9	23.9 (19.2-29.8)	629 (505-784)
**Western Pacific**	88	27.0 (24.8-29.5)	47	22.2 (19.8-24.9)	109 (97-122)
**Global**					**2911 (2329-3644)**

Supplementary table 15: Meta-estimate of hospitalisation rate (per 1000 children per year) and in-hospital CFR (%) for RSV-associated ALRI in children younger than 5 years in developing countries by subtypes

	0-5m		0-11m		0-59m			
	**No. of studies**	**Hospitalisation rate**	**No. of studies**	**Hospitalisation rate**	**No. of studies**	**Hospitalisation rate**	**No. of studies**	**hCFR**
RSV-A	5	9.2 (4.9-17.3)	5	9.0 (5.4-14.9)	6	2.4 (1.6-3.6)	9	2.3 (1.7-3.1)
RSV-B	3	2.1 (0.3-15.3)	4	2.5 (0.9-7.0)	4	0.7 (0.3-1.6)	7	1.5 (0.8-2.8)

Supplementary table 16: Proportion of eligible ALRI cases not tested for RSV (in the studies included in the analysis) with reasons thereof

Location (study period) (reference)	% of eligible cases not tested for RSV	Reasons for not testing
**Dhaka, Bangladesh (Jan 2005 - Feb 2008) (Brooks, unpublished)**	19.3 (290/1500)	NA
**Bandung, Indonesia (1st Feb 1999 - 26th Apr 2001) (Simoes, unpublished)**	65.6 (1535/2339)	subjects did not meet case definition (LRI)
**Gilgit, Pakistan (1st Apr 2012 - 31st Mar 2014) (Rasmussen, unpublished)**	14.3 (34/238)	child absent when collecting samples, samples were not valid by the time it reached the testing center, mother refusal
**Paarl, South Africa (Mar 2012 - Dec 2014) (Zar, unpublished)**	36.1 (116/321)	specimen not collected, PCR results not available
**Sa Kaeo and Nakhom Phanom, Thailand (1st Jan 2004 - 31st Dec 2007)[4]**	51.5 (11760/22857)	NA
**Damanhour, Egypt (Jun 2009 - Jun 2012)[10]**	12.4 (659/5324)	no informed consent or samples
**Multicentric, Germany (Nov 1999 - Oct 2001)[20]**	36.9 (1078/2924)	no informed consent, unavailable clinical specimens or specimens were not tested
**Banjul, Fajara and Sibanor, Gambia (Jan 1994 - Dec 1996)[33]**	59.5 (3305/5557)	no village of orgin documented, from outside the western region, no sex documented, samples not being taken because children admitted during weekends/holidays, absence of the patient from the ward for investigations and lack of documentation of an admission diagnosis on the patient's chart
**Nashville, Rochester and Cincinnati, USA (Oct 2000 - Sep 2004)[6]**	18.6 (1158/6225)	a lack of parental consent or unavailability of parents
**CEMIC, Buenos Aires, Argentina (1st Jun 2008 - 31st Dec 2010) (Echavarria, unpublished)**	5.3 (3/57)	insufficient sample
**Buenos Aires, Argentina (2011 - 2013) (Polack, unpublished)**	0.9 (30/3371)	sample inadequate
**Belo Horizonte, Brazil (2011 - 2013) (Oliveira, unpublished)**	59.4 (757/1275)	frozen samples at weekends; laboratory refused
**Concepcion, Chile (Jan 2012 - Dec 2013) (Fasce, unpublished)**	3.3 (16/481)	cases were not notified in time so as to collect sample
**Iquique, Chile (Jan 2012 - Dec 2013) (Fasce, unpublished)**	0.6 (4/683)	cases were not notified in time so as to collect sample
**Jingzhou, China (Jan 2010 - Dec 2012) (Yu, unpublished)**	97.1 (19092/19660)	sample selection
**Santa Ana, El Salvador (2008 - 2013) (Clara, unpublished)**	78.8 (4388/5570)	NA

Location (study period) (reference)	% of eligible cases not tested for RSV	Reasons for not testing
**Gambia Western Region, Gambia (Jul 2007 - Jun 2008) (Howie, unpublished)**	4.2 (4/95)	technical problem with specimen
**Santa Rosa, Guatemala (2008 - 2013) (McCracken, unpublished)**	5.7 (104/1810)	no consent or no specimen
**Quetzaltenango, Guatemala (2009 - 2013) (McCracken, unpublished)**	10.9 (207/1893)	no consent or no specimen
**Pune, India (May 2009 - Apr 2013) (Chadha, unpublished)**	12.7 (78/612)	poor quality swab; inadequate specimen; sample lost
**Lombok, Indonesia (2000 - 2002) (Gessner, unpublished)**	28.9 (1500/5198)	primarily age <3 months and critical illness on presentation; specimens collected 4 days a week so if patients who had rapid admission, would not have been done
**Kilifi hospital study, Kenya (Jan 2002 - Dec 2010) (Nokes, unpublished)**	24.4 (2817/11544)	died; discharged; refusal
**Bondo district, Kenya (Jan 2007 - Jun 2009) (Feikin, unpublished)**	28.7 (208/726)	low swab quality
**Manhiça, Mozambique (20th Sep 2006 - 19th Sep 2007) (Bassat, unpublished)**	3.4 (28/835)	NPA not collected or processed without results
**Manhiça, Mozambique (1st Jan 2011 - 30th Jun 2014) (Bassat, unpublished)**	3.1 (15/491)	NPA not collected or processed without results
**41 sites in Netherlands (2008 - 2013) (Bont, unpublished)**	10.0 (24/241)	reasons for not testing are unknown, as the decisions for testing are made by independent physicians at hospital of presentation
**David City, Panama (Jan 2011 - Dec 2013) (Jara, unpublished)**	81.8 (2152/2631)	lack of resources at the lab to test
**Tagbilaran and 6 rural sites, Philippines (5th Jul 2000 - 31st Dec 2004) (Lucero, unpublished)**	36.0 (537/1491)	no specimen collected for those cases not tested for RSV
**Gauteng province, South Africa (2009 - 2012) (Cohen, unpublished)**	2.0 (122/6016)	child too ill to take sample from; specimens not reaching laboratory within 72 hours
**KwaZulu-Natal province, South Africa (2010 - 2014) (Cohen, unpublished)**	4.5 (72/1607)	patient very ill; parents refuse consent, specimen not at lab within 72 hours
**Klerksdorp site, South Africa (Jan 2011 - Dec 2014) (Cohen, unpublished)**	3.2 (30/932)	patient very ill; parents refuse consent, specimen not at lab within 72 hours
**Soweto, South Africa (Mar 1998 - Oct 2005) (Madhi, unpublished)**	4.6 (240/5231)	missed as part of surveillance
**Maela Camp, Tak Province, Thailand (Nov 2007 - Oct 2010)**	2.3 (8/354)	parents refused NPA taken

Location (study period) (reference)	% of eligible cases not tested for RSV	Reasons for not testing
**(Turner, unpublished)**		
**Sa Kaeo and Nakhon Phanom, Thailand (Jan 2008 - Dec 2011) (Thamthitiwat, unpublished)**	75.0 (14334/19103)	NA
**Tone district, Togo (Aug 2011 - Dec 2013) (Gessner, unpublished)**	3.2 (4/124)	testing just started
**Alaska, USA (Jul 1994 - Jun 2012) (Singleton, unpublished)**	20.5 (1222/5965)	at provider discretion
**Navajo and WMA, USA - MEDI (Oct 2004 - Dec 2010) (O'Brien, unpublished)**	3.8 (5/132)	specimen not collected, most probably due to short hospitalization stay (<2 days)
**Nha Trang, Vietnam (Feb 2007 - Dec 2012) (Yoshida, unpublished)**	3.1 (25/795)	some sample being lost
**Subiaco, Australia (Jan 2000 - Dec 2005)[47]**	54.8 (10877/19849)	NA
**Lanzhou, China (Oct 2004 - Oct 2005)[50]**	7.1 (63/884)	lack of consent and convalescent serum sample was not obtained
**Hong Kong, China (Nov 2005 - Oct 2006)[53]**	80	sampling
**Nagasaki, Japan (Apr 2009 - Mar 2010)[58]**	3.0 (13/433)	NA
**Seoul, Korea (Jan - Dec 2004)[59]**	71.8 (509/709)	excluded during sampling process and small quantities not enough for RNA extraction
**Khon Kaen, Thailand (Apr 2002 - Aug 2004)[70]**	35.0 (91/260)	parents didn't give consent for collection of NPA from their children; inappropriately stored for viral detection
**Tak Province, Thailand (Apr 2009 - Sep 2011)[71]**	14.0 (117/836)	failure to meet the case definition; presented twice within 14 days; inadequate specimens
**Turku, Finland (Jan 1993 - Dec 1995)[80]**	14.2 (42/296)	a convalescent serum sample was not obtained, chest radiographs did not have infiltrates compatible with pneumonia at the reevaluation done by 3 pediatric radiologists, or the radiographs were not available for the reevaluation
**Kiel, Germany (Jul 1996 - Jun 2001)[82]**	37.3 (1015/2721)	NA
**Pisa, Italy (Jan 2000 - May 2006)[87]**	19.5 (39/200)	some specimens were frozen immediately after collection and unsuitable for virus detection by DFA or rapid culture in shell vials
**Milan, Italy (1st Dec 2008 - 31st Dec 2009)[89]**	4.0 (24/600)	no guardian consent
**Palma, Spain (Jan 1995 - Dec 2006)[91]**	17.5 (505/2889)	no info about RSV microbiology; erroneously codified; >2y; had a previous episode of obstructive lower airway infection

Location (study period) (reference)	% of eligible cases not tested for RSV	Reasons for not testing
**Madrid, Spain (Sep 2005 - Aug 2008)[93]**	14.1 (52/370)	lack of NPA samples or they refused to participate
**Lausanne and Geneva, Switzerland (Mar 2003 - Dec 2005)[95]**	10.8 (12/111)	impossibility to obtain samples or interrupt the study
**Pelotas, Brazil (1st Aug 1997 - 31st Jul 1998)[99]**	30.4 (266/874)	refusals and losses; insufficient available data to generate adequate diagnostic by the judge or the judge did not give a diagnostic of ARD; readmission; not tested for RSV
**Sao Paulo, Brazil (Jan 2006 - Dec 2007)[103]**	17.7 (53/299)	didn't meet criteria; incomplete data in their medical records
**Sao Paulo, Brazil (May 2004 - Sep 2005)[105]**	4.3 (13/303)	loss of specimen
**Sao Paulo, Brazil (Mar 2008 - Aug 2011)[107]**	27.5 (308/1121)	repeated or inadequate samples
**Belem, Brazil (Nov 2006 - Oct 2007)[108]**	13.5 (164/1214)	no consent from parents or legal guardians
**Porto Alegre, Brazil (Sep 2009 - Sep 2010)[109]**	3.0 (2/67)	no samples collected
**Iqaluit, Canada (28th Jan 2002 - 27th Mar 2003)[110]**	7.3 (9/124)	NA
**San Luis Potosi, Mexico (1st May 2003 - 30th Apr 2005)[114]**	16.1 (118/734)	no respiratory samples
**California, USA (1996 - 2004)[116]**	15.0 (309/2060)	NA
**Dallas, Texas, USA (Jan 2002 - Dec 2007)[117]**	4.4 (211/4800)	not tested
**Rarieda, Kenya (1st Mar 2007 - 28th Feb 2010)[120]**	78.1 (1250/1600)	NA
**Kilifi, Kenya (Jan - Dec 2010)[121]**	16.0 (154/963)	refused; died during the admission process; failure of the computerized flagging system; other reasons
**Bondo district, Kenya (Aug 2008 - Dec 2010)[122]**	58.9 (974/1654)	not tested
**Santiago, Chile (Jan 1989 - Dec 2000)[123]**	NA	poor sample quality or quantity
**Cape Town, South Africa (Jun 1995 - Aug 1996)[124]**	68.3 (2776/4064)	NA
**Manhica District, Mozambique (Oct 1998 - May 2000)[125]**	44.2 (793/1794)	no NPA or complete information or valid NPA result
**Athens, Greece (Feb 1997 - Jun 2000)[126]**	25.6 (163/636)	NA
**Buenos Aires, Argentina (2001 - 2013) (Gentile, unpublished)**	12.9 (1361/10581)	NA

Location (study period) (reference)	% of eligible cases not tested for RSV	Reasons for not testing
**Lwak, Kenya (2007 - 2011) (Montgomery, unpublished)**	72.4 (1231/1701)	refusal to consent; unavailability of transport from field site to lab; high patient volumes; inadequate staffing levels
**Kathmandu, Nepal (Jan 2006 - Jun 2008) (Basnet, unpublished)**	71.8 (1630/2271)	other severe illness, history of documented TB, severe malnutrition, heart disease, diarrhoea with dehydration, disappearance of indrawing after salbutamol, recurrent wheezing, effusion/pneumothorax on chest X-ray, Hb% < 7 gm/dl, chronic cough, already enrolled in study once
**Basse, Gambia (3rd Nov 2011 - 2nd Nov 2013) (PERCH, unpublished)**	7.2 (46/638)	quality control not yet complete
**Kilifi, Kenya (15th Aug 2011 - 15th Nov 2013) (PERCH, unpublished)**	0.3 (2/634)	specimen not collected
**Bamako, Mali (3rd Jan 2012 - 14th Jan 2014) (PERCH, unpublished)**	9.1 (61/674)	quality control not yet complete
**Nakhon Phanom and Sa Kaeo, Thailand (1st Jan 2012 - 31st Jan 2014) (PERCH, unpublished)**	18.3 (41/224)	quality control not yet complete
**Lusaka, Zambia (10th Oct 2011 - 31st Oct 2013) (PERCH, unpublished)**	10.5 (65/617)	quality control not yet complete
**Mpumalanga, South Africa (Jan 2010 - Dec 2014) (Cohen, unpublished)**	4.0 (52/1310)	patient very ill; parents refuse consent; specimen not at lab within 72 hours
**Beirut, Lebanon (Oct 2012 - Mar 2014)[209]**	18.3 (99/542)	no specimens collected or had onset of respiratory symptoms for more than 7 days
**Buenos Aires, Argentina (Apr 1993 - Dec 1994)[247]**	3.4 (44/1278)	NA
**Beer-Sheva, Israel (Nov to Mar, 2004 - 2011)[240]**	52.8 (1182/2240)	specimens were not obtained
**Jingzhou, China (Jan - Dec 2011)[234]**	93.0 (6803/7314)	random samples
**Amman, Jordan (18th Jan - 29th Mar 2007)[238]**	2.0 (15/743)	clinical or laboratory data were not available

Supplementary table 17: Method for calculating country specific estimates for RSV-associated ALRI

Step 1	We conducted a systematic literature review to identify the risk factors for RSV in children aged 0-4 years (Shi et al., 2015). We selected risk factors which in meta-analyses had strong statistically significant associations with RSV. These were:<br>• Prematurity (gestational age <37 weeks) - OR 1.96 (1.44-2.67)<br>• Low birth weight (<2500 gms) - OR 1.91 (1.45-2.53)<br>• Siblings - OR 1.6 (1.32-1.95)<br>• Maternal smoking - OR 1.36 (1.24-1.5)<br>• Paediatric HIV - OR 3.74 (2.47-5.66)<br>• Crowding - OR 1.94 (1.29-2.93)
Step 2	We obtained data on the prevalence of low birth weight, siblings and crowding from nationally representative household surveys with large sample sizes - the Demographic and Health surveys (DHS). DHS surveys are generally carried out every 2 to 5 years and provide data on demography and health indicators. The data for China were obtained from the Chinese National Bureau of Statistics. The HIV data were obtained from the United Nations programme on HIV/AIDS (UNAIDS) estimates for 2013 (http://www.unaids.org/en/media/unaids/contentassets/documents/epidemiology/2013/gr2013/20131118_Methodology.pdf). Maternal smoking data were obtained from previously published global estimates (Ng et al JAMA 2013) and prematurity data were obtained from WHO estimates (Blenclowe et al, Lancet 2012). We ensured that the definitions of risk factors were the same in the studies estimating the risk ratios as in the surveys that measured prevalence of exposure to these risk factors.<br>Specifically, we defined each risk factor using one or more DHS variables. Then, using individual patient data from the survey for each country, we calculated the proportion of participants with each combination of risk factors.<br>We chose the standard DHS survey (i.e. not special surveys) with these risk factors recorded closest to 2015 respectively.
Step 3	We categorised the developing countries into different subgroups based on the six WHO regions and child and adult mortality strata: A, very low child and very low adult mortality; B, low child and low adult mortality; C, low child and high adult mortality; D, high child and high adult mortality; E, high child and very high adult mortality. Where available, we decided to use the latest data for the prevalence of the six risk factors. If the prevalence data were not available for any risk factor in a particular country, we imputed the median

prevalence for the subgroup to which the country belonged.

Step 4 The total rates for RSV-associated ALRI in developing countries were obtained from meta-analyses described in the methods section of the paper. The estimates and standard errors for each meta-estimate was used to define log-normal distributions. In R, we obtained 10,000 from these distributions.

The rate of RSV-associated ALRI in a population is the sum of each stratum specific rate, weighted by the proportion in each stratum where there are *j* mutually exclusive strata: -

$$Total\ rate = \frac{\sum x_j}{\sum n_j} = \sum_j \left(\frac{x_j}{n_j} \cdot \frac{n_0}{x_0} \cdot \frac{x_0}{n_0} \cdot \frac{n_j}{\sum n_j} \right)$$

where x is the number of cases in a stratum, n is the total population in a stratum and where j = 0 indicates the stratum which has no exposures (i.e. the unexposed) and j = 1 to j = 127 indicate the strata for each unique combination of risk factors. This equation can be re-written as: -

$$Total\ rate = \frac{x_0}{n_0} \times \sum j \left(\frac{x_j}{n_j} \cdot \frac{n_0}{x_0} \cdot \frac{n_j}{\sum n_j} \right)$$

where $\frac{x_0}{n_0}$ is the rate in the unexposed, $\frac{x_j}{n_j} \cdot \frac{n_0}{x_0}$ is the rate ratio, and $\frac{n_j}{\sum n_j}$ is the proportion in each stratum.

Therefore, using the regional estimates (for developing countries) of the total rate, the combined rate ratio for each stratum, and the proportion in each stratum calculated the incidence in unexposed people for the region (all developing countries). This was estimated to be 26.6 (15.6-44.3) episodes per 1000 children aged below 5 years per year. If we then assume that the rate in the unexposed is consistent across the region (all developing countries) we can then calculate the incidence rate for each country: -

$$Country\ specific\ rate = \frac{\sum x_j^c}{\sum n_j^c} = \frac{\sum x_j^r}{\sum n_j^r} \cdot \frac{\sum_j \left(\frac{x_j}{n_j} \cdot \frac{n_0}{x_0} \cdot \frac{n_j^r}{\sum n_j^r} \right)}{\sum_j \left(\frac{x_j}{n_j} \cdot \frac{n_0}{x_0} \cdot \frac{n_j^c}{\sum n_j^c} \right)}$$

where c indicates a country specific value and r indicates a regional value.

Step 5 Assumptions:

- As with attributable fraction calculations, we assume that the pooled risk ratios obtained from a range of case-control/cohort studies in the developing region were applicable across all developing countries. As described in Step 6 we added additional uncertainty to our estimates to somewhat relax this assumption.
- As in our previous analysis (Theodoratou et al., 2014) we assumed that there was an n-way interaction between each risk factor (1.25 fold reduction) such that rate ratios combined sub-multiplicatively (e.g. for the group with malnutrition and low birth weight, RR_A = 4.5, and RR_B = 3.6, $RR_{A\&B}$ = 4.5 x 3.6 / 1.25 = 12.96 rather than 4.5 x 3.6 =16.2). None of the primary studies included in the meta-analysis reported interaction/heterogeneity of effect measure estimates on a multiplicative scale. However, we noted that the majority of studies did not report testing for interaction and it seemed unlikely to us that all risks combined fully multiplicatively. Therefore, we added a weak interaction term to shrink the combined rate ratios towards the null. This would have the effect of causing the country specific estimates to be attenuated towards the overall estimate for the developing region.
- We also obtained individual-level patient data from two collaborating sites and estimated all two-way interactions for prematurity, siblings, overcrowding and low birth weight except low birth weight-siblings (as no patients had this combination). There were 92 cases with RSV of 311 patients. As expected the confidence intervals were very wide, offering little empirical guidance to the choice of a specified interaction odds ratio.

Interaction term	Odds ratio	Lower CI	Upper CI
**prematurity:siblings**	2.66	0.2	35.89
**prematurity:crowding**	0.67	0.04	10.15
**prematurity:lbw**	0.83	0.27	2.61
**lbw:crowding**	6.71	0.72	62.79
**siblings:crowding**	2.68	0.2	35.36

This justifies our choice of adding a weak interaction term (as described above).

- In order to calculate the proportion of the population in each country within each of the j strata we first aggregated the survey data to obtain a marginal (overall) estimate for each risk factor, then assumed independence to estimate the proportion of individuals with each combination of risk factors (i.e. the proportion in each of the j strata).

This assumption was made so that the uncertainty around the proportion for each combination of risk factors could be obtained by sampling from 6 beta distributions (with shape parameters determined by the number of participants in the survey with and without each of the 6 risk factors), then multiplying together each of the 6 sampling distributions. We could instead have used the actual number of participants with each combination of risk factors, and sampled instead from a Dirlecht distribution (a multivariate extension of the beta distribution). However, our chosen approach had a number of advantages. Firstly, it allowed us to use data from more than one source (only 3 of the 6 risk factors were well recorded in the DHS surveys). Secondly, data processing was more straightforward. Thirdly, it meant that we could make the data used in the analyses publicly available, which would not have been possible had we used the actual joint proportions, which are potentially disclosive.

- Finally we assumed that the rate among the unexposed (i.e. the risk in the absence of these SIX risk factors) came from the same distribution for all countries. This is the same as assuming that there was no residual confounding.

Step 6 Using a simulation based approach, we performed the calculation described in step 4 to estimate the country specific rates along with uncertainty ranges (UR).

This was done by performing this calculation for each of the 10,000 samples from the distributions representing the incidence rates, odds ratios and risk factor proportions (methods used to obtain each of these were obtained as described above) within R. Full analysis code and data are available from the authors on request.

Supplementary table 18: Incidence and number of episodes of RSV-associated ALRI in 132 developing countries

Country	Incidence of RSV-associated ALRI (per 1000 children per year)	No. of episodes of RSV-associated ALRI (UR)
**Afghanistan**	53.3 (33.6-83.8)	263677 (166462-414858)
**Algeria**	51.6 (32.7-80.9)	236857 (150049-371261)
**Angola**	53.7 (34-84.1)	253440 (160269-397001)
**Antigua and Barbuda**	46.8 (29.7-73.4)	341 (216-534)
**Argentina**	48.3 (30.7-75.8)	179694 (114110-281976)
**Bahamas**	48.2 (30.5-75.4)	1402 (889-2195)
**Bahrain**	57.2 (36-89.9)	6217 (3918-9773)
**Bangladesh**	51.9 (32.9-81.2)	796123 (504325-1245371)
**Barbados**	47.7 (30.2-74.8)	829 (525-1299)
**Belize**	53 (33.6-83)	2092 (1324-3275)
**Benin**	57.4 (36.1-90.5)	98087 (61677-154467)
**Bhutan**	39.6 (24.3-64.3)	2611 (1600-4239)
**Bolivia**	50.2 (31.7-78.7)	59473 (37562-93249)
**Botswana**	60 (37.6-94.2)	15976 (10011-25088)
**Brazil**	51.4 (32.5-80.3)	773070 (488993-1207125)
**Burkina Faso**	59.7 (37.5-93.9)	187717 (117797-295328)
**Burundi**	56 (35.4-88.1)	115475 (72992-181572)
**Cambodia**	51.1 (32.3-80)	90538 (57236-141700)
**Cameroon**	58.8 (36.8-92.4)	219742 (137476-345249)
**Cape Verde**	54.5 (34.3-85.5)	2923 (1840-4581)
**Central African Republic**	55 (34.7-86.2)	38958 (24558-61043)
**Chad**	57.7 (36.2-90.7)	151768 (95236-238755)
**Chile**	48.7 (30.9-76.4)	57031 (36187-89448)
**China**	31 (18.7-50.8)	2581262 (1552250-4229873)
**Colombia**	47.2 (29.9-73.9)	176453 (111638-276234)
**Comoros**	62.8 (39.3-99.1)	7496 (4686-11820)
**Congo**	56.7 (35.7-88.9)	43059 (27084-67509)
**Cook Islands**	50.3 (31.6-79)	106 (66-166)
**Costa Rica**	40.3 (24.5-65.6)	14125 (8595-22989)
**Cote d'Ivoire**	51.9 (32.8-81.5)	190408 (120415-298876)
**Dem. Peoples's Rep. of Korea**	51.1 (32.4-79.8)	89280 (56599-139482)
**Dem. Rep. of the Congo**	54.3 (34.3-85.1)	753225 (475718-1180411)
**Djibouti**	57.3 (36-90)	5848 (3672-9182)
**Dominica**	48.7 (30.8-76.2)	297 (188-464)
**Dominican Republic**	46.9 (29.6-73.8)	49802 (31480-78430)
**Ecuador**	46.6 (29.5-73.1)	75053 (47574-117696)
**Egypt**	50.4 (31.9-78.8)	610777 (386798-955331)
**El Salvador**	53.7 (34-84.1)	27939 (17674-43719)
**Equatorial Guinea**	55.3 (35-86.9)	7093 (4488-11137)

Country	Incidence of RSV-associated ALRI (per 1000 children per year)	No. of episodes of RSV-associated ALRI (UR)
**Eritrea**	57.6 (36.2-90.6)	46916 (29459-73808)
**Ethiopia**	58 (36.4-91.3)	846851 (532193-1333188)
**Fiji**	37.5 (22.8-61)	3299 (2011-5369)
**Gabon**	60.9 (38-95.7)	14554 (9094-22881)
**Gambia**	58.3 (36.5-91.7)	21361 (13378-33601)
**Ghana**	52.8 (33.4-82.9)	214075 (135362-336105)
**Grenada**	48.3 (30.6-75.6)	480 (304-752)
**Guatemala**	54.6 (34.5-86.1)	114073 (72104-179935)
**Guinea**	59.5 (37.1-93.9)	121665 (75809-192047)
**Guinea-Bissau**	58.2 (36.6-91.5)	16822 (10566-26435)
**Guyana**	56 (35.3-87.8)	3758 (2372-5897)
**Haiti**	52.6 (33.2-82.3)	65088 (41121-101880)
**Honduras**	52 (33-81.4)	42428 (26902-66384)
**India**	56.7 (35.7-89)	7013468 (4420047-11010288)
**Indonesia**	50.1 (31.6-78.5)	1245185 (786572-1951996)
**Iran (Islamic Republic of)**	56.6 (35.7-88.9)	387906 (244677-609112)
**Iraq**	54.8 (34.6-86.2)	313987 (197944-493979)
**Jamaica**	48.3 (30.6-75.7)	9849 (6228-15419)
**Jordan**	61.3 (38.6-96.8)	60063 (37877-94943)
**Kenya**	53.8 (34-84.4)	385484 (243738-604648)
**Kiribati**	38.2 (23.2-62)	563 (342-916)
**Kuwait**	55.9 (35.2-88.1)	19450 (12261-30648)
**Lao People's Dem. Republic**	41 (25-66.9)	34428 (20929-56091)
**Lebanon**	56.2 (35.5-88.3)	25907 (16363-40710)
**Lesotho**	58.1 (36.5-91.4)	16167 (10164-25433)
**Liberia**	54.9 (34.4-86)	38516 (24112-60313)
**Libyan Arab Jamahiriya**	55 (34.8-86.4)	35686 (22581-56046)
**Madagascar**	54.5 (34.4-85.5)	205554 (129713-322426)
**Malawi**	58.9 (37.2-92.6)	174043 (109741-273602)
**Malaysia**	38.1 (23.2-62.1)	94332 (57527-153910)
**Maldives**	44.4 (28.1-70.2)	1660 (1049-2622)
**Mali**	58.1 (36.5-91.8)	190123 (119269-300448)
**Marshall Islands**	39.9 (24.3-65.1)	200 (122-327)
**Mauritania**	55.9 (35.2-87.8)	33617 (21171-52756)
**Mauritius**	53.3 (33.7-83.6)	3765 (2379-5905)
**Mexico**	47.4 (30.1-74.3)	550902 (349207-863101)
**Micronesia (Fed. States of)**	40.1 (24.4-65.4)	464 (282-757)
**Mongolia**	37 (22.5-60.1)	12501 (7617-20319)
**Morocco**	54.8 (34.5-86.2)	187360 (118086-294888)
**Mozambique**	58.6 (36.9-91.8)	282071 (177599-442050)
**Myanmar**	40.2 (24.7-65.2)	183560 (112580-297556)

Country	Incidence of RSV-associated ALRI (per 1000 children per year)	No. of episodes of RSV-associated ALRI (UR)
**Namibia**	55.4 (34.9-87.1)	18744 (11809-29458)
**Nauru**	42.1 (25.4-69.1)	40 (24-66)
**Nepal**	54.6 (34.5-85.6)	153176 (96943-240359)
**Nicaragua**	52.7 (33.4-82.7)	31940 (20210-50079)
**Niger**	58.2 (36.3-92.1)	241080 (150641-381852)
**Nigeria**	55.6 (34.9-87.3)	1728622 (1086317-2717239)
**Niue**	41 (24.9-66.8)	6 (3-9)
**Oman**	57 (35.9-89.4)	21954 (13841-34454)
**Pakistan**	63.9 (39.4-101.7)	1575051 (972854-2507724)
**Palau**	37.5 (22.9-61.2)	75 (46-122)
**Panama**	47.6 (30.2-74.4)	17533 (11114-27427)
**Papua New Guinea**	37.9 (23.1-61.9)	37763 (22994-61670)
**Paraguay**	52.3 (33.1-81.7)	35221 (22293-55074)
**Peru**	48.3 (30.6-75.6)	145886 (92275-228312)
**Philippines**	59.8 (37.5-93.9)	673244 (422425-1057080)
**Qatar**	55.8 (35.2-87.6)	7383 (4656-11593)
**Republic of Korea**	35.9 (21.8-58.3)	82022 (49895-133456)
**Rwanda**	50.6 (31.9-79.6)	85681 (54107-134839)
**Saint Kitts and Nevis**	48.6 (30.7-76.1)	223 (141-349)
**Saint Lucia**	48.6 (30.7-76.1)	669 (423-1048)
**Saint Vincent and Grenadines**	48.6 (30.8-76.3)	414 (262-649)
**Samoa**	36.9 (22.5-60.3)	892 (543-1457)
**Sao Tome and Principe**	51.5 (32.5-81)	1523 (962-2395)
**Saudi Arabia**	54.3 (34.3-85.6)	171639 (108456-270632)
**Senegal**	65.6 (40.3-105.1)	170579 (104861-273378)
**Seychelles**	53.1 (33.5-83.4)	445 (281-699)
**Sierra Leone**	55.8 (34.9-87.8)	56009 (35078-88184)
**Solomon Islands**	39.7 (24.1-64.6)	3242 (1969-5284)
**Somalia**	48.7 (30.8-76.9)	95964 (60642-151541)
**South Africa**	52.7 (33.3-82.6)	283103 (178994-443697)
**Sri Lanka**	39.9 (24.1-65.5)	65633 (39649-107543)
**Sudan**	56.9 (35.7-89.4)	338938 (212399-531922)
**Suriname**	47.7 (30.2-74.8)	2299 (1456-3603)
**Swaziland**	59.3 (37.2-93.1)	10277 (6448-16151)
**Syrian Arab Republic**	57.5 (36.3-90.5)	126112 (79599-198435)
**Thailand**	39.4 (23.8-64.3)	149676 (90537-244458)
**Timor-Leste**	42.2 (26.5-66.9)	8597 (5396-13629)
**Togo**	58 (36.4-91.2)	67291 (42204-105766)
**Tonga**	36.6 (22.2-59.5)	472 (287-768)
**Trinidad and Tobago**	47.9 (30.3-75.2)	4602 (2913-7231)
**Tunisia**	51.6 (32.6-81.2)	50711 (31977-79731)

Country	Incidence of RSV-associated ALRI (per 1000 children per year)	No. of episodes of RSV-associated ALRI (UR)
**Tuvalu**	36.6 (22.3-59.4)	37 (22-60)
**Uganda**	58.5 (36.8-92)	425686 (267700-669329)
**United Arab Emirates**	54.8 (34.7-86.3)	26947 (17053-42408)
**United Republic of Tanzania**	56.8 (35.6-89)	533967 (335007-836521)
**Uruguay**	49.4 (31.3-77.4)	11899 (7534-18643)
**Vanuatu**	38.1 (23.2-62.2)	1325 (807-2162)
**Venezuela**	48.1 (30.5-75.5)	142500 (90276-223432)
**Viet Nam**	35.3 (21.4-57.6)	273416 (166008-446120)
**Yemen**	57.2 (35.9-89.9)	224628 (140904-352670)
**Zambia**	57.9 (36.4-90.9)	165095 (103789-259193)
**Zimbabwe**	52.3 (33.1-82)	131042 (82878-205315)

Supplementary table 19: Comparison of the modelled national estimates of RSV-associated ALRI with those reported in the included studies

Country	Observed incidence rate of RSV-ALRI in 0-59m (per 1000 children per year)			RSV-ALRI incidence (modelled estimates)
	Study 1	Study 2	Study 3	
**Bangladesh***	*84.7 (56.4-129.8)*	*26.4 (17.3-40.5)*	17.5 (15.7-19.5)	51.9 (32.9-81.2)
**Brazil**	14 (7.8-25.3)			51.4 (32.5-80.3)
**Germany**	*52.3 (45.5-60.4)*			
**Guatemala**	*84.1 (69.7-115.6)*			54.6 (34.5-86.1)
**India†**	*21.7 (10.8-43.3)*	33.4 (25.2-44.2)		56.7 (35.7-89)
**Indonesia**	65.4 (57.4-74.6)			50.1 (31.6-78.5)
**Kenya**	*59.9 (44.1-81.4)*			53.8 (34-84.4)
**Nicaragua**	*34.7 (17.3-69.3)*			52.7 (33.4-82.7)
**Nigeria**	94 (89.1-99.1)			55.6 (34.9-87.3)
**Pakistan**	35.9 (27.6-46.7)			63.9 (39.4-101.7)
**Peru**	*194.3 (158.2-238.6)*			48.3 (30.6-75.6)
**South Africa**	*188.7 (154.1-230.9)*			52.7 (33.3-82.6)

* There are 3 studies: Dhaka, Bangladesh (Apr 2009 - Mar 2011); Mirzapur, Bangladesh (Oct 1993 - Aug 1996); Dhaka, Bangladesh (Jan 2005 - Feb 2008)
† There are 2 studies: Ballabhgarh, India (Oct 2001 - Mar 2005); Ballabhgarh, India (Aug 2012 - Aug 2013)
Estimates in italics are imputed

Supplementary table 20: In-hospital CFR (%) for RSV-associated ALRI in children younger than 5 years by narrow non-overlapping age bands by World Bank income regions (based on data from 43 studies reporting hCFR estimates by narrow age bands for full 0-59 month age range)

Region	No.*	0-27 days	28-<3m	3-5m	6-8m	9-11m	12-23m	24-59m
**Low income**	9	0	0	3.8 (0.9-15.1)	16.1 (5.2-49.9)	0	6.6 (0.9-47.1)	0
**Lower middle income**	16	6.3 (3.3-12.1)	2.7 (1.7-4.1)	3.6 (2.2-6.00)	2.6 (1.2-5.4)	7.0 (4.0-12.4)	2.5 (1.3-5.1)	5.5 (3.1-10.0)
**Upper middle income**	12	1.0 (0.1-7.2)	1.9 (1.1-3.4)	1.9 (1.1-3.3)	3.0 (1.1-8.1)	2.2 (0.5-8.6)	0.7 (0.1-4.7)	0
**High income**	6	0	2.1 (1.3-3.5)	0.4 (0.0-5.5)	1.2 (0.3-5.6)	1.1 (0.5-2.5)	0.3 (0.0-5.4)	1.2 (0.2-8.2)
**Developing**	41	5.3 (2.8-9.8)	2.3 (1.7-3.0)	2.4 (1.8-3.2)	3.0 (2.1-4.4)	3.6 (2.3-5.6)	2.0 (1.3-3.0)	4.2 (2.6-6.6)
**Industrialised**	2	0	0	0.1 (0.0-0.4)	0.1 (0.0-0.7)	0	0.0 (0.0-0.3)	0.2 (0.1-0.5)

* No.= Number of studies

Supplementary table 21: Method estimating overall RSV-associated ALRI mortality in children younger than 5 years

**Step 1**	Identify sites with community-based data on ALRI (pneumonia) mortality (based on verbal autopsy, mortality survey, or COD provided in vital registration of deaths) along with monthly data on RSV (and influenza) activity (where available) for at least three consecutive years
**Step 2**	Use comparative (excess mortality model) to estimate excess ALRI mortality during RSV season (Supplementary table 20). Ascertain that there is no overlap in peak RSV and influenza activity.
**Step 3**	Attribute all excess mortality during RSV season to RSV. Estimate the proportion of ALRI mortality that can be attributed to RSV (Supplementary table 21).
**Step 4**	Estimate RSV-associated ALRI mortality in the country providing community-based ALRI mortality data.
**Step 5**	Estimate RSV-associated ALRI hospitalisations in above country using hospitalisation rate for developing country. Apply in-hospital CFR to this estimate to calculate the estimated number of in-hospital deaths due to RSV-associated ALRI (Supplementary table 22).
**Step 6**	Compare overall RSV-associated ALRI deaths in given country (step 4) with RSV-associated ALRI in hospital in same country (step 5). This ratio provides the inflation factor.
**Step 7**	Estimate RSV-associated ALRI mortality in developing countries by applying mean inflation factor to the estimated RSV-associated ALRI deaths in hospital in developing countries (Supplementary table 22).<br><br>[59578 (47750-74325) *2.2 = 131072 (105050-163515)]
**Step 8**	Adjust for influenza activity during RSV season by estimating area under the curve for RSV. This ranges from 90-100% based on data from Bangladesh and Argentina. Assume that there is no difference in CFR for RSV and influenza in the community.<br><br>[131072 (105050-163515)*0.9= 117964 (94545-147164)]
**Step 9**	Estimate global RSV-associated ALRI mortality by summing the RSV-associated ALRI mortality in developing countries with RSV-associated ALRI deaths in hospital in industrialised countries.<br><br>[{117964 (94545-147164)} + {237 (78-2202)}] = 118201 (94623-149366)

Supplementary table 22: Estimated RSV-ALRI mortality in children younger than 5 years (using excess mortality models) in three LMICs using pneumonia mortality data in under-five children and concurrent RSV activity

Site	Study year	Months of RSV season (a)	Average ALRI deaths per month during RSV season (b)	Average ALRI deaths per month outside RSV season (c)	Total ALRI deaths per year (d)	Proportion of ALRI deaths due to RSV (%) (e)*	ALRI deaths overall in country (f)[254]	Estimated RSV deaths in country (g)#
**Urban slums, Buenos Aires, Argentina**	2011	4	6.3	1.8	39	46.2	887	409
	2012	4	8.5	0.9	41	74.4	878	653
	2013	4	3.3	0.9	20	47.5	844	401
	**Mean**							**488**
**Rural villages, Multicentre, Bangladesh**	2010	9	3.0	2.7	35	8.6	21917	1879
	2011	4	8.8	5.4	78	17.3	20028	3466
	2012	6	9.5	6.3	95	20.0	18759	3752
	**Mean**							**3032**
**Multiple rural hamlets, Lombok, Indonesia**	2000	6	73.3	62.3	814	8.1	39257	3183
	2001	6	84.8	48.8	802	26.9	37584	10122
	2002	7	45.0	36.8	499	11.5	36020	4143
	**Mean**							**5816**

*RSV=respiratory syncytial virus. ALRI=acute lower respiratory infection. *e= [(b-c)*a]/d. #g=e*f*0.01*

Supplementary table 23: Calculation of inflation factor to estimate overall RSV-ALRI mortality in developing countries

Country	Meta-estimate of hospitalised RSV-ALRI (per 1000 children per year) (h)§	Meta-estimate of hCFR for RSV-ALRI (%) (i)§	Population in mean study period (j)	Estimated in-hospital RSV-ALRI deaths in country (k)*	Estimated RSV deaths in country (g)	Inflation factor (l)#
**Argentina**	4.9	1.9	3407649	320	488	1.5
**Bangladesh**	4.9	1.9	15197631	1426	3032	2.1
**Indonesia**	4.9	1.9	21639000	2030	5816	2.9
**Mean**						**2.2**

*RSV=respiratory syncytial virus. hCFR=in-hospital case fatality ratio. §Meta-estimate in the developing region (where this country comes from) was used. *k=h*j*0.001*i*0.01. #l=g/k*

Supplementary table 24: Sensitivity analysis for estimating RSV-ALRI mortality in children younger than 5 years (using excess mortality models) in three LMICs using pneumonia mortality data in under-five children and concurrent RSV activity (by extending RSV season by one month to capture pneumococcal deaths secondary to RSV infection)

Site	Study year	Months of RSV season (a)	Average ALRI deaths per month during RSV season (b)	Average ALRI deaths per month outside RSV season (c)	Total ALRI deaths in community per year (d)	Proportion of ALRI deaths due to RSV (%) (e)*	ALRI deaths overall in this country (f)[254]	RSV deaths overall in this country (g)#
**Buenos Aires, Argentina**	2011	5	5.6	1.6	39	51.6	887	458
	2012	5	7.4	0.6	41	83.3	878	731
	2013	5	3.0	0.7	20	57.1	844	482
	**Mean**							**557**
**Multicentre, Bangladesh**	2010	11	3.0	2.0	35	31.4	21917	6888
	2011	4	8.8	5.4	78	17.3	20028	3466
	2012	8	9.5	4.8	95	40.0	18759	7504
	**Mean**							**5953**
**Lombok, Indonesia**	2000	7	78.7	52.6	814	22.5	39257	8816
	2001	7	86.3	39.6	802	40.7	37584	15315
	2002	9	43.2	36.7	499	11.8	36020	4259
	**Mean**							**9463**

*RSV=respiratory syncytial virus. ALRI=acute lower respiratory infection. *e=[(b-c)*a]/d. #g=e*f*0.01*

Site	Meta-estimate of hospitalisation rate (per 1000 per year) (h)§	Meta-estimate of in-hospital CFR (%) (i)§	Population in mean study period (j)	RSV deaths in hospital overall (k)*	Mean of RSV deaths in community (g)	Adjustment factor (l)#
**Argentina**	4.9	1.9	3407649	320	557	1.7
**Bangladesh**	4.9	1.9	15197631	1426	5953	4.2
**Indonesia**	4.9	1.9	21639000	2030	9463	4.7
**Mean**						**3.5**

*RSV=respiratory syncytial virus. CFR=case fatality ratio. §Meta-estimate in the developing region (where this country comes from) was used. *k=h*j*0.001*i*0.01. #l=g/k.*

Supplementary table 25: Proportion of eligible cases not tested for RSV and comparison of CFR among children who were tested for RSV (with those not tested)

Location (reference)	% of eligible cases not tested for RSV	Reasons for not testing	Proportion of in-hospital ALRI deaths not tested for RSV (%)	CFR among hospitalised ALRI tested for RSV (%)	CFR among hospitalised ALRI not tested for RSV (%)
**Kilifi, Kenya (PERCH, unpublished)**	0.3 (2/634)	specimen not collected	6.1	4.9	100.0
**Iquique, Chile (Fasce, unpublished)**	0.6 (4/683)	cases were not notified in time so as to collect sample	0.0	0.4	0.0
**Buenos Aires, Argentina (Polack, unpublished)**	0.9 (30/3371)	sample inadequate	23.6	1.3	43.3
**Gauteng province, South Africa (Cohen, unpublished)**	2.0 (122/6016)	child too ill to take sample from; specimens not reaching laboratory within 72 hours	5.7	0.8	2.5
**Manhiça, Mozambique (Bassat, unpublished)**	3.1 (15/491)	NPA not collected or processed without results	14.3	2.5	13.3
**Klerksdorp site, South Africa (Cohen, unpublished)**	3.2 (30/932)	patient very ill; parents refuse consent, specimen not at lab within 72 hours	0.0	2.1	0.0
**Tone District, Togo (Gessner, unpublished)**	3.2 (4/124)	testing just started	100.0	0.0	66.7
**Concepcion, Chile (Fasce, unpublished)**	3.3 (16/481)	cases were not notified in time so as to collect sample	0.0	0.4	0.0
**Manhiça, Mozambique (Bassat, unpublished)**	3.4 (28/835)	NPA not collected or processed without results	8.5	9.3	25.0
**Mpumalanga, South Africa (Cohen, unpublished)**	4.0 (52/1310)	patient very ill; parents refuse consent; specimen not at lab within 72 hours	12.5	6.1	21.2
**Western Gambia (Howie, unpublished)**	4.2 (4/95)	technical problem with specimen	0.0	3.3	0.0
**KwaZulu-Natal province, South Africa (Cohen, unpublished)**	4.5 (72/1607)	patient very ill; parents refuse consent, specimen not at lab within 72 hours	9.1	0.7	1.4

Location (reference)	% of eligible cases not tested for RSV	Reasons for not testing	Proportion of in-hospital ALRI deaths not tested for RSV (%)	CFR among hospitalised ALRI tested for RSV (%)	CFR among hospitalised ALRI not tested for RSV (%)
**Soweto, South Africa (Madhi, unpublished)**	4.6 (240/5231)	missed as part of surveillance	16.3	5.5	22.5
**CEMIC, Buenos Aires, Argentina (Echavarria, unpublished)**	5.3 (3/57)	insufficient sample	NA	0.0	0.0
**Santa Rosa, Guatemala (McCracken, unpublished)**	5.8 (104/1810)	no consent or no specimen	2.2	5.2	1.9
**Basse Santa Su, Gambia (PERCH, unpublished)**	7.2 (46/638)	quality control not yet complete	22.7	2.9	10.9
**Bamako, Mali (PERCH, unpublished)**	9.1 (61/674)	quality control not yet complete	12.7	14.5	21.3
**41 sites in Netherlands (Bont, unpublished)**	10.0 (24/241)	reasons for not testing are unknown, as the decisions for testing are made by independent physicians at hospital of presentation	NA	0.0	0.0
**Lusaka, Zambia (PERCH, unpublished)**	10.5 (65/617)	quality control not yet complete	14.5	18.1	26.2
**Quetzaltenango, Guatemala (McCracken, unpublished)**	10.9 (207/1893)	no consent or no specimen	0.0	2.7	0.0
**Buenos Aires, Argentina (Gentile, unpublished)**	12.9 (1361/10581)	NA	10.8	2.0	1.6
**Paarl, South Africa (Zar, unpublished)**	16.4 (21/128)	specimens were not obtained	50.0	0.9	4.8
**Nakhon Phanom and Sa Kaeo, Thailand (PERCH, unpublished)**	18.3 (41/224)	quality control not yet complete	33.3	1.1	2.4
**Kilifi hospital study, Kenya (Nokes, unpublished)**	24.4 (2817/11544)	died; discharged; refusal	67.0	5.2	32.7
**Bondo district, Kenya (Feikin,**	28.7 (208/726)	NA	43.8	5.2	10.1

Location (reference)	% of eligible cases not tested for RSV	Reasons for not testing	Proportion of in-hospital ALRI deaths not tested for RSV (%)	CFR among hospitalised ALRI tested for RSV (%)	CFR among hospitalised ALRI not tested for RSV (%)
**unpublished)**					
**Lombok, Indonesia (Gessner, unpublished)**	28.9 (1500/5198)	primarily age <3 months and critical illness on presentation; specimens collected 4 days a week so if patients who had rapid admission, would not have been done	86.1	2.2	33.1
**Tagbilaran and 6 rural sites, Philippines (Lucero, unpublished)**	36.0 (537/1491)	no specimen collected for those cases not tested for RSV	70.6	0.5	2.2
**Belo Horizonte, Brazil (Oliveira, unpublished)**	59.4 (757/1275)	frozen samples at weekends; laboratory refused	62.5	1.2	1.3
**Lwak, Kenya (Montgomery, unpublished)**	72.4 (1231/1701)	refusal to consent; unavailability of transport from field site to lab; high patient volumes; inadequate staffing levels	66.0	3.4	2.5
**Sa Kaeo and Nakhon Phanom, Thailand (Thamthitiwat, unpublished)**	75.0 (14334/19103)	patients refused to take part in it	NA	0.0	0.0

Supplementary table 26: hCFR sensitivity analysis assuming that the proportion of RSV in untested (deaths) is same as in those tested

Location (reference)	% of eligible cases not tested for RSV	CFR of tested ALRI (%)	CFR of untested ALRI (%) - a	No. of untested ALRI - b	Proportion of RSV-ALRI - c	No. of RSV+ from untested ALRI - d[*]	Excess RSV mortality from untested ALRI - e	Deaths of RSV-tested ALRI - f	Cases of RSV-tested ALRI - g	CFR of RSV-tested ALRI (%) - h	Adjusted CFR of RSV-ALRI (%) - i
**Kilifi, Kenya (PERCH, unpublished)**	0.3 (2/634)	4.9	100.0	2	0.24	0.47	0.47	6	150	4.00	4.30
**Iquique, Chile (Fasce, unpublished)**	0.6 (4/683)	0.4	0.0	4	0.35	1.40	0.00	2	237	0.84	0.84
**Buenos Aires, Argentina (Polack, unpublished)**	0.9 (30/3371)	1.3	43.3	30	0.65	19.46	8.43	24	2167	1.11	1.48
**Gauteng province, South Africa (Cohen, unpublished)**	2.0 (122/6016)	0.8	2.5	122	0.29	35.04	0.86	8	1693	0.47	0.51
**Manhiça, Mozambique (Bassat, unpublished)**	3.1 (15/491)	2.5	13.3	15	0.14	2.14	0.29	0	68	0.00	0.41
**Klerksdorp site, South Africa (Cohen, unpublished)**	3.2 (30/932)	2.1	0.0	30	0.22	6.72	0.00	3	202	1.49	1.44
**Tone District, Togo (Gessner, unpublished)**	3.2 (4/124)	0.0	66.7	3	0.12	0.37	0.25	0	15	0.00	1.61

[*] Assume the proportion is the same as in the tested ALRI; d=b*c; e=d*a*0.01; h=f/g*100; i=(f+e)/(g+d)*100

Location (reference)	% of eligible cases not tested for RSV	CFR of tested ALRI (%)	CFR of untested ALRI (%) - a	No. of untested ALRI - b	Proportion of RSV-ALRI - c	No. of RSV+ from untested ALRI - d*	Excess RSV mortality from untested ALRI - e	Deaths of RSV-tested ALRI - f	Cases of RSV-tested ALRI - g	CFR of RSV-tested ALRI (%) - h	Adjusted CFR of RSV-ALRI (%) - i
**Concepcion, Chile (Fasce, unpublished)**	3.3 (16/481)	0.4	0.0	16	0.43	6.92	0.00	1	201	0.50	0.48
**Manhiça, Mozambique (Bassat, unpublished)**	3.4 (28/835)	9.3	25.0	28	0.06	1.70	0.43	1	49	2.04	2.81
**Mpumalanga, South Africa (Cohen, unpublished)**	4.0 (52/1310)	6.1	21.2	52	0.21	11.08	2.34	6	268	2.24	2.99
**Western Gambia (Howie, unpublished)**	4.2 (4/95)	3.3	0.0	100	0.18	17.58	0.00	0	16	0.00	0.00
**KwaZulu-Natal province, South Africa (Cohen, unpublished)**	4.5 (72/1607)	0.7	1.4	72	0.28	20.22	0.28	0	431	0.00	0.06
**Soweto, South Africa (Madhi, unpublished)**	4.6 (240/5231)	5.5	22.5	240	0.17	41.50	9.34	14	863	1.62	2.58
**CEMIC, Buenos Aires, Argentina (Echavarria, unpublished)**	5.3 (3/57)	0.0	0.0	3	0.39	1.17	0.00	0	21	0.00	0.00

Location (reference)	% of eligible cases not tested for RSV	CFR of tested ALRI (%)	CFR of untested ALRI (%) - a	No. of untested ALRI - b	Proportion of RSV-ALRI - c	No. of RSV+ from untested ALRI - d*	Excess RSV mortality from untested ALRI - e	Deaths of RSV-tested ALRI - f	Cases of RSV-tested ALRI - g	CFR of RSV-tested ALRI (%) - h	Adjusted CFR of RSV-ALRI (%) - i
**Santa Rosa, Guatemala (McCracken, unpublished)**	5.8 (104/1810)	5.2	1.9	104	0.41	34.10	0.66	15	505	2.97	2.90
**Basse Santa Su, Gambia (PERCH, unpublished)**	7.2 (46/638)	2.9	10.9	46	0.19	8.86	0.96	0	114	0.00	0.78
**Bamako, Mali (PERCH, unpublished)**	9.1 (61/674)	14.5	21.3	61	0.25	15.52	3.31	4	156	2.56	4.26
**41 sites in Netherlands (Bont, unpublished)**	10 (24/241)	0.0	0.0	24	0.72	17.25	0.00	0	156	0.00	0.00
**Lusaka, Zambia (PERCH, unpublished)**	10.5 (65/617)	18.1	26.2	65	0.19	12.13	3.17	6	103	5.83	7.97
**Quetzaltenango, Guatemala (McCracken, unpublished)**	10.9 (207/1893)	2.7	0.0	207	0.37	69.28	0.00	8	628	1.27	1.15
**Buenos Aires, Argentina (Gentile, unpublished)**	12.9 (1361/10581)	2.0	1.6	1361	0.39	529.93	8.57	65	3590	1.81	1.79
**Paarl, South Africa (Zar, unpublished)**	16.4 (21/128)	0.9	4.8	21	0.26	5.50	0.26	0	28	0.00	0.78

Location (reference)	% of eligible cases not tested for RSV	CFR of tested ALRI (%)	CFR of untested ALRI (%) - a	No. of untested ALRI - b	Proportion of RSV-ALRI - c	No. of RSV+ from untested ALRI - d*	Excess RSV mortality from untested ALRI - e	Deaths of RSV-tested ALRI - f	Cases of RSV-tested ALRI - g	CFR of RSV-tested ALRI (%) - h	Adjusted CFR of RSV-ALRI (%) - i
**Nakhon Phanom and Sa Kaeo, Thailand (PERCH, unpublished)**	18.3 (41/224)	1.1	2.4	41	0.27	11.20	0.27	0	50	0.00	0.45
**Kilifi hospital study, Kenya (Nokes, unpublished)**	24.4 (2817/11544)	5.2	32.7	2817	0.16	460.95	150.87	31	1428	2.17	9.63
**Bondo district, Kenya (Feikin, unpublished)**	28.7 (208/726)	5.2	10.1	208	0.13	26.50	2.68	1	66	1.52	3.97
**Lombok, Indonesia (Gessner, unpublished)**	28.9 (1500/5198)	2.2	33.1	1500	0.20	300.57	99.59	13	741	1.75	10.81
**Tagbilaran and 6 rural sites, Philippines (Lucero, unpublished)**	36 (537/1491)	0.5	2.2	537	0.37	200.95	4.49	2	357	0.56	1.16
**Belo Horizonte, Brazil (Oliveira, unpublished)**	59.4 (757/1275)	1.2	1.3	757	0.29	222.13	2.93	2	152	1.32	1.32
**Lwak, Kenya (Montgomery, unpublished)**	72.4 (1231/1701)	3.4	2.5	1231	0.21	264.53	6.66	4	101	3.96	2.92

Location (reference)	% of eligible cases not tested for RSV	CFR of tested ALRI (%)	CFR of untested ALRI (%) - a	No. of untested ALRI - b	Proportion of RSV-ALRI - c	No. of RSV+ from untested ALRI - d*	Excess RSV mortality from untested ALRI - e	Deaths of RSV-tested ALRI - f	Cases of RSV-tested ALRI - g	CFR of RSV-tested ALRI (%) - h	Adjusted CFR of RSV-ALRI (%) - i
**Sa Kaeo and Nakhon Phanom, Thailand (Thamthitiwat, unpublished)**	75 (14334/19103)	0.0	0.0	14334	0.17	2389.50	0.00	0	795	0.00	0.00

hCFR meta-estimate in children aged 0-59m from developing countries using data from above 30 studies reporting deaths in hospitalised RSV positive cases – 1.5 (0.9 to 2.3) (A)

hCFR meta-estimate in children aged 0-59m from developing countries using data from above 30 studies reporting deaths in hospitalised RSV positive cases and assuming that proportion of RSV positive in those not tested is same as in those tested– 1.6 (1.0 to 2.5) (B)

Adjustment factor to account for this assumption = B/A = 1.07

Supplementary table 27: hCFR sensitivity analysis assuming that all deaths in untested ALRI cases are RSV positive

Location (reference)	% of eligible cases not tested for RSV	CFR of tested ALRI (%)	CFR of untested ALRI (%) - a	No. of untested ALRI - b	Deaths in RSV-tested ALRI - f	Cases of RSV-tested ALRI - g	CFR in RSV-tested ALRI (%) - h	Deaths in RSV untested ALRI - j†	Adjusted CFR in RSV-ALRI (%) - i
**Kilifi, Kenya (PERCH, unpublished)**	0.3 (2/634)	4.9	100.0	2	6	150	4.00	2	5.26
**Iquique, Chile (Fasce, unpublished)**	0.6 (4/683)	0.4	0.0	4	2	237	0.84	0	0.83
**Buenos Aires, Argentina (Polack, unpublished)**	0.9 (30/3371)	1.3	43.3	30	24	2167	1.11	13	1.68
**Gauteng province, South Africa (Cohen, unpublished)**	2 (122/6016)	0.8	2.5	122	8	1693	0.47	3	0.61
**Manhiça, Mozambique (Bassat, unpublished)**	3.1 (15/491)	2.5	13.3	15	0	68	0.00	2	2.41
**Klerksdorp site, South Africa (Cohen, unpublished)**	3.2 (30/932)	2.1	0.0	30	3	202	1.49	0	1.29
**Tone District, Togo (Gessner, unpublished)**	3.2 (4/124)	0.0	66.7	3	0	15	0.00	2	11.11

† Assume all deaths in untested ALRI cases are RSV positive; h=f/g*100; i=(f+j)/(g+b)*100

Location (reference)	% of eligible cases not tested for RSV	CFR of tested ALRI (%)	CFR of untested ALRI (%) - a	No. of untested ALRI - b	Deaths in RSV-tested ALRI - f	Cases of RSV-tested ALRI - g	CFR in RSV-tested ALRI (%) - h	Deaths in RSV untested ALRI - j[†]	Adjusted CFR in RSV-ALRI (%) - i
**Concepcion, Chile (Fasce, unpublished)**	3.3 (16/481)	0.4	0.0	16	1	201	0.50	0	0.46
**Manhiça, Mozambique (Bassat, unpublished)**	3.4 (28/835)	9.3	25.0	28	1	49	2.04	7	10.39
**Mpumalanga, South Africa (Cohen, unpublished)**	4 (52/1310)	6.1	21.2	52	6	268	2.24	11	5.31
**Western Gambia (Howie, unpublished)**	4.2 (4/95)	3.3	0.0	100	0	16	0.00	0	0.00
**KwaZulu-Natal province, South Africa (Cohen, unpublished)**	4.5 (72/1607)	0.7	1.4	72	0	431	0.00	1	0.20
**Soweto, South Africa (Madhi, unpublished)**	4.6 (240/5231)	5.5	22.5	240	14	863	1.62	54	6.17
**CEMIC, Buenos Aires, Argentina (Echavarria, unpublished)**	5.3 (3/57)	0.0	0.0	3	0	21	0.00	0	0.00
**Santa Rosa, Guatemala (McCracken, unpublished)**	5.8 (104/1810)	5.2	1.9	104	15	505	2.97	2	2.79

Location (reference)	% of eligible cases not tested for RSV	CFR of tested ALRI (%)	CFR of untested ALRI (%) - a	No. of untested ALRI - b	Deaths in RSV-tested ALRI - f	Cases of RSV-tested ALRI - g	CFR in RSV-tested ALRI (%) - h	Deaths in RSV untested ALRI - j[†]	Adjusted CFR in RSV-ALRI (%) - i
**Basse Santa Su, Gambia (PERCH, unpublished)**	7.2 (46/638)	2.9	10.9	46	0	114	0.00	5	3.13
**Bamako, Mali (PERCH, unpublished)**	9.1 (61/674)	14.5	21.3	61	4	156	2.56	13	7.83
**41 sites in Netherlands (Bont, unpublished)**	10 (24/241)	0.0	0.0	24	0	156	0.00	0	0.00
**Lusaka, Zambia (PERCH, unpublished)**	10.5 (65/617)	18.1	26.2	65	6	103	5.83	17	13.69
**Quetzaltenango, Guatemala (McCracken, unpublished)**	10.9 (207/1893)	2.7	0.0	207	8	628	1.27	0	0.96
**Buenos Aires, Argentina (Gentile, unpublished)**	12.9 (1361/10581)	2.0	1.6	1361	65	3590	1.81	22	1.76
**Paarl, South Africa (Zar, unpublished)**	16.4 (21/128)	0.9	4.8	21	0	28	0.00	1	2.04
**Nakhon Phanom and Sa Kaeo, Thailand (PERCH,**	18.3 (41/224)	1.1	2.4	41	0	50	0.00	1	1.10

Location (reference)	% of eligible cases not tested for RSV	CFR of tested ALRI (%)	CFR of untested ALRI (%) - a	No. of untested ALRI - b	Deaths in RSV-tested ALRI - f	Cases of RSV-tested ALRI - g	CFR in RSV-tested ALRI (%) - h	Deaths in RSV untested ALRI - j[†]	Adjusted CFR in RSV-ALRI (%) - i
**unpublished)**									
**Kilifi hospital study, Kenya (Nokes, unpublished)**	24.4 (2817/11544)	5.2	32.7	2817	31	1428	2.17	922	22.45
**Bondo district, Kenya (Feikin, unpublished)**	28.7 (208/726)	5.2	10.1	208	1	66	1.52	21	8.03
**Lombok, Indonesia (Gessner, unpublished)**	28.9 (1500/5198)	2.2	33.1	1500	13	741	1.75	497	22.76
**Tagbilaran and 6 rural sites, Philippines (Lucero, unpublished)**	36.0 (537/1491)	0.5	2.2	537	2	357	0.56	12	1.57
**Belo Horizonte, Brazil (Oliveira, unpublished)**	59.4 (757/1275)	1.2	1.3	757	2	152	1.32	10	1.32
**Lwak, Kenya (Montgomery, unpublished)**	72.4 (1231/1701)	3.4	2.5	1231	4	101	3.96	31	2.63

Location (reference)	% of eligible cases not tested for RSV	CFR of tested ALRI (%)	CFR of untested ALRI (%) - a	No. of untested ALRI - b	Deaths in RSV-tested ALRI - f	Cases of RSV-tested ALRI - g	CFR in RSV-tested ALRI (%) - h	Deaths in RSV untested ALRI - j[†]	Adjusted CFR in RSV-ALRI (%) - i
**Sa Kaeo and Nakhon Phanom, Thailand (Thamthitiwat, unpublished)**	75.0 (14334/19103)	0.0	0.0	14334	0	795	0.00	0	0.00

hCFR meta-estimate in children aged 0-59m from developing countries using data from above 30 studies reporting deaths in hospitalised RSV positive cases – 1.5 (0.9 to 2.3) (A)

hCFR meta-estimate in children aged 0-59m from developing countries using data from above 30 studies reporting deaths in hospitalised RSV positive cases and assuming that all deaths in untested ALRI are RSV positive – 2.1 (1.2 to 3.5) (B)

Adjustment factor to account for this assumption = B/A = 1.4

Supplementary table 28: Sensitivity analysis of in-hospital mortality estimates in developing countries from a sub-set of 22 studies reporting data by narrow age bands through the first year of life

Age group	0-27 days	28 days - <3 months	3-5 months	6-8 months	9-11 months	12-23 months	24-59 months	Overall
**CFR**	5.3 (2.8-9.8)	2.1 (1.6-2.9)	2.2 (1.6-3.0)	2.8 (1.9-4.1)	3.0 (1.9-4.8)	1.7 (1.0-2.7)	4.0 (2.5-6.5)	--
**No. of deaths**	8600 (3700-20400)	11400 (6500-19700)	13700 (7800-24400)	10200 (5800-18100)	10200 (5300-19700)	9900 (5800-17100)	13900 (7500-25700)	81200 (64200-103400)

Supplementary table 29: Sensitivity analysis for in-hospital CFR for RSV-ALRI after excluding studies with <50 RSV cases

		0-5m	6-11 m	12-59 m
	No. of studies	CFR	CFR	CFR
**Low income**	4	1.7 (0.4-6.8)	5.6 (0.8-39.4)	4.8 (0.7-33.8)
**Lower middle income**	13	2.7 (2.0-3.6)	2.8 (1.8-4.4)	2.7 (1.7-4.3)
**Upper middle income**	9	1.8 (1.2-2.6)	2.4 (1.1-5.4)	0.5 (0.1-3.5)
**High income**	5	0.2 (0.0-14.3)	0.9 (0.2-4.3)	0.7 (0.1-5.4)
**Developing**	29	2.2 (1.8-2.7)	2.28 (1.7-3.0)	2.2 (1.6-3.0)
**Industrialised**	2	0.02 (0.0-0.1)	0.05 (0.01-0.4)	0.1 (0.03-0.3)

Supplementary table 30: Sensitivity analysis for in-hospital CFR for RSV-ALRI after excluding studies with <100 RSV cases

		0-5m	6-11 m	12-59 m
	No.	CFR	CFR	CFR
**Low income**	3	1.7 (0.4-6.8)	5.6 (0.8-39.4)	4.8 (0.7-33.8)
**Lower middle income**	11	2.8 (2.0-3.8)	2.7 (1.7-4.2)	2.7 (1.7-4.3)
**Upper middle income**	8	1.8 (1.2-2.6)	2.4 (1.1-5.4)	0.5 (0.1-3.5)
**High income**	5	0.2 (0.0-14.3)	0.9 (0.2-4.3)	0.7 (0.1-5.4)
**Developing**	25	2.2 (1.8-2.7)	2.2 (1.7-3.0)	2.2 (1.6-3.0)
**Industrialised**	2	0.02 (0.00-0.13)	0.05 (0.01-0.38)	0.1 (0.03-0.3)

Supplementary table 31: Definition of hypoxemia in included studies

Location (reference)	Prop of RSV cases with recorded SpO2 (%)	Definition of hypoxemia
Matlab, Bangladesh (PERCH)	100.00	SpO2 <90% or based on supplemental oxygen at altitude ≤2500 m
Basse, Gambia (PERCH)	100.00	SpO2 <90% or based on supplemental oxygen at altitude ≤2500 m
Santa Rosa, Guatemala (McCracken and colleagues)	93.00	SpO2 <90% for 1-59 months and <88% for neonates at altitude ≤2500 m
Dhaka, Bangladesh (PERCH)	100.00	SpO2 <90% or based on supplemental oxygen at altitude ≤2500 m
Tacloban, Philippines (Lupisan and colleagues)	100.00	SpO2 <90% (at altitude ≤2500 m) in children aged 1-59 months and <88% for neonates
Kilifi hospital study, Kenya (Nokes and colleagues)	99.93	SpO2 <90% for all at altitude ≤2500 m
Bondo district, Kenya (Feikin and colleagues)	100.00	SpO2 <90% for 1-59 months and <88% for neonates at altitude ≤2500 m (at sea level)
Rabat, Morocco (Bassat and colleagues)	93.38	SpO2 <90% for all at altitude ≤2500 m
Nakhon Phanom and Sa Kaeo, Thailand (PERCH)	100.00	SpO2 <90% or based on supplemental oxygen at altitude ≤2500 m
David City, Panama (Jara and colleagues)	71.86	SpO2 <90% for 1-59 months and <88% for neonates at altitude ≤2500 m (at sea level)
Berlin, Germany (Rath and colleagues)	78.11	SpO2 <90% for 1-59 months and <88% for neonates at altitude ≤0.942500 m
Soweto, South Africa (Madhi and colleagues)	99.65	SpO2 <90% for 1-59 months
Kilifi, Kenya (PERCH)	100.00	SpO2 <90% or based on supplemental oxygen at altitude ≤2500 m
Lusaka, Zambia (PERCH)	97.09	SpO2 <90% or based on supplemental oxygen at altitude ≤2500 m
Bamako, Mali (PERCH)	100.00	SpO2 <90% or based on supplemental oxygen at altitude ≤2500 m
Manhiça, Mozambique (Bassat and colleagues)	100.00	SpO2 <90% for all at altitude ≤2500 m
Damanhour, Egypt [10]	27.41	SpO2 <90% for all at altitude ≤2500 m
Sa Kaeo and Nakhon Phanom, Thailand (Thamthitiwat and colleagues)	51.95	SpO2 <90% for 1-59 months and <88% for neonates at altitude ≤2500 m (at sea level)
Buenos Aires, Argentina (Gentile and colleagues)	99.94	SpO2 <90% for 1-59 months and <88% for neonates at altitude ≤2500 m (at sea level)
Colorado, USA (Simoes and colleagues)	100.00	ICD-9 codes
Maela Camp, Tak Province, Thailand (Turner and colleagues)	93.02	SpO2 <90% for 1-59 months and <88% for neonates at altitude ≤2500 m (at sea level)

Tagbilaran and Dauis, Philippines (Lucero and colleagues)	NA	SpO2 <90% for 1-59 months and <88% for neonates at altitude ≤2500 m (at sea level)
Alaska, USA (Singleton and colleagues)#	100.00	SpO2 <90% for all at altitude ≤2500 m
Lombok, Indonesia (Gessner and colleagues)	99.19	SpO2 <90% for 1-59 months and <88% for neonates at altitude ≤2500 m (at sea level)
Amman, Jordan (Khuri and colleagues)	100.00	SpO2 <90% for all at altitude ≤2500 m
Paarl, South Africa (Zar and colleagues)	92.86	SpO2 <90% for 1-59 months and <88% for neonates at altitude ≤2500 m
Buenos Aires, Argentina (Polack and colleagues)	98.71	SpO2 <90% for 1-59 months and <88% for neonates at altitude ≤2500 m
Navajo and WMA, USA - EPI (O'Brien and colleagues)#	92.05	SpO2 <90% for 1-59 months and <88% for neonates at altitude ≤2500 m (at sea level)

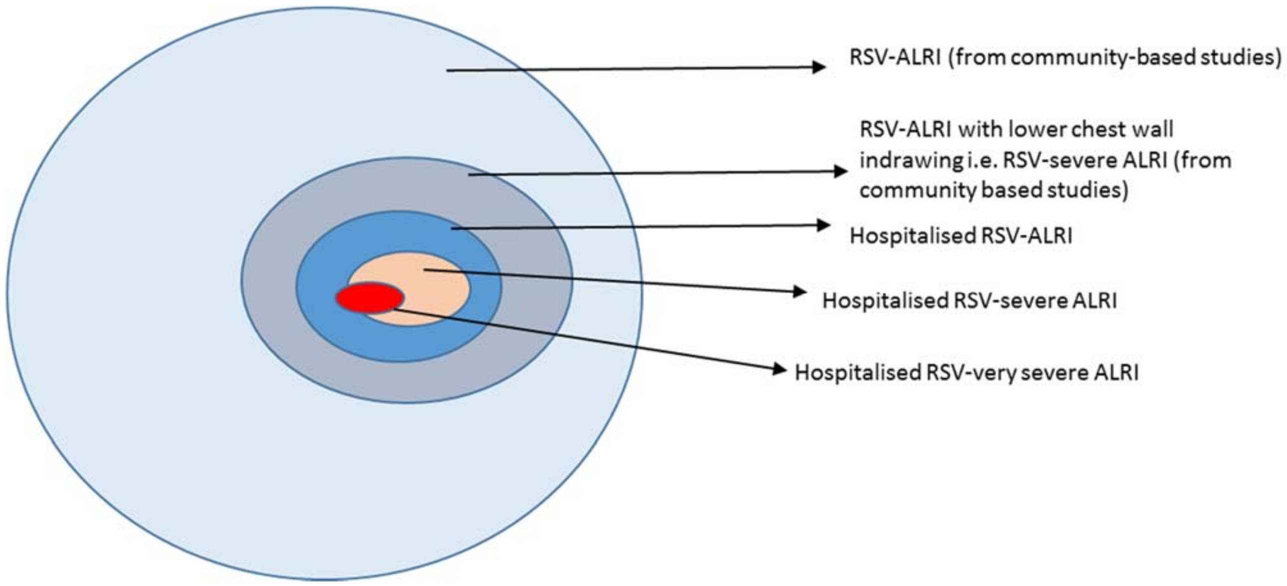

Supplementary figure 1: Conceptual diagram showing how the various categories of disease severity relate to each other

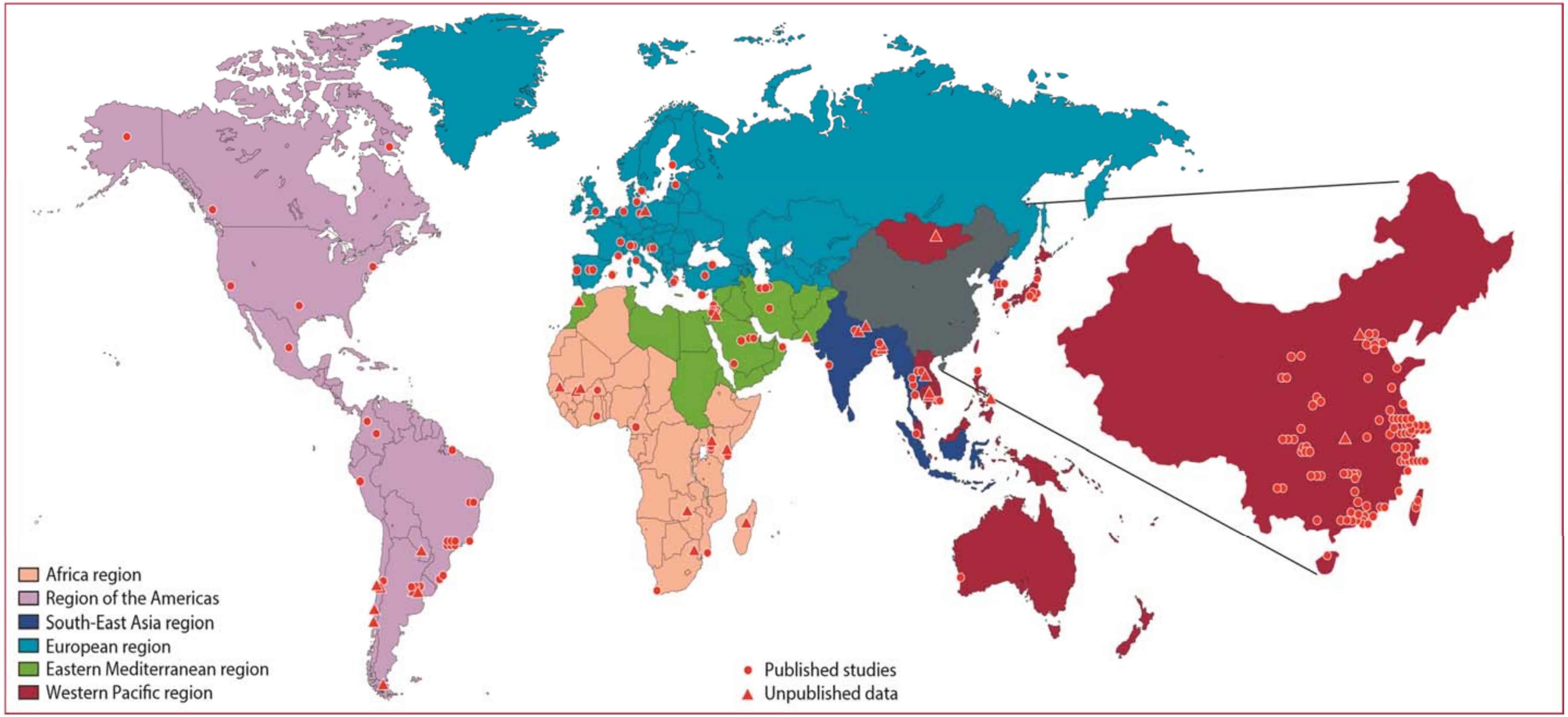

Supplementary figure 2: Location of hospital-based studies reporting proportion of ALRI cases positive for RSV

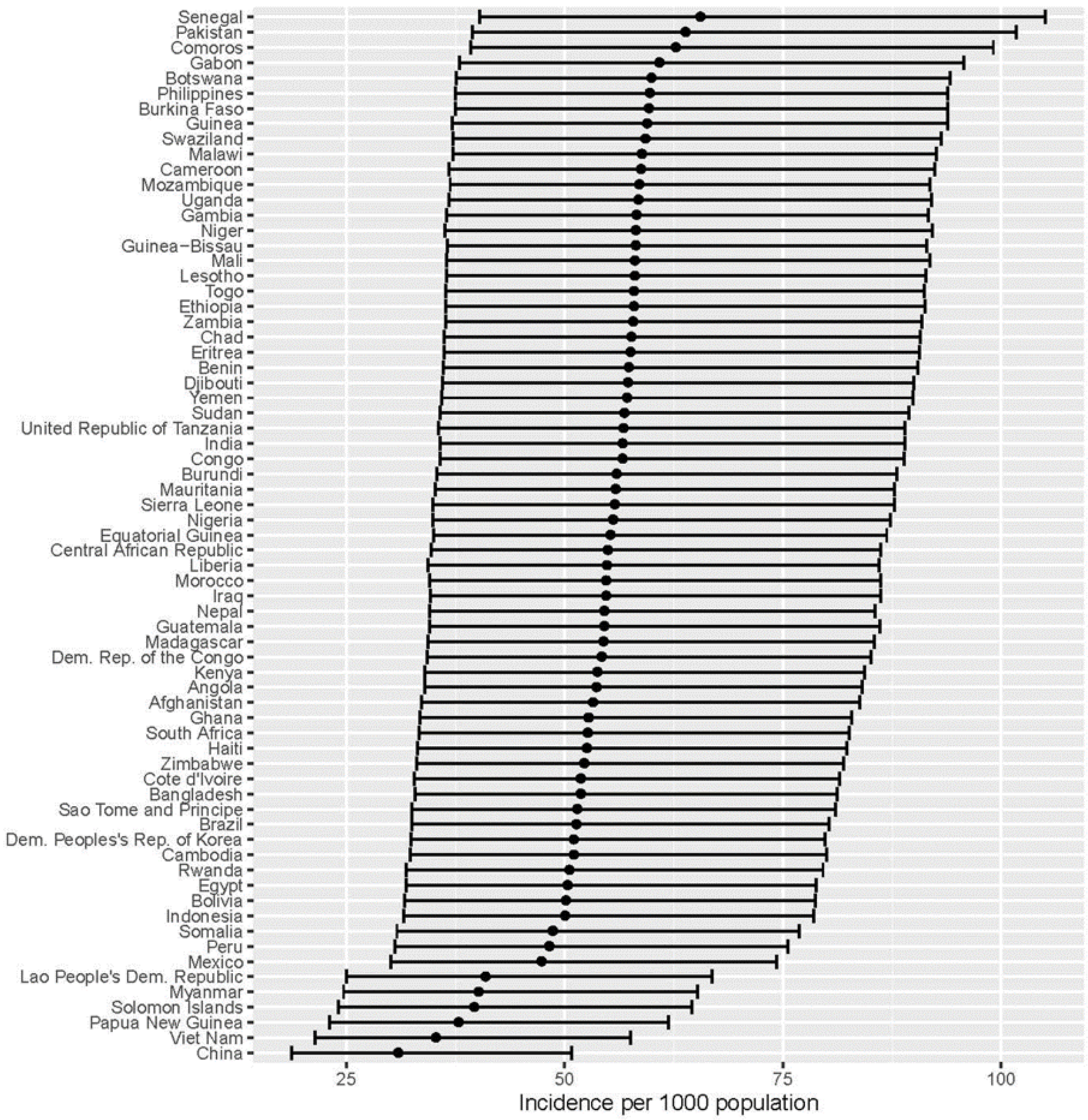

Supplementary figure 3: Incidence of RSV-associated ALRI (per 1000 children aged under 5 years per year) in 69 countdown countries

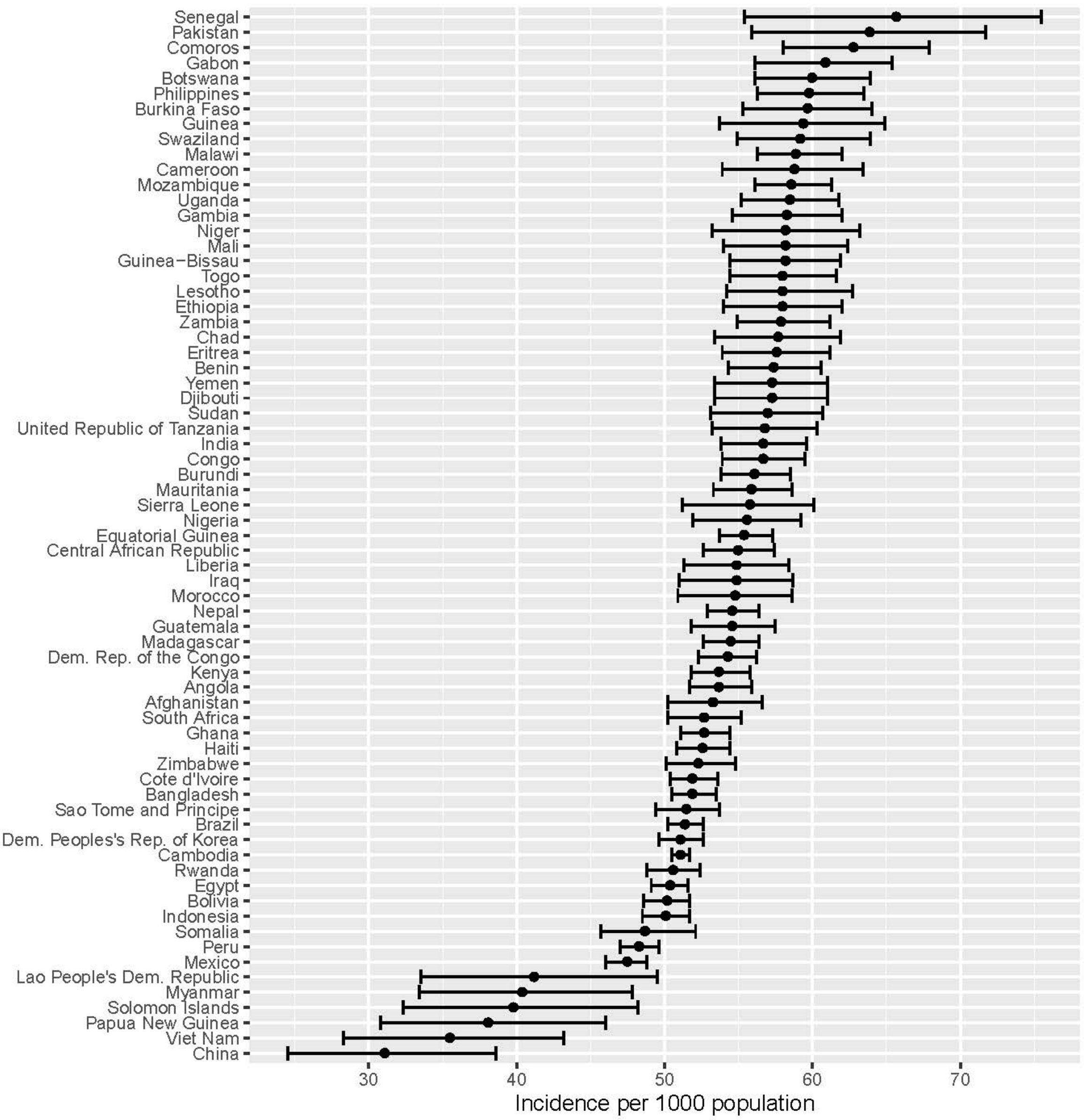

Supplementary figure 4: Incidence of RSV-associated ALRI (per 1000 children younger than 5 years per year) in 69 countdown countries if there were no uncertainty in meta-estimates of incidence of RSV-associated ALRI in developing countries

Supplementary figure 5: Annual variation in the incidence and hospitalisation rates for RSV-associated ALRI among children aged below 5 years at various locations across the world

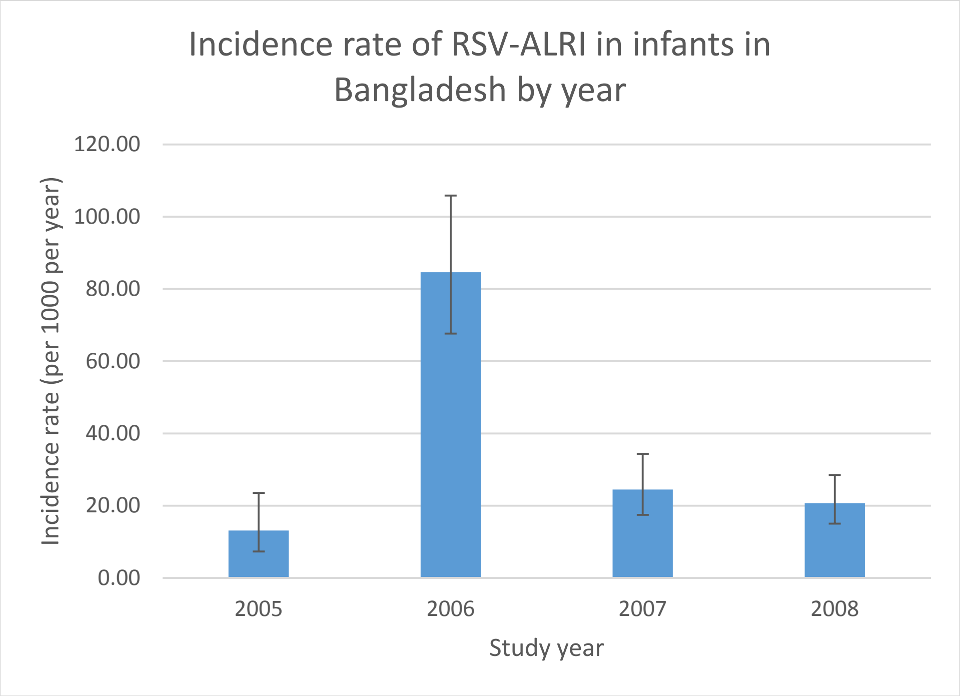

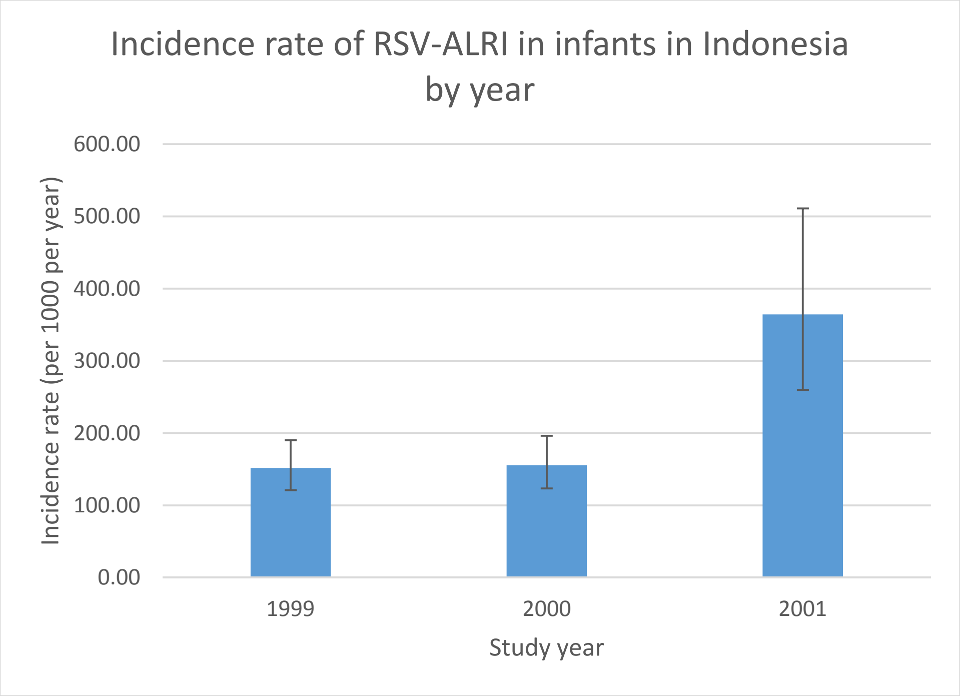

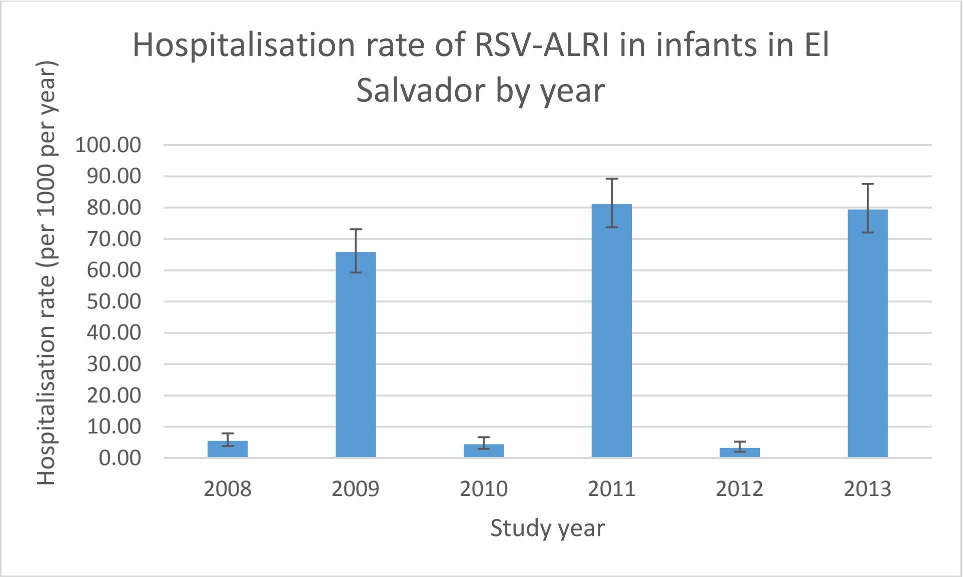

Hospitalisation rate of RSV-ALRI in infants in El Salvador by year
Hospitalisation rate (per 1000 per year)
100.00
90.00
80.00
70.00
60.00
50.00
40.00
30.00
20.00
10.00
0.00
2008
2009
2010
2011
2012
2013
Study year

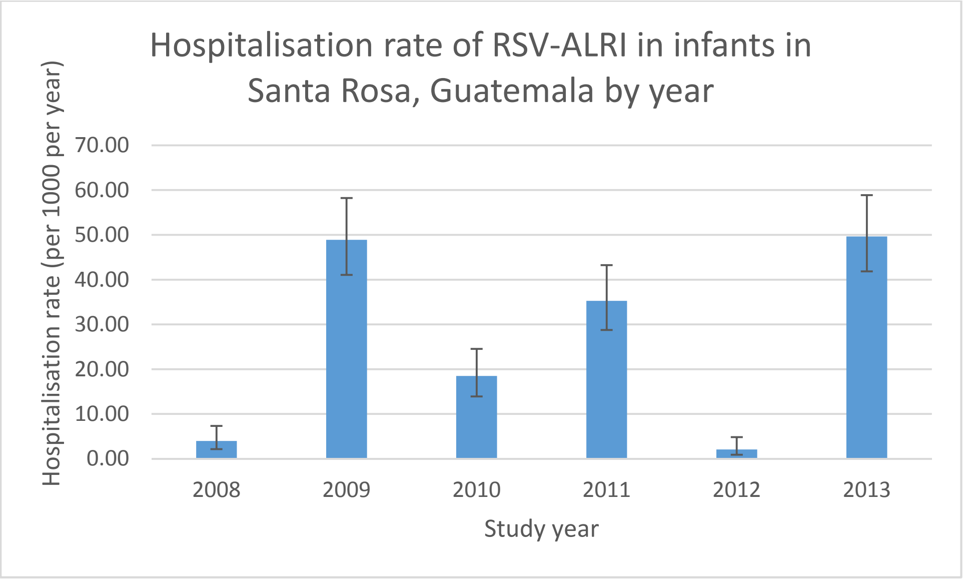

Hospitalisation rate of RSV-ALRI in infants in Santa Rosa, Guatemala by year
Hospitalisation rate (per 1000 per year)
70.00
60.00
50.00
40.00
30.00
20.00
10.00
0.00
2008
2009
2010
2011
2012
2013
Study year

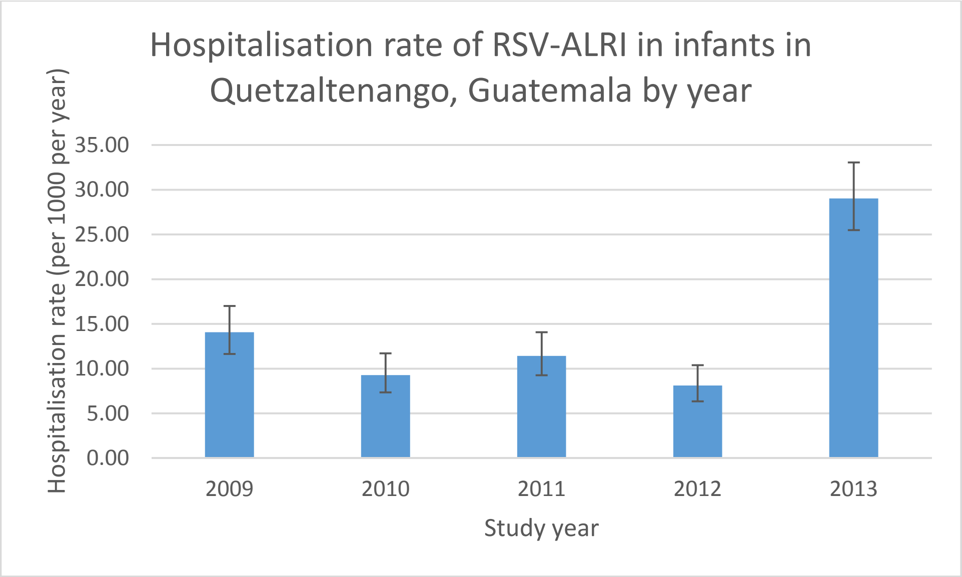
Hospitalisation rate of RSV-ALRI in infants in Quetzaltenango, Guatemala by year
Hospitalisation rate (per 1000 per year)
35.00
30.00
25.00
20.00
15.00
10.00
5.00
0.00
2009
2010
2011
2012
2013
Study year

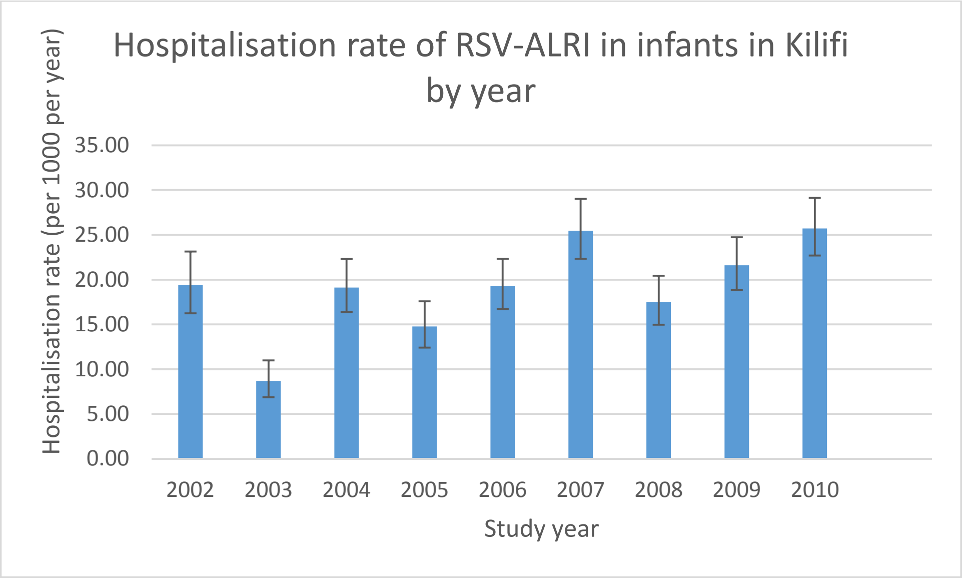
Hospitalisation rate of RSV-ALRI in infants in Kilifi by year
Hospitalisation rate (per 1000 per year)
35.00
30.00
25.00
20.00
15.00
10.00
5.00
0.00
2002
2003
2004
2005
2006
2007
2008
2009
2010
Study year

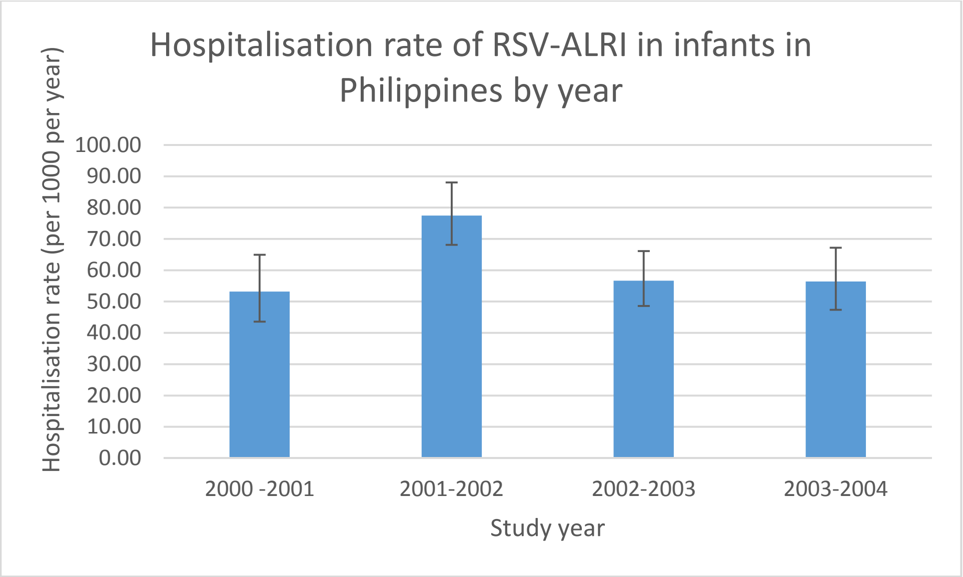
Hospitalisation rate of RSV-ALRI in infants in Philippines by year
Hospitalisation rate (per 1000 per year)
100.00
90.00
80.00
70.00
60.00
50.00
40.00
30.00
20.00
10.00
0.00
2000 -2001
2001-2002
2002-2003
2003-2004
Study year

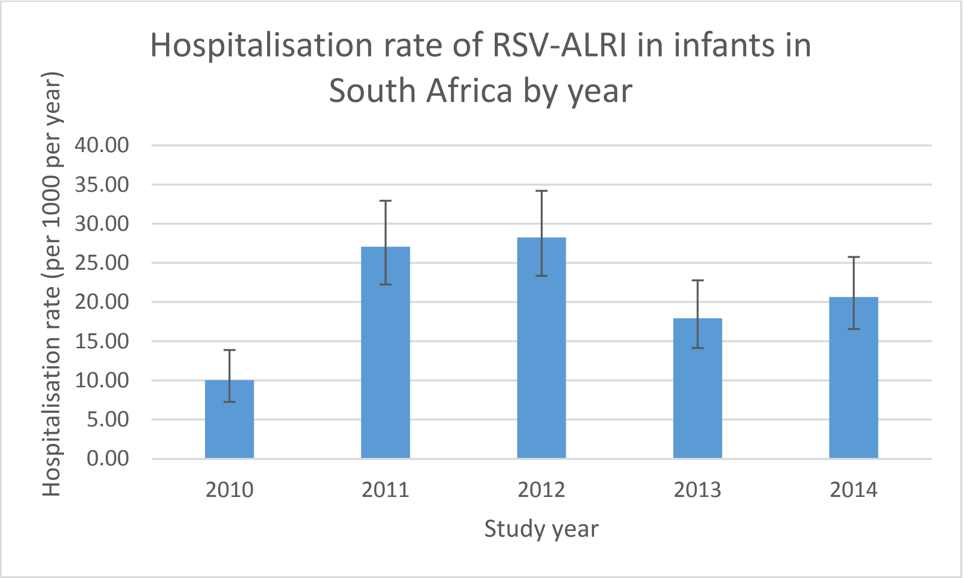
Hospitalisation rate of RSV-ALRI in infants in South Africa by year
Hospitalisation rate (per 1000 per year)
40.00
35.00
30.00
25.00
20.00
15.00
10.00
5.00
0.00
2010
2011
2012
2013
2014
Study year

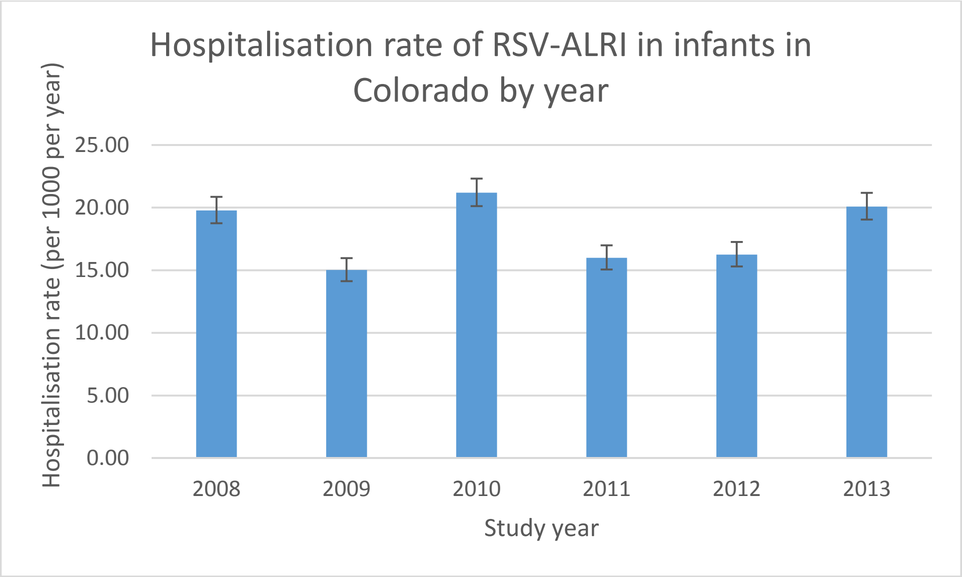
Hospitalisation rate of RSV-ALRI in infants in Colorado by year
Hospitalisation rate (per 1000 per year)
25.00
20.00
15.00
10.00
5.00
0.00
2008
2009
2010
2011
2012
2013
Study year

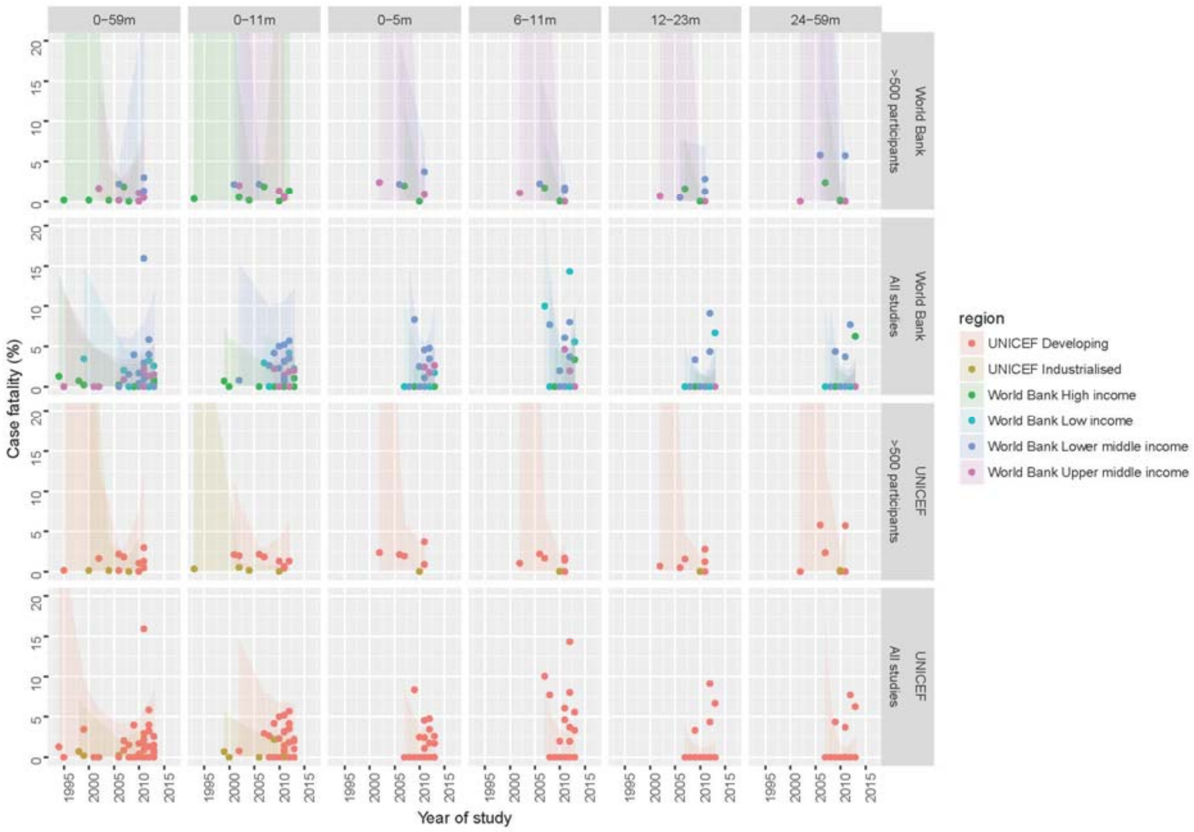

Supplementary figure 6: Trends in in-hospital case fatality ratio for RSV-associated ALRI in children younger than 5 years, by age group and by region

Checklist of information that should be included in new reports of global health estimates

Item #	Checklist item	Reported on page #
**Objectives and funding**		
1	Define the indicator(s), populations (including age, sex, and geographic entities), and time period(s) for which estimates were made.	2
2	List the funding sources for the work.	12
**Data Inputs**		
For all data inputs from multiple sources that are synthesized as part of the study:		
3	Describe how the data were identified and how the data were accessed.	2
4	Specify the inclusion and exclusion criteria. Identify all ad-hoc exclusions.	2
5	Provide information on all included data sources and their main characteristics. For each data source used, report reference information or contact name/institution, population represented, data collection method, year(s) of data collection, sex and age range, diagnostic criteria or measurement method, and sample size, as relevant.	13-38 in supplementary material
6	Identify and describe any categories of input data that have potentially important biases (e.g., based on characteristics listed in item 5).	3
For data inputs that contribute to the analysis but were not synthesized as part of the study:		
7	Describe and give sources for any other data inputs.	2
For all data inputs:		
8	Provide all data inputs in a file format from which data can be efficiently extracted (e.g., a spreadsheet rather than a PDF), including all relevant meta-data listed in item 5. For any data inputs that cannot be shared because of ethical or legal reasons, such as third-party ownership, provide a contact name or the name of the institution that retains the right to the data.	Can be accessed from the web link- is http://datashare.is.ed.ac.uk/handle/10283/2115
**Data analysis**		
9	Provide a conceptual overview of the data analysis method. A diagram may be helpful.	4
10	Provide a detailed description of all steps of the analysis, including mathematical formulae. This description should cover, as relevant, data cleaning, data pre-processing, data adjustments and weighting of data sources, and mathematical or statistical model(s).	3-4
11	Describe how candidate models were evaluated and how the final model(s) were selected.	3-4
12	Provide the results of an evaluation of model performance, if done, as well as the results of any relevant sensitivity analysis.	9
13	Describe methods for calculating uncertainty of the estimates. State which sources of uncertainty were, and were not, accounted for in the uncertainty analysis.	3, 9-11
14	State how analytic or statistical source code used to generate estimates can be accessed.	3
**Results and Discussion**		
15	Provide published estimates in a file format from which data can be efficiently extracted.	Page 7-9 in published article and pages 41-48, 58-61 in supplementa

References

1. Robertson SE, Roca A, Alonso P, et al. Respiratory syncytial virus infection: denominator-based studies in Indonesia, Mozambique, Nigeria and South Africa. *Bull World Health Organ* 2004; **82**(12): 914-22.
2. Ahmed JA, Katz MA, Auko E, et al. Epidemiology of respiratory viral infections in two long-term refugee camps in Kenya, 2007-2010. *BMC Infect Dis* 2012; **12**: 7.
3. Chiu SS, Chan KH, Chen H, et al. Virologically confirmed population-based burden of hospitalization caused by respiratory syncytial virus, adenovirus, and parainfluenza viruses in children in Hong Kong. *Pediatr Infect Dis J* 2010; **29**(12): 1088-92.
4. Fry AM, Chittaganpitch M, Baggett HC, et al. The burden of hospitalized lower respiratory tract infection due to respiratory syncytial virus in rural Thailand. *PLoS ONE [Electronic Resource]* 2010; **5**(11): e15098.
5. Gil-Prieto R, Gonzalez-Escalada A, Marin-Garcia P, Gallardo-Pino C, Gil-de-Miguel A. Respiratory syncytial virus bronchiolitis in children up to 5 years of age in Spain: epidemiology and comorbidities: an observational study. *Medicine* 2015; **94**(21).
6. Hall CB, Weinberg GA, Iwane MK, et al. The burden of respiratory syncytial virus infection in young children. *N Engl J Med* 2009; **360**(6): 588-98.
7. Iwane MK, Edwards KM, Szilagyi PG, et al. Population-based surveillance for hospitalizations associated with respiratory syncytial virus, influenza virus, and parainfluenza viruses among young children. *Pediatrics* 2004; **113**(6): 1758-64.
8. Muller-Pebody B, Edmunds WJ, Zambon MC, Gay NJ, Crowcroft NS. Contribution of RSV to bronchiolitis and pneumonia-associated hospitalization in English children, April 1995-March 1998. *Epidemiol Infect* 2002; **129**(1): 99-106.
9. Paramore LC, Ciuryla V, Ciesla G, Liu L. Economic impact of respiratory syncytial virus-related illness in the US: an analysis of national databases. *Pharmacoeconomics* 2004; **22**(5): 275-84.
10. Rowlinson E, Dueger E, Taylor T, et al. Incidence and clinical features of respiratory syncytial virus infections in a population-based surveillance site in the Nile Delta Region. *J Infect Dis* 2013; **208 Suppl 3**: S189-96.
11. Vicente D, Montes M, Cilla G, Perez-Yarza EG, Perez-Trallero E. Hospitalization for respiratory syncytial virus in the paediatric population in Spain. *Epidemiol Infect* 2003; **131**(2): 867-72.
12. Nair H, Nokes DJ, Gessner BD, et al. Global burden of acute lower respiratory infections due to respiratory syncytial virus in young children: a systematic review and meta-analysis. *Lancet* 2010; **375**(9725): 1545-55.
13. Nair H, Brooks WA, Katz M, et al. Global burden of respiratory infections due to seasonal influenza in young children: a systematic review and meta-analysis. *Lancet* 2011; **378**(9807): 1917-30.
14. Nair H, Simoes EA, Rudan I, et al. Global and regional burden of hospital admissions for severe acute lower respiratory infections in young children in 2010: a systematic analysis. *Lancet* 2013; **381**(9875): 1380-90.
15. Wu A, Budge PJ, Williams J, et al. Incidence and Risk Factors for Respiratory Syncytial Virus and Human Metapneumovirus Infections among Children in the Remote Highlands of Peru. *PLoS One* 2015; **10**(6).
16. Homaira N, Luby SP, Petri WA, et al. Incidence of respiratory virus-associated pneumonia in urban poor young children of Dhaka, Bangladesh, 2009-2011. *PLoS ONE [Electronic Resource]* 2012; **7**(2): e32056.
17. Hasan K, Jolly P, Marquis G, et al. Viral etiology of pneumonia in a cohort of newborns till 24 months of age in Rural Mirzapur, Bangladesh. *Scand J Infect Dis* 2006; **38**(8): 690-5.
18. Broor S, Parveen S, Bharaj P, et al. A prospective three-year cohort study of the epidemiology and virology of acute respiratory infections of children in rural India. *PLoS ONE [Electronic Resource]* 2007; **2**(6): e491.

19. Sutmoller F, Ferro ZP, Asensi MD, Ferreira V, Mazzei IS, Cunha BL. Etiology of acute respiratory tract infections among children in a combined community and hospital study in Rio de Janeiro. *Clin Infect Dis* 1995; **20**(4): 854-60.
20. Forster J, Ihorst G, Rieger CH, et al. Prospective population-based study of viral lower respiratory tract infections in children under 3 years of age (the PRI.DE study). *Eur J Pediatr* 2004; **163**(12): 709-16.
21. Dede A, Isaacs D, Torzillo PJ, et al. Respiratory syncytial virus infections in Central Australia. *J Paediatr Child Health* 2010; **46**(1-2): 35-9.
22. Ji W, Zhang T, Zhang X, et al. The epidemiology of hospitalized influenza in children, a two year population-based study in the People's Republic of China. *BMC Health Serv Res* 2010; **10**: 82.
23. Fjaerli HO, Farstad T, Bratlid D. Hospitalisations for respiratory syncytial virus bronchiolitis in Akershus, Norway, 1993-2000: a population-based retrospective study. *BMC Pediatr* 2004; **4**(1): 25.
24. Diez Domingo J, Ridao Lopez M, Ubeda Sansano I, Ballester Sanz A. Incidence and cost of hospitalizations for bronchiolitis and respiratory syncytial virus infections in the autonomous community of Valencia in Spain (2001 and 2002). *Anales de Pediatria* 2006; **65**(4): 325-30.
25. Cilla G, Onate E, Perez-Yarza EG, Montes M, Vicente D, Perez-Trallero E. Hospitalization rates for human metapneumovirus infection among 0- to 3-year-olds in Gipuzkoa (Basque Country), Spain. *Epidemiol Infect* 2009; **137**(1): 66-72.
26. Hacimustafaoglu M, Celebi S, Bozdemir SE, et al. RSV frequency in children below 2 years hospitalized for lower respiratory tract infections. *Turk J Pediatr* 2013; **55**(2): 130-9.
27. Stockman LJ, Curns AT, Anderson LJ, Fischer-Langley G. Respiratory syncytial virus-associated hospitalizations among infants and young children in the United States, 1997-2006. *Pediatr Infect Dis J* 2012; **31**(1): 5-9.
28. Weigl JA, Puppe W, Rockahr S, Schmitt HJ. Burden of disease in hospitalized RSV-positive children in Germany. *Klin Padiatr* 2002; **214**(6): 334-42.
29. Deshpande SA, Northern V. The clinical and health economic burden of respiratory syncytial virus disease among children under 2 years of age in a defined geographical area. *Arch Dis Child* 2003; **88**(12): 1065-9.
30. Eriksson M, Bennet R, Rotzen-Ostlund M, von Sydow M, Wirgart BZ. Population-based rates of severe respiratory syncytial virus infection in children with and without risk factors, and outcome in a tertiary care setting. *Acta Paediatr* 2002; **91**(5): 593-8.
31. Resch B, Gusenleitner W, Muller W. The impact of respiratory syncytial virus infection: a prospective study in hospitalized infants younger than 2 years. *Infection* 2002; **30**(4): 193-7.
32. Jansen AGSC, Sanders EAM, Hoes AW, Van Loon AM, Hak E. Influenza- and respiratory syncytial virus-associated mortality and hospitalisations. *Eur Respir J* 2007; **30**(6): 1158-66.
33. Weber MW, Milligan P, Sanneh M, et al. An epidemiological study of RSV infection in the Gambia. *Bull World Health Organ* 2002; **80**(7): 562-8.
34. Suwanjutha S, Sunakorn P, Chantarojanasiri T, et al. Respiratory syncytial virus-associated lower respiratory tract infection in under-5-year-old children in a rural community of central Thailand, a population-based study. *J Med Assoc Thai* 2002; **85 Suppl 4**: S1111-9.
35. Chan PK, Sung RY, Fung KS, et al. Epidemiology of respiratory syncytial virus infection among paediatric patients in Hong Kong: seasonality and disease impact. *Epidemiol Infect* 1999; **123**(2): 257-62.
36. Whitehall JS, Bolisetty S, Whitehall JP, Francis F, Norton R, Patole SK. High rate of indigenous bronchiolitis and palivuzumab. *J Paediatr Child Health* 2001; **37**(4): 416-7.
37. Henrickson KJ, Hoover S, Kehl KS, Hua W. National disease burden of respiratory viruses detected in children by polymerase chain reaction. *Pediatr Infect Dis J* 2004; **23**(1 Suppl): S11-8.
38. Boyce TG, Mellen BG, Mitchel EF, Jr., Wright PF, Griffin MR. Rates of hospitalization for respiratory syncytial virus infection among children in medicaid. *J Pediatr* 2000; **137**(6): 865-70.

39. Holman RC, Curns AT, Cheek JE, et al. Respiratory syncytial virus hospitalizations among American Indian and Alaska Native infants and the general United States infant population. *Pediatrics* 2004; **114**(4): e437-44.
40. Jain S, Williams DJ, Arnold SR, et al. Community-acquired pneumonia requiring hospitalization among U.S. children. *N Engl J Med* 2015; **372**(9): 835-45.
41. Emukule GO, Khagayi S, McMorrow ML, et al. The burden of influenza and rsv among inpatients and outpatients in rural western Kenya, 2009-2012. *PLoS One* 2014; **9**(8).
42. Anders KL, Nguyen HL, Nguyet Minh N, et al. Epidemiology and virology of acute respiratory infections during the first year of life: a birth cohort study in Vietnam. *Pediatr Infect Dis J* 2015; **34**(4): 361-70.
43. Leader S, Kohlhase K. Recent trends in severe respiratory syncytial virus (RSV) among US infants, 1997 to 2000. *J Pediatr* 2003; **143**(5 Suppl): S127-32.
44. Munoz-Quiles C, Lopez-Lacort M, Ubeda-Sansano I, et al. Population-based Analysis of Bronchiolitis Epidemiology in Valencia, Spain. *Pediatric Infectious Disease Journal* 2016; **35**(3): 275-80.
45. Homaira N, Oei JL, Mallitt KA, et al. High burden of RSV hospitalization in very young children: a data linkage study. *Epidemiology and Infection* 2016; **144**(8): 1612-21.
46. Svensson C, Berg K, Sigurs N, Trollfors B. Incidence, risk factors and hospital burden in children under five years of age hospitalised with respiratory syncytial virus infections. *Acta Paediatrica* 2015; **104**(9): 922-6.
47. Moore HC, de Klerk N, Keil AD, et al. Use of data linkage to investigate the aetiology of acute lower respiratory infection hospitalisations in children. *J Paediatr Child Health* 2012; **48**(6): 520-8.
48. Tang LF, Wang TL, Tang HF, Chen ZM. Viral pathogens of acute lower respiratory tract infection in China. *Indian Pediatr* 2008; **45**(12): 971-5.
49. Huang G, Yu D, Mao N, et al. Viral etiology of acute respiratory infection in Gansu Province, China, 2011. *PLoS ONE [Electronic Resource]* 2013; **8**(5): e64254.
50. Zhang Q, Guo Z, MacDonald NE. Vaccine preventable community-acquired pneumonia in hospitalized children in Northwest China. *Pediatr Infect Dis J* 2011; **30**(1): 7-10.
51. Zhang G, Hu Y, Wang H, Zhang L, Bao Y, Zhou X. High incidence of multiple viral infections identified in upper respiratory tract infected children under three years of age in Shanghai, China. *PLoS ONE [Electronic Resource]* 2012; **7**(9): e44568.
52. Chan DCW, Chiu WK, Ip PLS. Respiratory syncytial virus and influenza infections among children <3 years of age with acute respiratory infections in a regional hospital in Hong Kong. *HK J Paediatr* 2007; **12**(1): 15-21+61-2.
53. Sung RY, Chan PK, Tsen T, et al. Identification of viral and atypical bacterial pathogens in children hospitalized with acute respiratory infections in Hong Kong by multiplex PCR assays. *J Med Virol* 2009; **81**(1): 153-9.
54. Chen CJ, Jeng MJ, Yuan HC, Wu KG, Soong WJ, Hwang B. Epidemiology of respiratory syncytial virus in children with lower respiratory tract infection. *Acta Paediatrica Taiwanica* 2005; **46**(2): 72-6.
55. Kaneko M, Watanabe J, Kuwahara M, et al. Impact of respiratory syncytial virus infection as a cause of lower respiratory tract infection in children younger than 3 years of age in Japan. *J Infect* 2002; **44**(4): 240-3.
56. Moriyama Y, Hamada H, Okada M, et al. Distinctive clinical features of human bocavirus in children younger than 2 years. *Eur J Pediatr* 2010; **169**(9): 1087-92.
57. Okada T, Morozumi M, Sakata H, et al. A practical approach estimating etiologic agents using real-time PCR in pediatric inpatients with community-acquired pneumonia. *J Infect Chemother* 2012; **18**(6): 832-40.
58. Harada Y, Kinoshita F, Yoshida LM, et al. Does respiratory virus coinfection increases the clinical severity of acute respiratory infection among children infected with respiratory syncytial virus? *Pediatr Infect Dis J* 2013; **32**(5): 441-5.

59. Yoo SJ, Kuak EY, Shin BM. Detection of 12 respiratory viruses with two-set multiplex reverse transcriptase-PCR assay using a dual priming oligonucleotide system. *Korean J Lab Med* 2007; **27**(6): 420-7.
60. Chung JY, Han TH, Kim SW, Hwang ES. Respiratory picornavirus infections in Korean children with lower respiratory tract infections. *Scand J Infect Dis* 2007; **39**(3): 250-4.
61. Min SJ, Song JS, Choi JH, et al. Analysis of palivizumab prophylaxis in patients with acute lower respiratory tract infection caused by respiratory syncytial virus. [Korean]. *Korean Journal of Pediatric Infectious Diseases* 2011; **18**(2): 154-62.
62. Chan PW, Goh AY, Chua KB, Kharullah NS, Hooi PS. Viral aetiology of lower respiratory tract infection in young Malaysian children. *J Paediatr Child Health* 1999; **35**(3): 287-90.
63. Do AH, van Doorn HR, Nghiem MN, et al. Viral etiologies of acute respiratory infections among hospitalized Vietnamese children in Ho Chi Minh City, 2004-2008. *PLoS ONE [Electronic Resource]* 2011; **6**(3): e18176.
64. Tran DN, Pham TM, Ha MT, et al. Molecular epidemiology and disease severity of human respiratory syncytial virus in Vietnam. *PLoS ONE [Electronic Resource]* 2013; **8**(1): e45436.
65. Yeolekar LR, Damle RG, Kamat AN, Khude MR, Simha V, Pandit AN. Respiratory viruses in acute respiratory tract infections in Western India. *Indian J Pediatr* 2008; **75**(4): 341-5.
66. Mazumdar J, Chawla-Sarkar M, Rajendran K, et al. Burden of respiratory tract infections among paediatric in and out-patient units during 2010-11. *Eur Rev Med Pharmacol Sci* 2013; **17**(6): 802-8.
67. Singh AK, Jain A, Jain B, et al. Viral aetiology of acute lower respiratory tract illness in hospitalised paediatric patients of a tertiary hospital: One year prospective study. *Indian J Med Microbiol* 2014; **32**(1): 13-8.
68. Ekalaksananan T, Pientong C, Kongyingyoes B, Pairojkul S, Teeratakulpisarn J, Heng S. Etiology of acute lower respiratory tract infection in children at Srinagarind Hospital, Khon Kaen, Thailand. *Southeast Asian J Trop Med Public Health* 2001; **32**(3): 513-9.
69. Siritantikorn S, Puthavathana P, Suwanjutha S, et al. Acute viral lower respiratory infections in children in a rural community in Thailand. *J Med Assoc Thai* 2002; **85 Suppl 4**: S1167-75.
70. Teeratakulpisarn J, Ekalaksananan T, Pientong C, Limwattananon C. Human metapneumovirus and respiratory syncytial virus detection in young children with acute bronchiolitis. *Asian Pac J Allergy Immunol* 2007; **25**(2-3): 139-45.
71. Turner P, Turner C, Watthanaworawit W, et al. Respiratory virus surveillance in hospitalised pneumonia patients on the Thailand-Myanmar border. *BMC Infect Dis* 2013; **13**(1).
72. Khamis FA, Al-Kobaisi MF, Al-Areimi WS, Al-Kindi H, Al-Zakwani I. Epidemiology of respiratory virus infections among infants and young children admitted to hospital in Oman. *J Med Virol* 2012; **84**(8): 1323-9.
73. Bdour S. Respiratory syncytial virus subgroup A in hospitalized children in Zarqa, Jordan. *Ann Trop Paediatr* 2001; **21**(3): 253-61.
74. Al-Toum R, Bdour S, Ayyash H. Epidemiology and clinical characteristics of respiratory syncytial virus infections in Jordan. *J Trop Pediatr* 2006; **52**(4): 282-7.
75. Bakir TM, Halawani M, Ramia S. Viral aetiology and epidemiology of acute respiratory infections in hospitalized Saudi children. *J Trop Pediatr* 1998; **44**(2): 100-3.
76. Bukhari EE, Elhazmi MM. Viral agents causing acute lower respiratory tract infections in hospitalized children at a tertiary care center in Saudi Arabia. *Saudi Med J* 2013; **34**(11): 1151-5.
77. Al-Shehri MA, Sadeq A, Quli K. Bronchiolitis in Abha, Southwest Saudi Arabia: viral etiology and predictors for hospital admission. *West Afr J Med* 2005; **24**(4): 299-304.
78. Mlinaric-Galinovic G, Vilibic-Cavlek T, Ljubin-Sternak S, et al. Eleven consecutive years of respiratory syncytial virus outbreaks in Croatia. *Pediatr Int* 2009; **51**(2): 237-40.
79. Mlinaric-Galinovic G, Bace A, Cepin-Bogovic J, Ivkovic-Jurekovic I, Sim R, Cosic M. Characteristics of 2006/2007 respiratory syncytial virus outbreak in Zagreb County: proof of predictability of outbreaks periodicity. *Paediatria Croatica* 2009; **53**(2): 49-52.

80. Juven T, Mertsola J, Waris M, et al. Etiology of community-acquired pneumonia in 254 hospitalized children. *Pediatr Infect Dis J* 2000; **19**(4): 293-8.
81. Foulongne V, Guyon G, Rodiere M, Segondy M. Human metapneumovirus infection in young children hospitalized with respiratory tract disease. *Pediatr Infect Dis J* 2006; **25**(4): 354-9.
82. Weigl JA, Puppe W, Schmitt HJ. Seasonality of respiratory syncytial virus-positive hospitalizations in children in Kiel, Germany, over a 7-year period. *Infection* 2002; **30**(4): 186-92.
83. Wasem S, Weichert S, Walther S, et al. Lower respiratory tract disease in children: constant pathogens - constant management?! *Klin Padiatr* 2008; **220**(5): 291-5.
84. Vagia F, Makri A, Iliadou H, et al. Prevalence and clinical features of respiratory syncytial virus and human metapneumovirus lower respiratory disease among children in Greece. *Clin Microbiol Infect* 2010; **16**: S428-S9.
85. Wolf DG, Greenberg D, Kalkstein D, et al. Comparison of human metapneumovirus, respiratory syncytial virus and influenza A virus lower respiratory tract infections in hospitalized young children. *Pediatr Infect Dis J* 2006; **25**(4): 320-4.
86. Wolf DG, Greenberg D, Shemer-Avni Y, Givon-Lavi N, Bar-Ziv J, Dagan R. Association of human metapneumovirus with radiologically diagnosed community-acquired alveolar pneumonia in young children. *J Pediatr* 2010; **156**(1): 115-20.
87. Maggi F, Andreoli E, Pifferi M, Meschi S, Rocchi J, Bendinelli M. Human bocavirus in Italian patients with respiratory diseases. *J Clin Virol* 2007; **38**(4): 321-5.
88. Canducci F, Debiaggi M, Sampaolo M, et al. Two-year prospective study of single infections and co-infections by respiratory syncytial virus and viruses identified recently in infants with acute respiratory disease. *J Med Virol* 2008; **80**(4): 716-23.
89. Zuccotti G, Dilillo D, Zappa A, et al. Epidemiological and clinical features of respiratory viral infections in hospitalized children during the circulation of influenza virus A(H1N1) 2009. *Influenza Other Respi Viruses* 2011; **5**(6): e528-34.
90. Van Leeuwen JC, Goossens LK, Hendrix RM, Van Der Palen J, Lusthusz A, Thio BJ. Equal virulence of rhinovirus and respiratory syncytial virus in infants hospitalized for lower respiratory tract infection. *Pediatr Infect Dis J* 2012; **31**(1): 84-6.
91. Hervas D, Reina J, Yanez A, del Valle JM, Figuerola J, Hervas JA. Epidemiology of hospitalization for acute bronchiolitis in children: differences between RSV and non-RSV bronchiolitis. *Eur J Clin Microbiol Infect Dis* 2012; **31**(8): 1975-81.
92. Garcia-Garcia ML, Calvo C, Perez-Brena P, De Cea JM, Acosta B, Casas I. Prevalence and clinical characteristics of human metapneumovirus infections in hospitalized infants in Spain. *Pediatr Pulmonol* 2006; **41**(9): 863-71.
93. Calvo C, Pozo F, Garcia-Garcia ML, et al. Detection of new respiratory viruses in hospitalized infants with bronchiolitis: a three-year prospective study. *Acta Paediatr* 2010; **99**(6): 883-7.
94. Jansson L, Nilsson P, Olsson M. Socioeconomic environmental factors and hospitalization for acute bronchiolitis during infancy. *Acta Paediatr* 2002; **91**(3): 335-8.
95. Cevey-Macherel M, Galetto-Lacour A, Gervaix A, et al. Etiology of community-acquired pneumonia in hospitalized children based on WHO clinical guidelines. *Eur J Pediatr* 2009; **168**(12): 1429-36.
96. Turkish Neonatal S. The seasonal variations of respiratory syncytial virus infections in Turkey: a 2-year epidemiological study. *Turk J Pediatr* 2012; **54**(3): 216-22.
97. Carballal G, Videla C, Sequeira MD, Mistchenko A, Requeijo PV, Arbiza J. Respiratory syncytial virus: changes in prevalence of subgroups A and B among Argentinian children, 1990-1996. *J Med Virol* 2000; **61**(2): 275-9.
98. Viegas M. Molecular epidemiology of respiratory syncytial virus in pediatric patients in a six-year period. *Acta Bioquimica Clinica Latinoamericana* 2011; **45**(1): 3-45.
99. Macedo SEC, Menezes AMB, Post P, Albernaz E, Knorst M. Respiratory syncytial virus infection in children under one year of age hospitalized for acute respiratory diseases in Pelotas, RS. *J pneumol* 2002; **29**(1): 4-8.

100. Moura FEA, Ribeiro DH, Borges LC, Ramos EAG. Laboratory diagnosis of acute respiratory infection of viral origin [Portuguese]. *Pediatria Moderna* 2002; **38**(6): 255-60.
101. Vieira SE, Gilio AE, Durigon EL, Ejzenberg B. Lower respiratory tract infection caused by respiratory syncytial virus in infants: the role played by specific antibodies. *Clinics (Sao Paulo, Brazil)* 2007; **62**(6): 709-16.
102. Oliveira DB, Durigon EL, Carvalho AC, et al. Epidemiology and genetic variability of human metapneumovirus during a 4-year-long study in Southeastern Brazil. *J Med Virol* 2009; **81**(5): 915-21.
103. Ferronato AE, Gilio AE, Ferraro AA, Paulis M, Vieira SE. Etiological diagnosis reduces the use of antibiotics in infants with bronchiolitis. *Clinics (Sao Paulo, Brazil)* 2012; **67**(9): 1001-6.
104. Nascimento-Carvalho CM, Cardoso MR, Barral A, et al. Seasonal patterns of viral and bacterial infections among children hospitalized with community-acquired pneumonia in a tropical region. *Scand J Infect Dis* 2010; **42**(11-12): 839-44.
105. Salomao Jr JB, Gardinassi LGA, Simas PVM, et al. Human respiratory syncytial virus in children hospitalized for acute lower respiratory infection [Portuguese] (Virus respiratorio sincicial humano em criancas hospitalizadas por infeccoes agudas das vias aereas inferiores). *J Pediatr (Rio J)* 2011; **87**(3): 219-24.
106. Pecchini R, Berezin EN, Felicio MC, et al. Incidence and clinical characteristics of the infection by the respiratory syncytial virus in children admitted in Santa Casa de Sao Paulo Hospital. *Braz J Infect Dis* 2008; **12**(6): 476-9.
107. Ferone EA, Berezin EN, Durigon GS, et al. Clinical and epidemiological aspects related to the detection of adenovirus or respiratory syncytial virus in infants hospitalized for acute lower respiratory tract infection. *J Pediatr (Rio J)* 2014; **90**(1): 42-9.
108. Lamarao LM, Ramos FL, Mello WA, et al. Prevalence and clinical features of respiratory syncytial virus in children hospitalized for community-acquired pneumonia in northern Brazil. *BMC Infect Dis* 2012; **12**: 119.
109. Sparremberger DAH, Luisi F, Azevedo AV, et al. Epidemiological surveillance and influence of co-infection by respiratory virusesin the severity of acute bronchiolitis in infants (Características epidemiológicas e influência da coinfecção por vírus respiratórios na gravidade da bronquiolite aguda em lactentes). *Sci med* 2011; **21**(3).
110. Banerji A, Greenberg D, White LF, et al. Risk factors and viruses associated with hospitalization due to lower respiratory tract infections in Canadian Inuit children : a case-control study. *Pediatr Infect Dis J* 2009; **28**(8): 697-701.
111. Santibanez P, Gooch K, Vo P, Lorimer M, Sandino Y. Acute care utilization due to hospitalizations for pediatric lower respiratory tract infections in British Columbia, Canada. *BMC Health Serv Res* 2012; **12**: 451.
112. Bedoya VI, Abad V, Trujillo H. Frequency of respiratory syncytial virus in hospitalized infants with lower acute respiratory tract infection in Colombia. *Pediatr Infect Dis J* 1996; **15**(12): 1123-4.
113. Pineros JG, Baquero H, Bastidas J, et al. Respiratory syncytial virus infection as a cause of hospitalization in population under 1 year in Colombia. *J Pediatr (Rio J)* 2013; **89**(6): 544-8.
114. Noyola DE, Zuviri-Gonzalez A, Castro-Garcia JA, Ochoa-Zavala JR. Impact of respiratory syncytial virus on hospital admissions in children younger than 3 years of age. *J Infect* 2007; **54**(2): 180-4.
115. Singleton RJ, Petersen KM, Berner JE, et al. Hospitalizations for respiratory syncytial virus infection in Alaska Native children. *Pediatr Infect Dis J* 1995; **14**(1): 26-30.
116. Flaherman V, Li S, Ragins A, Masaquel A, Kipnis P, Escobar GJ. Respiratory syncytial virus testing during bronchiolitis episodes of care in an integrated health care delivery system: a retrospective cohort study. *Clin Ther* 2010; **32**(13): 2220-9.
117. Garcia CG, Bhore R, Soriano-Fallas A, et al. Risk factors in children hospitalized with RSV bronchiolitis versus non-RSV bronchiolitis. *Pediatrics* 2010; **126**(6): e1453-60.
118. Suryadevara M, Cummings E, Bonville CA, et al. Viral etiology of acute febrile respiratory illnesses in hospitalized children younger than 24 months. *Clin Pediatr (Phila)* 2011; **50**(6): 513-7.

119. Kwofie TB, Anane YA, Nkrumah B, Annan A, Nguah SB, Owusu M. Respiratory viruses in children hospitalized for acute lower respiratory tract infection in Ghana. *Virol J* 2012; **9**: 78.
120. Feikin DR, Njenga MK, Bigogo G, et al. Viral and bacterial causes of severe acute respiratory illness among children aged less than 5 years in a high malaria prevalence area of western Kenya, 2007-2010. *Pediatr Infect Dis J* 2013; **32**(1): e14-9.
121. Hammitt LL, Kazungu S, Morpeth SC, et al. A preliminary study of pneumonia etiology among hospitalized children in Kenya. *Clin Infect Dis* 2012; **54**(SUPPL. 2): S190-S9.
122. Fuller JA, Njenga MK, Bigogo G, et al. Association of the CT values of real-time PCR of viral upper respiratory tract infection with clinical severity, Kenya. *J Med Virol* 2013; **85**(5): 924-32.
123. Avendano LF, Palomino MA, Larranaga C. Surveillance for respiratory syncytial virus in infants hospitalized for acute lower respiratory infection in Chile (1989 to 2000). *J Clin Microbiol* 2003; **41**(10): 4879-82.
124. Hussey GD, Apolles P, Arendse Z, et al. Respiratory syncytial virus infection in children hospitalised with acute lower respiratory tract infection. *S Afr Med J* 2000; **90**(5): 509-12.
125. Loscertales MP, Roca A, Ventura PJ, et al. Epidemiology and clinical presentation of respiratory syncytial virus infection in a rural area of southern Mozambique. *Pediatr Infect Dis J* 2002; **21**(2): 148-55.
126. Tsolia MN, Kafetzis D, Danelatou K, et al. Epidemiology of respiratory syncytial virus bronchiolitis in hospitalized infants in Greece. *Eur J Epidemiol* 2003; **18**(1): 55-61.
127. Videla C, Carballal G, Misirlian A, Aguilar M. Acute lower respiratory infections due to respiratory syncytial virus and adenovirus among hospitalized children from Argentina. *Clin Diagn Virol* 1998; **10**(1): 17-23.
128. Cao L, Lu J, Qian Y, et al. A viral etiological and clinical investigation into acute lower respiratory tract infection in children under 6 years old [Chinese] *Chin J Pract Pediatr* 2004; **19**(9): 528-31.
129. Liu CY, Xiao Y, Xie ZD, et al. Viral etiology of acute respiratory tract infection among pediatric inpatients and outpatients from 2010 to 2012 in Beijing, China [Chinese]. *Chin J Pediatr* 2013; **51**(4): 255-9.
130. Ding XF, Zhang B, Zhong LL, et al. Viral etiology and risk factors for severe community-acquired pneumonia in children [Chinese]. *J Clin Pediatr* 2012; **30**(9): 857-61.
131. Xiao NG, Zhang B, Duan ZJ, et al. Viral etiology of 1165 hospitalized children with acute lower respiratory tract infection [Chinese]. *Chin J Contemp Pediatr* 2012; **14**(1): 28-32.
132. Li LP, Lai Y, Yi SS, SUn KS, Zhu CH. Pathogenic analysis of acute respiratory infections among children in Changsha area [Chinese] *Pract Prev Med* 2013; **20**(7): 876-8.
133. Xie H, Li GC, Wang PP, et al. An etiology investigation of acute lower respiratory tract infection in children in Chaozhou [Chinese] *Med Innov China* 2013; **10**(11): 116-8.
134. Zhang L. Analysis of Etiological Agent in Hospitalized Children with Lower Respiratory infection in Cheng Du Area [Chinese] [Master]: Luzhou Medical College; 2008.
135. Cao Y, Zhang M, Liao H, et al. Surveillance on 592 cases of human respiratory syncytial virus infection in hospitalized children with respiratory tract infection in Sichuan [Chinese]. *Mod Prev Med* 2013; **40**(7): 1249-51.
136. Shi WY, Zhu WH, He ZG, Xu GZ, Li LP. Analysis of characteristics of pathogens in viral pneumonia [Chinese]. *Med Inf* 2012; **25**(3): 93.
137. Peng DH, Liu E, M., Zhao XD, Huang Y, Liu Y, Luo XJ. Surveillance for respiratory viruses in children with acute lower respiratory infections in Chongqing between 2003 and 2007 [Chinese]. *J Appl Clin Pediatr* 2009; **24**(10): 768-70.
138. Du LN. Molecular epidemiology of respiratory syncytial virus and human metapneumovirus in children in Chongqing area [Chinese] [Master]: Chongqing Medical University; 2012.
139. Zhu Y, Hua ZY. Clinical and epidemiological features of respiratory syncytial virus penumonia in 182 neonates [Chinese] *J Appl Clin Pediatr* 2011; **26**(22): 1719-21.

140. Lei XY. Epidemiological characteristics of children's respiratory tract viral infection in Chongqing during 2009 and 2011 [Chinese] [Master]: Chongqing Medical Univesity; 2012.
141. Lu XM, Li SP, He YJ, Peng Q, Liu SJ. Analysis on detective resutls of multiple respiratory virus antigens in 1256 children [Chinese]. *J Appl Clin Pediatr* 2012; **27**(22): 1733-5.
142. Wang SM. Analysis of pathogens in children pneumonia [Chinese]. *Med Inf* 2011; (09): 4262.
143. Jiang JP, Lan YL. Study of respiratory syncytial virus pneumonia in neonate [Chinese]. *Mod J Integr Tradit Chin West Med* 2007; **16**(14): 1892-3.
144. Xie YP, Deng L. 1815 cases of lower respiratory infection in young children [Chinese]. *Guangzhou Med J* 2011; **42**(3): 24-6.
145. Yin WY, Lu G, Su SS, Wang F. An analysis on respiratory seven virus detection results in sputum in 1050 children with capillary bronchitis [Chinese]. *J Guiyang Med Coll* 2012; **37**(3): 280-2.
146. Huang L, Zhou Y, Wang Y. Epidemic characteristics of common virus causing lower respiratory track infection of children from Guiyang area [Chinese]. *Matern Child Health Care China* 2013; **28**(26): 4307-9.
147. Tan ZT, Shi L, Lu GX, et al. Detection of respiratory syncytial virus in children with respiratory tract infections by nucleic acid amplification fluorogenic quantitative assay [Chinese]. *Chin J Contemp Pediatr* 2009; **11**(10): 825-8.
148. Ye LF, Yuan CD. A study on etiology of acute lower respiratory tract infections in infants [Chinese] *Zhejiang Prev Med* 2004; **16**(8): 5-6.
149. Ma XL, Xu YC, Zheng JY, Chen XJ. Study on pathogen and clinical characteristics of infectious pneumonia in neonates [Chinese]. *Zhejiang Prev Med* 2005; **17**(1): 6-8.
150. Miao Z, Xu Y, Li P. Human metapneumovirus infection of hospitalized infants in Jiaxing District [Chinese]. *Chin J Nosocomiol* 2010; **20**(24): 3877-9.
151. Zhang AL, Wu M, Zhang X, Wu XY. Results of common virus test in hospitalised children [Chinese] *Zhejiang Prev Med* 2013; **25**(4): 62-4.
152. Fan M, Wu Q, Ni LX, Song SQ, Feng XX. Analysis of viral pathogens identified in children with lower respiratory infection in Kunming area [Chinese]. *Chin J Child Health Care* 2007; **15**(5): 539-41.
153. Zheng WJ. A study on etiology and clinial epidemiology of respiratory tract in children in Kunming area [Chinese] [Master]: Kunming Medical University; 2011.
154. Zhang B, Wang X, Zhang W, Chen XY. Epidemiological features of acute lower respiratory tract viral infections in children [Chinese] *Zhejiang Med J* 2012; **34**(4): 250-5.
155. Chen LF, Chen LP. Investigation on respiratory syncytial virus of lower respiratory tract infection in the third liaocheng peoples hospital [Chinese]. *Chin J Coal Ind Med* 2009; **12**(11): 1678-80.
156. Chen J. Study on viral pathogen of bronchiolitis in two hundred and six infants [Chinese] [Master]: Shandong University; 2012.
157. Qin M, Tian M, Xia W, Wang HY, Shi SY, Chen Q. Etiology of community-acquired pneumonia in children [Chinese]. *J Clin Pediatr* 2008; **26**(4): 312-5.
158. Chen Q, Shi SY, Hu Z, Zhang QH, Cao X. Detection of Mycoplasm pneumonia, Chlamydia trachomatis and common respiratory viruses in children with acute respiratory infection in Nanjing [Chinese]. *Chin J Contemp Pediatr* 2010; **12**(6): 450-4.
159. Jiang M, Zhao YF, Zhang YH, et al. Etiological analysis of childen with acute respiratory infection in Nanjing [Chinese] *Chin J Health Lab Technol* 2013; **23**(4): 932-6.
160. Zhao GC, Wang XH, Zhu QR. Etiology and clinical epidemiology of children with acute pneumonia in Shanghai area [Chinese]. *Chin J Infec Chemother* 2003; **3**(3): 134-7.
161. Che DD, Lu Q, Lu M, Ji F, Tong HY. Analysis of pathogens in children with acute lower respiratory infection in Shanghai in 2000 [Chinese]. *Chin J Contemp Pediatr* 2004; **6**(2): 136-8.
162. Lu M, Zeng M, Lu Q, Gu LQ. The epidemiologic feature of viral infection in the children with acute lower respiratory tract infections in Shanghai [Chinese] *Chin J Infec Chemother* 2005; **5**(3): 152-5.

163. Zeng M, Wang XH, Yu H, Zhu QR. Epidemiological characteristics of common respiratory viruses among children with acute respiratory tract infection in Shanghai [Chinese]. *Chin J Infect Dis* 2008; **26**(9): 527-33.
164. Zhao B, Shen J, Gao Y, Yu X, Zhang X, Wu F. Detection for respiratory viruses in Shanghai with multiplex PCR from 2009 to 2010 [Chinese]. *J Hyg Res* 2011; **40**(5): 635-7.
165. Zhang XQ, Hu J, Ning XX, Gao SF, Wang L. Analysis on the detection situation of 7 common viruses in 2425 children with respiratory tract infection [Chinese] *Lab Med* 2013; **28**(7): 602-5.
166. Ru GP, Li YY, Wang Q, Wang HJ, Zhao WJ. Etiologic analysis of 1200 children less 5 years old with lower respiratory tract infections in Shangzhou City [Chinese] *Hebei Med* 2013; **19**(8): 1243-5.
167. Ou SY, Lin GY, Wu Y, Lu XD, Lin CX, Zhou RB. Viral pathogens of acute lower respiratory tract infection in hospitalized children from East Guangdong of China [Chinese]. *Chin J Contemp Pediatr* 2009; **11**(3): 203-6.
168. Ji W, Chen ZR, Zhou WF, et al. Etiology of acute respiratory tract infection in hospitalized children Suzhou from 2005 to 2011 [Chinese]. *Chin J Prev Med* 2013; **47**(6): 497-503.
169. Sun LX, Huang JF, Zhang H. Study of viral pathogens from children with lower respiratory infection in Tianjin [Chinese]. *China J Mod Med* 2004; **14**(24): 129-30.
170. Wang W, Lin SX, Li SY, ZHang H, Huang JF. Analysis of viral pathogens of children with acute respiratory infection in Tianjin area [Chinese]. *Tianjin Med J* 2012; **40**(6): 625-7.
171. Yang WM, Sun ZH, Zhang SP, Ma HM, Liu CY. Diagnosis of virus in young children with acute lower respiratory infection in Weifang area [Chinese]. *Chin J Child Health Care* 2009; **17**(2): 233-5.
172. Xiang QW, Luo YC, Chen XF. Investigation on the viral etiology of acute lower respiratory tract infection in Wenzhou Yuying children's hospital [Chinese]. *Chin J Pract Pediatr* 2005; **20**(12).
173. Wan C, Yan SN, Zheng G. Clinial features of 112 cases of respiratory syncytial virus associated with lower respiratory infection [Chinese]. *Zhenjiang Clin Med* 2006; **8**(4): 396-7.
174. Cao SY, Sun ZX, Chen XF, Cai XH, Yang SY. Detecting result on rspirovirus and chlamydia pneumoniae of pnemonia from nasopharyngeal secretions in infants [Chinese] *Chin J Child Health Care* 2007; **15**(4): 368-70.
175. Chang J, Li CY, Li HJ, Luo YC, Chen XF, Yang SY. Viral etiology of acute respiratory infection in children from Wenzhou between 2007 and 2008 [Chinese]. *Chin J Contemp Pediatr* 2010; **12**(1): 32-4.
176. Wang FM, Luo YC, Zhu YY, Xu LD. A study of viral etiology of impatients below 5 years old with community acquired pneumonia in Wenzhou [Chinese]. *Zhejiang Prev Med* 2011; **23**(2): 17-9.
177. Liang Y, Wang Y, Nan Y, Chen SQ. Analysis of 75 cases lower respiratory tract infection by respiratory syncytial virus [Chinese] *Chin J Perinat Med* 2012; **15**(4): 206-10.
178. Zhang YM, Feng YZ, Luo SZ, Lei CL. Study on viral pathogens of lower respiratory infection in young children [Chinese]. *Shanxi Med J* 2002; **31**(3): 195-7.
179. Ju HX, Zhou ZW, Zhu R, Tang JF. Viral etiology of hospitalized children with acute respiratory tract infection [Chinese] *J Jiangsu Uni* 2012; **22**(6): 494-6.
180. Liu XT, Wang GL, Luo XF, et al. Spectrum of pathogens for community-acquired pneumonia in children [Chinese]. *Chin J Contemp Pediatr* 2013; **15**(1): 42-5.
181. Jiang ZM, Peng J, Gu M, Liu JQ, Ji Q. Analysis on pathogens in respiratory infection in 1410 children [Chinese] *Chin J Infec control* 2013; **12**(2): 129-31.
182. Malekshahi SS, Azad TM, Yavarian J, Shahmahmoodi S, Naseri M, Rezaei F. Molecular detection of respiratory viruses in clinical specimens from children with acute respiratory disease in Iran. *Pediatr Infect Dis J* 2010; **29**(10): 931-3.
183. Faghihloo E, Yavarian J, Jandaghi NZS, Shadab A, Azad TM. Genotype circulation pattern of human respiratory syncytial virus in Iran. *Infect Genet Evol* 2014; **22**: 130-3.
184. Zhang G, Xia X, Lyu L. Analysis on the detection results of common viruses in 503 children with acute respiratory tract infection [Chinese]. *Zhongguo Weishengtaxixue Zazhi / Chinese Journal of Microecology* 2014; **26**(10): 1206-8.

185. Xu L, Qiu C, Gong P, Tang J, Deng J. Pathogen spectrum of respiratory viruses in children of Shaoguan, 2010-2012 [Chinese]. *J Trop Med* 2014; **14**(1): 124-6.
186. He Y. Analysis of features of children with viral infection from outpatiens and inpatients during 2011 and 2012 [Chinese]. *Matern Child Health Care China* 2015; **30**: 382-4.
187. Liang L, Ge RH. Analysis of diagnosis of children with acute lower respiratory infection in Guiyang area during 2012 and 2013 [Chinese]. *Guiyang Med* 2014; **38**(7): 655-7.
188. Cao XY, Chen WQ, Luo KM, Li GH, Huang HX. Investigation on respiratory syncytial virus infection in children in Qingyuan District from 2012-2014 [Chinese]. *Occup Health* 2015; **31**(10).
189. Chen Y, Lian GW, Zhang YY, et al. Etiology of community-acquired pneumonia among pediatric inpatients in Guangzhou during 2012 and 2013 [Chinese]. *Chinese Journal of Clinical Infectious Disease* 2014; **7**(6): 521-5.
190. Zhang H, Yu X. Viral etiology analysis of 3496 hospitalized children with lower respiratory tract infection [Chinese]. *Mod Prev Med* 2015; **42**(3): 437-9.
191. Yao J, Shen GS, Fan LH, Li WW. Analysis of viral pathogens among 6089 children hospitalised with respiratory infection [Chinese]. *Chin J Epidemiol* 2015; **36**(6): 664-6.
192. Zhang QL, Zhong BC, Tang YM. Analysis of viruses in children with acute respiratory infection in Sanshui area [Chinese]. *Laboratory Medince and Clinic* 2014; **11**(14): 1966-70.
193. Hu XW, Wang WP. Epidemiological characteristics of respiratory viruses in children of Wuhan [Chinese]. *Lab Med Clin* 2014; **11**(6): 770-2.
194. Ye YY, Li WC, Zhang N. Analysis of 374 hospitalised cases of respiratory infection [Chinese]. *Chin J Rural Med Pharm* 2014; **21**(21): 60-1.
195. Xue HF, Liu XP, Su WD. Analysis of epidemiology of 2813 cases with acute respiratory infection in children [Chinese]. *Zhejiang J Integr Tradit Chin West Med* 2014; **24**(9): 834-6.
196. Wang D, Yang J, Wang B, et al. Etiological analysis of children with respiratory tract viral infection in Dazu district [Chinese]. *Lab Med Clin* 2015; **12**(10): 1364-6.
197. Ding PB, Tian PJ. Analysis of viral pathogens in young children with lower respiratory infection in Ningxia district [Chinese]. *Contemp Med* 2014; **20**(20): 159-60.
198. Zhang YL, Lin HL, Lan JH, Qian LJ. Analysis of children hospitalised with respiratory syncytial virus associated with acute lower respiratory infection [Chinese] *Chin Pediatr Emerg Med* 2014; **21**(3): 143-4.
199. Sheng KH, Xu MY, Sun BL, Huang ZH. The aetiological analysis of common viruses of acute lower respiratory infection among the hospitalised children 0 to 7 years old in Nantong of Jiangsu [Chinese]. *Chin J Prim Med Pharm* 2014; **21**(11): 1607-9.
200. Lin ZB, Li LQ, Chen QH, Zheng JY, Fu QL. Viral etiology investigation of children community acquired pneumonia in Quanzhou area [Chinese]. *Chin Community Dr* 2015; **31**(4): 102-5.
201. Yin F. The viral epidemiology and the analysis of clinical features of human bocavirus in children with acute respiratory tract infection in Sochow area [Chinese] [Master]: Soochow University; 2014.
202. Liu Q, Zhang B, Xie ZP, et al. Etiological analysis of chidlren hospitalised with acute lower respiratory infection in Changsha area [Chinese]. *J Hunan Normal Univ* 2015; **12**(1): 26-31.
203. He Y, Lin G, Wang Q, et al. A 3-year prospective study of the epidemiology of acute respiratory viral infections in hospitalized children in Shenzhen, China. *Influenza Other Respi Viruses* 2014; **8**(4): 443-51.
204. Takeyama A, Hashimoto K, Sato M, et al. Clinical and epidemiologic factors related to subsequent wheezing after virus-induced lower respiratory tract infections in hospitalized pediatric patients younger than 3 years. *Eur J Pediatr* 2014; **173**(7): 959-66.
205. Hamada H, Ogura A, Hotta C, Wakui T, Ogawa T, Terai M. Epidemiological study of respiratory viruses detected in patients under two years old who required admission because of lower respiratory disease. *Kansenshogaku Zasshi - Journal of the Japanese Association for Infectious Diseases* 2014; **88**(4): 423-9.

206. Tallo VL, Kamigaki T, Tan AG, et al. Estimating influenza outpatients' and inpatients' incidences from 2009 to 2011 in a tropical urban setting in the Philippines. *Influenza Other Respi Viruses* 2014; **8**(2): 159-68.
207. Balmaks R, Ribakova I, Gardovska D, Kazaks A. Molecular epidemiology of human respiratory syncytial virus over three consecutive seasons in Latvia. *J Med Virol* 2014; **86**(11): 1971-82.
208. Farzin A, Saha SK, Baqui AH, et al. Population-based incidence and etiology of community-acquired neonatal viral infections in Bangladesh: a community-based and hospital-based surveillance study. *Pediatr Infect Dis J* 2015; **34**(7): 706-11.
209. Assaf-Casals A, Ghanem S, Rajab M. Respiratory syncytial virus: prevalence and features among hospitalized Lebanese children. *Br J Med Med Res* 2015; **6**(1): 77-87.
210. Murray J, Bottle A, Sharland M, et al. Risk factors for hospital admission with RSV bronchiolitis in England: a population-based birth cohort study. *PLoS One* 2014; **9**(2).
211. Pourakbari B, Mahmoudi S, Movahedi Z, et al. Viral etiology of acute lower respiratory tract infections in hospitalized young children in a children's referral hospital in Iran. *Turk J Pediatr* 2014; **56**(4): 354-9.
212. Chen YW, Huang YC, Ho TH, Huang CG, Tsao KC, Lin TY. Viral etiology of bronchiolitis among pediatric inpatients in northern Taiwan with emphasis on newly identified respiratory viruses. *Journal of Microbiology, Immunology & Infection* 2014; **47**(2): 116-21.
213. Ouedraogo S, Traore B, Nene Bi ZA, et al. Viral etiology of respiratory tract infections in children at the pediatric hospital in Ouagadougou (Burkina Faso). *PLoS ONE [Electronic Resource]* 2014; **9**(10): e110435.
214. Dereci S, Cicek AC, Ozkasap S, Mutlu MA, Kocyigit S, Sahin K. Distribution of respiratory viruses which cause lower respiratory tract infection in pediatric age group. *Journal of Coastal Life Medicine* 2015; **3**(7): 547-50.
215. Parsania M, Poopak B, Pouriayevali MH, Haghighi S, Amirkhani A, Nateghian A. Detection of human Metapneumovirus and Respiratory syncytial virus by real-time polymerase chain reaction among hospitalized young children in Iran. *Jundishapur Journal of Microbiology* 2016; **9**(3).
216. Amer HM, Alshaman MS, Farrag MA, Hamad ME, Alsaadi MM, Almajhdi FN. Epidemiology of 11 respiratory RNA viruses in a cohort of hospitalized children in Riyadh, Saudi Arabia. *Journal of Medical Virology* 2016; **88**(6): 1086-91.
217. Kenmoe S, Tchendjou P, Vernet MA, et al. Viral etiology of severe acute respiratory infections in hospitalized children in Cameroon, 2011-2013. *Influenza and other Respiratory Viruses* 2016; **10**(5): 386-93.
218. Oliveira-Santos M, Santos JA, Soares J, Dias A, Quaresma M. Influence of meteorological conditions on RSV infection in Portugal. *International Journal of Biometeorology* 2016; **60**(12): 1807-17.
219. Richter J, Panayiotou C, Tryfonos C, et al. Aetiology of Acute Respiratory Tract Infections in Hospitalised Children in Cyprus. *Plos One* 2016; **11**(1).
220. Wang H, Zheng Y, Deng J, et al. Prevalence of respiratory viruses among children hospitalized from respiratory infections in Shenzhen, China. *Virology Journal* 2016; **13**(39).
221. Pavic-Espinoza I, Bendezu-Medina S, Herrera-Alzamora A, et al. High prevalence of Bordetella pertussis in children under 5 years old hospitalized with acute respiratory infections in Lima, Peru. *BMC Infectious Diseases* 2015; **15**: 554.
222. Pratheepamornkull T, Ratanakorn W, Samransamruajkit R, Poovorawan Y. Causative Agents of Severe Community Acquired Viral Pneumonia among Children in Eastern Thailand. *Southeast Asian Journal of Tropical Medicine & Public Health* 2015; **46**(4): 650-6.
223. Qiao RJ, Wang P, Kang Q, et al. Epidemiological analysis of respiratory syncytial virus in pediatric patients under 5 years in hospital with pneumonia in Baiyin District. *Chinese J Exp Clin Virol* 2015; **29**(6): 510-4.
224. Wang YK, Gao WJ, An SH, An HB, Ge SW, Zhang WC. Detection of 7 viral respiratory infection among children. *Hebei Medical Journal* 2016; **38**(14): 2229-31.

225. Xie JH, Hua L, Zhong JY, et al. Epidemiological features of respiratory syncytial virus infection in Guangzhou children, 2011 to 2013. *Chin J Biomed Eng* 2015; **21**(3): 270-5.
226. Chen H, Dong L. Surveilance of common viruse causing acute respiratory infection in hospitalised children in Wenzhou city. *Zhejiang Prev Med* 2015; **27**(11): 1134-6.
227. Wu YQ. Detection and analysis of the virus in 1200 cases children with acute respiratory tract infection. *China Journal of Child Health Care* 2015; **23**(11): 1216-8.
228. Peng Y, Shu C, Fu Z, Li QB, Liu Z, Yan L. Pathogen detection of 1613 cases of hospitalized children with community acquired pneumonia. *Chin J Contemp Pediatr* 2015; **17**(11): 1193-9.
229. Hu J, Zhao K, Zhu T. Retrospective study of viral etiology of acute lower respiratory tract infections in hospitalised children in Suzhou Area. *Heilongjiang Medical Journal* 2015; **39**(10): 1122-3.
230. Li QH, Gao WJ, Li JY, et al. Detection of respiratory viruses in children with acute lower respiratory tract infection: an analysis of 5150 children. *Chin J Contemp Pediatr* 2016; **18**(1): 51-4.
231. Xie F, Cai YY, Yang L, Yue B. Study on viral pathogen of acute respiratory infection of 741 children. *China Journal of Child Health Care* 2015; **23**(2): 1104-6.
232. Xie ZC, Zhang YM. Characteristics of respiratory syncycial virus associated lower respiratory infection in hospitalised children in People's Hospital in Dongguan. *Anhui Medical and Pharmaceutical Journal* 2016; **20**(1): 139-40.
233. Singleton RJ, Bulkow LR, Miernyk K, et al. Viral respiratory infections in hospitalized and community control children in Alaska. *J Med Virol* 2010; **82**(7): 1282-90.
234. Huo X, Fang B, Liu L, et al. Clinical and epidemiologic characteristics of respiratory syncytial virus infection among children aged <5 years, Jingzhou City, China, 2011. *J Infect Dis* 2013; **208 Suppl 3**: S184-8.
235. Chi H, Chang IS, Tsai FY, et al. Epidemiological study of hospitalization associated with respiratory syncytial virus infection in Taiwanese children between 2004 and 2007. *J Formos Med Assoc* 2011; **110**(6): 388-96.
236. Mori M, Kawashima H, Nakamura H, et al. Nationwide survey of severe respiratory syncytial virus infection in children who do not meet indications for palivizumab in Japan. *J Infect Chemother* 2011; **17**(2): 254-63.
237. El Kholy AA, Mostafa NA, El-Sherbini SA, et al. Morbidity and outcome of severe respiratory syncytial virus infection. *Pediatr Int* 2013; **55**(3): 283-8.
238. Khuri-Bulos N, Williams JV, Shehabi AA, et al. Burden of respiratory syncytial virus in hospitalized infants and young children in Amman, Jordan. *Scand J Infect Dis* 2010; **42**(5): 368-74.
239. Roglic S, Knezovic I, Markovinovic L, Mise B, Tesovic G. Bronchiolitis caused by respiratory syncytial virus in the period from 2003 to 2009 [Croatian] (Bronhiolitis uzrokovan respiratornim sincicijskim virusom u razdoblju od 2003. do 2009). *Infektoloski Glasnik* 2009; **29**(3): 101-10.
240. Greenberg D, Dagan R, Shany E, Bar-Ziv J, Givon-Lavi N. Increased risk for respiratory syncytial virus-associated, community-acquired alveolar pneumonia in infants born at 31-36 weeks of gestation. *Pediatr Infect Dis J* 2014; **33**(4): 381-6.
241. Savic N, Jankovic B, Minic P, et al. Clinical characteristics of respiratory syncytial virus infection in neonates and young infants. *Vojnosanit Pregl* 2011; **68**(3): 220-4.
242. Duppenthaler A, Gorgievski-Hrisoho M, Frey U, Aebi C. Two-year periodicity of respiratory syncytial virus epidemics in Switzerland. *Infection* 2003; **31**(2): 75-80.
243. Berner R, Schwoerer F, Schumacher RF, Meder M, Forster J. Community and nosocomially acquired respiratory syncytial virus infection in a German paediatric hospital from 1988 to 1999. *Eur J Pediatr* 2001; **160**(9): 541-7.
244. Simon A, Ammann RA, Wilkesmann A, et al. Respiratory syncytial virus infection in 406 hospitalized premature infants: results from a prospective German multicentre database. *Eur J Pediatr* 2007; **166**(12): 1273-83.
245. Numa A. Outcome of respiratory syncytial virus infection and a cost-benefit analysis of prophylaxis. *J Paediatr Child Health* 2000; **36**(5): 422-7.

246. Bockova J, O'Brien KL, Oski J, et al. Respiratory Syncytial Virus Infection in Navajo and White Mountain Apache Children. *Pediatrics* 2002; **110**(2 Pt 1): e20.
247. Carballal G, Videla CM, Espinosa MA, et al. Multicentered study of viral acute lower respiratory infections in children from four cities of Argentina, 1993-1994. *J Med Virol* 2001; **64**(2): 167-74.
248. Vieira SE, Stewien KE, Queiroz DA, et al. Clinical patterns and seasonal trends in respiratory syncytial virus hospitalizations in Sao Paulo, Brazil. *Rev Inst Med Trop Sao Paulo* 2001; **43**(3): 125-31.
249. Miranda-Novales G, Solorzano-Santos F, Leanos-Miranda B, Vazquez-Rosales G, Palafox-Torres M, Guiscafre-Gallardo H. Blood culture and respiratory syncytial virus identification in acute lower respiratory tract infection. *Indian J Pediatr* 1999; **66**(6): 831-6.
250. Byington CL, Wilkes J, Korgenski K, Sheng X. Respiratory syncytial virus-associated mortality in hospitalized infants and young children. *Pediatrics* 2015; **135**(1): e24-31.
251. Vizcarra-Ugalde S, Rico-Hernandez M, Monjaras-Avila C, et al. Intensive care unit admission and death rates of infants admitted with respiratory syncytial virus lower respiratory tract infection in Mexico. *Pediatric Infectious Disease Journal* 2016; **35**(11): 1199-203.
252. Ng KF, Tan KK, Sam ZH, Ting GS, Gan WY. Epidemiology, clinical characteristics, laboratory findings and severity of respiratory syncytial virus acute lower respiratory infection in Malaysian children, 2008-2013. *Journal of Paediatrics and Child Health* 2016.
253. Gupta P, Beam BW, Rettiganti M. Temporal Trends of Respiratory Syncytial Virus-Associated Hospital and ICU Admissions Across the United States. *Pediatric Critical Care Medicine* 2016; **17**(8): E343-E51.
254. Liu L, Oza S, Hogan D, et al. Global, regional, and national causes of child mortality in 2000-13, with projections to inform post-2015 priorities: an updated systematic analysis. *Lancet* 2015; **385**(9966): 430-40.
